# *Alteromonas macleodii* breaches the diatom frustule triggering protoplast emergence and programmed necrosis-like death in *Thalassiosira rotula*

**DOI:** 10.1038/s41467-026-78079-7

**Published:** 2026-09-26

**Authors:** Isobel Short, Clara Martínez-Pérez, Samuel Miravet-Verde, Shinichi Sunagawa, Roman Stocker, Uria Alcolombri

**Affiliations:** 1 Institute of Environmental Engineering, Department of Civil, Environmental and Geomatic Engineering, Eidgenössische Technische Hochschule Zürich (ETHZ), Zürich, Switzerland; 2 Institute of Microbiology and Swiss Institute of Bioinformatics, Department of Biology, Eidgenössische Technische Hochschule Zürich (ETHZ), Zürich, Switzerland; 3 Department of Plant and Environmental Sciences, Institute of Life Sciences, The Hebrew University of Jerusalem, Jerusalem, Israel; 4 Present Address: Limnological Research Station, Department of Hydrology, University of Bayreuth, Bayreuth, Germany

**Keywords:** Water microbiology, Microbial biooceanography

## Abstract

Diatom-bacteria interactions drive biogeochemical fluxes at the base of the marine food-web. Although bacteria are increasingly recognised as important drivers of algal mortality, the diversity of algicidal strategies remains understudied. Here we show that *Alteromonas macleodii*, a recurrent member of *Thalassiosira rotula*’s microbiome, breaches the diatom’s silica frustule, inducing >95% of cells to emerge from their shells within 72 h, exposing protoplasts to lysis and exploitation. Whilst protoplast formation was induced by diverse bacteria, *A. macleodii* uniquely caused population-wide collapse, used here to characterise this diatom stress response. Dual transcriptomics indicated the presence of a programmed necrosis-like cell death pathway in diatoms underpinning *T. rotula*’s terminal morphological change, alongside upregulation of bacterial motility, chemotaxis and opportunistic metabolism. These findings reveal a potent algicidal strategy, recasting *A. macleodii* as an antagonist that exploits diatom mortality for its own benefit, removes the ballast provided by their frustules and thus reshapes oceanic carbon cycling.

## Abstract (web summary)

In this study, the authors show that the marine bacterium *Alteromonas macleodii* exploits the diatom *Thalassiosira rotula* by triggering programmed necrosis-like cell death. By forcing cells out of their silica shells and lysing them, this interaction may affect ocean carbon cycling.

## Introduction

Marine microbial ecosystems are composed of networks of interactions among species, which occur at the microscale yet influence biogeochemical cycles at global scales. Having cohabited the surface ocean for millions of years, diatoms and bacteria have co-evolved a strong ecological coupling^1^. Diatom-bacteria relationships exist on a continuum from reciprocal to antagonistic interactions, encompassing mutualism, commensalism, parasitism and amensalism^1,2^. Interactions with bacterial partners influence diatom metabolism^3^, productivity^4^, cell morphology^5^, aggregation^6^, cell division^7^ and sexual reproduction^8^. By shaping the life histories of their algal hosts, bacterial-algal relationships affect primary productivity, microbial community dynamics and oceanic carbon cycling. Diatoms’ siliceous frustules ballast organic particles, enhancing carbon flux to depth and making diatom-derived carbon a major contributor to particulate organic carbon (POC) export^9^. While bacteria-driven aggregation can modulate carbon export^6^, interactions that disrupt frustule ballast may have similarly significant consequences, an effect that remains unexplored.

Bacteria most commonly associated with diatoms belong to only a few genera within the phyla Proteobacteria and Bacteroidetes^10^, highlighting the extensive specialisation required to establish stable interkingdom interactions. For example, to achieve favourable rates and durations of encounters with algae, bacteria have evolved targeted chemotaxis and attachment mechanisms^11–14^. Once the two actors are in close proximity, they are able to more effectively exchange the chemical currencies mediating their interaction, including micronutrients such as vitamins^15^ and iron^16^, along with nitrogen^17^ and dissolved organic carbon (DOC)^18,19^. These resource exchanges occur primarily within the phycosphere—the micro-environment enriched in algal-derived metabolites surrounding individual phytoplankton cells^2,20^—where they sustain individual partners and collectively shape the structure, productivity, and biogeochemical impact of marine microbial communities.

In the most extreme form of antagonism, some heterotrophic bacteria have evolved to secure nutrients by actively inducing phytoplankton cell death^21–23^. These algicidal strains deploy diverse mechanisms, inducing growth inhibition^7^, morphological changes^24^, and most dramatically, cell lysis^25^—processes that modulate microbial community dynamics and profoundly impact the balance between primary and secondary production in marine ecosystems^26^. Similar to viruses, which regulate microbial ecosystems by driving phytoplankton mortality and fuelling the viral shunt^27,28^, algicidal bacteria can disrupt bloom dynamics^21–23,25^, yet their ecological roles, prevalence and antagonistic mechanisms remain poorly characterised. Moreover, phytoplankton cell death encompasses multiple distinct pathways, both programmed and passive, yet our understanding of their diversity and molecular hallmarks^29^ lags behind that of other eukaryotes such as mammals and yeast^30,31^. This is exemplified by the formation of protoplasts, a phenotype observed at low prevalence in nutrient-limited cultures of many diatom species^32^ (Supplementary Fig. 1) but never directly characterised, nor systematically investigated during interactions with antagonistic bacteria. Resolving these fundamentals of phytoplankton cell death is essential for understanding their impacts on microbial ecology and nutrient fluxes in the ocean.

Here, we report the identification and characterisation of a striking algicidal interaction between two globally distributed microplankton species, the diatom *Thalassiosira rotula*^33,34^ and the bacterium *Alteromonas macleodii*^35,36^. *A. macleodii* is emerging as a model marine heterotrophic bacteria, having evolved versatile copiotrophic strategies to thrive in nutrient-rich niches^37–44^. Members of the *Alteromonas* genus have been identified as recurrent and often dominant phycosphere companions of diatoms^45,46^, including as stable associates of *T. rotula* in previous studies^47–50^. Using time lapse microscopy, we observed that co-culture with the bacterium induces a terminal morphological change in the diatom, causing complete emergence of the protoplast from the silica shell and frequently leading to cell lysis; this interaction is contact dependent and results in rapid bacterial population growth. 14 of 24 additional taxonomically diverse marine bacteria induced protoplast formation in *T. rotula*, though not at the population-wide scale of *A. macleodii*. Employing a dual transcriptomic approach allowed us to follow the simultaneous gene expression changes in both species during co-culture, revealing that *A. macleodii* induces a stress response in *T. rotula* that leads to a programmed cell death (PCD) pathway, with genetic and phenotypic signatures of programmed necrosis. Bacterial gene expression analysis revealed that motility, chemotaxis and dynamic metabolic strategies underlie *A. macleodii*’s virulence and its ability to exploit diatom exudates and lysates, highlighting that traits previously considered opportunistic can also serve algicidal functions. Our results reveal a potent algicidal mechanism that shapes phytoplankton and bacterial population dynamics and, by removing the ballast provided by diatom frustules, may alter the sinking speed of particles and fate of carbon in the ocean.

## Results

### *A. macleodii* triggers protoplast formation in *T. rotula*

**Fig. 1:**
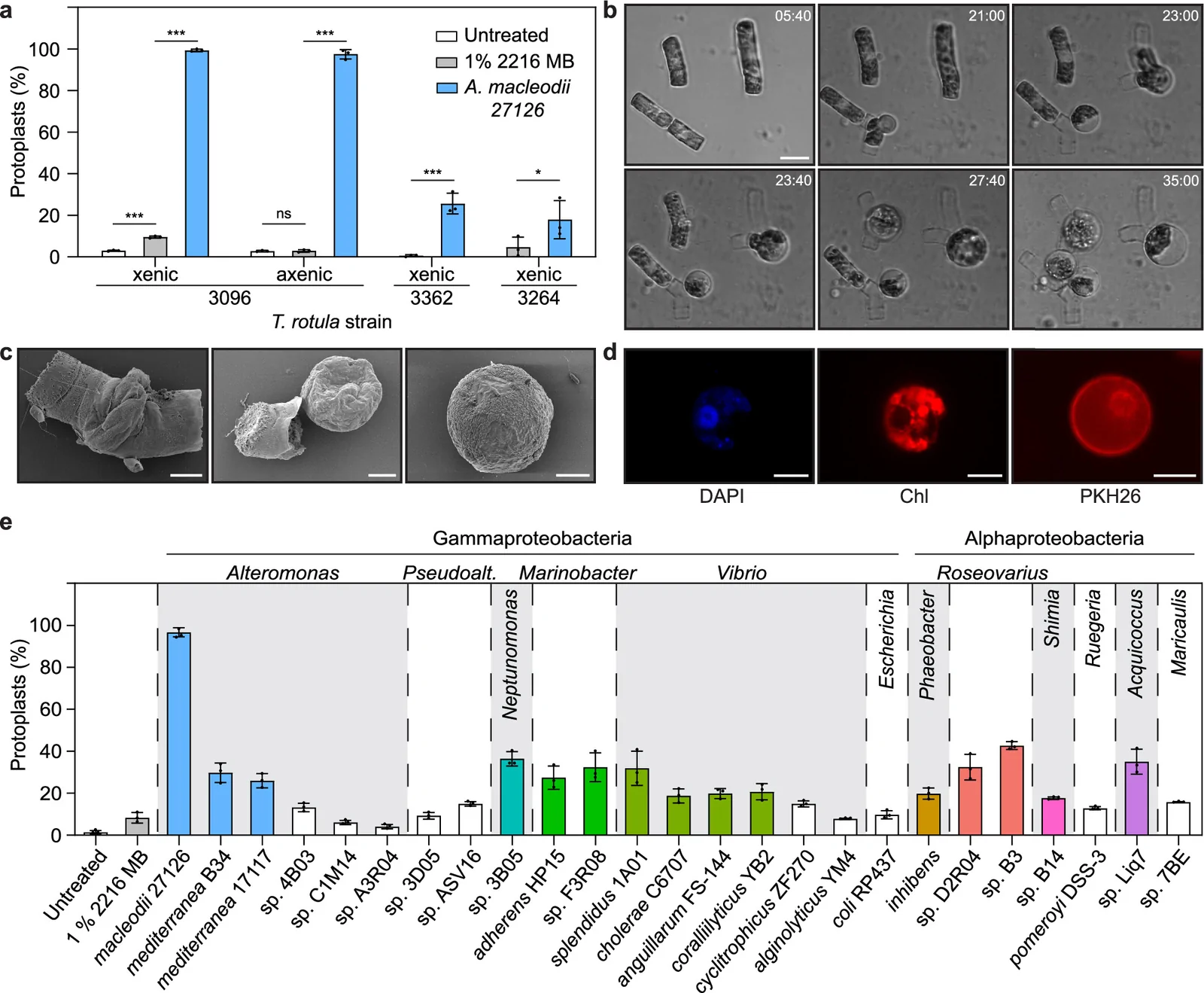
*A. macleodii* and diverse bacteria induce *T. rotula* to emerge from its protective silica frustule. **a** Proportion of protoplasts (% of total diatom cells) in three *Thalassiosira rotula* strains (CCMP 3362, 3264 and 3096; with and without their associated microbiomes) after 72 h of exposure to *Alteromonas macleodii* ATCC 27126, fresh bacterial culture medium (1% 2216 MB), or left untreated. Bars show means ± s.d. (n = 3 independent biological replicates), 100 diatom cells examined per replicate by phase contrast microscopy. Horizontal bars show statistical significance from within-strain one-way ANOVA with Tukey’s post-hoc test (CCMP 3096 xenic and axenic) or unpaired two-sided t-test (CCMP 3362 and 3264); *** = *P* < 0.001, * = *P* < 0.05 (*P* = 0.033), ns = not significant (*P* = 0.996). **b** Representative images from a time-lapse video of four *T. rotula* CCMP 3096 cells emerging from their silica frustules and forming protoplasts, in co-culture with *A. macleodii* ATCC 27126. Labels indicate time since the start of co-culture (hh:mm), scale bar 20 μm (Supplementary Video 1). **c** Scanning electron micrographs of co-cultures showing progressive formation of *T. rotula* protoplasts during co-culture. Left: membrane protrusion emerges between the thecae. Centre: protoplast released from the frustule. Right: detached protoplast. Scale bars 5 μm. **d** Fluorescence microscopy images of *T. rotula* protoplasts showing cellular arrangement. Left: DAPI staining of DNA. Centre: chlorophyll autofluorescence of the chloroplasts. Right: PKH26 staining of cell membrane lipids. Scale bars 10 μm. **e** Proportion of protoplasts (% of total diatom cells) in *T. rotula* CCMP 3096 after 72 h left untreated, exposed to fresh bacterial culture medium (1% 2216 MB), or to one of 24 bacterial strains, grouped and colour-coded by genus (order indicated). Full bacterial strain details and selection rationale are given in Supplementary Data 2. Bars show means ± s.d. (*n* = 3 independent biological replicates), 100 diatom cells examined per replicate by phase contrast microscopy. Coloured bars indicate a significant increase in protoplast formation relative to the 2216 MB control (one-way ANOVA with Dunnett’s post-hoc test; *P* < 0.05; full statistical details for all comparisons are provided in the Source Data file), white bars indicate no significant difference (*P* > 0.05). Source data are provided as a Source Data file.

To investigate their interaction, exponentially growing *T. rotula* CCMP 3096 was co-cultured with *A. macleodii* ATCC 27126 at a final bacterial concentration of 1 × 10⁶ cells/mL and a bacteria-to-diatom cell ratio of 10:1 (Methods). Within 72 h of co-culture, 99.3% ± 0.6% of the diatom cells had undergone a striking morphological change, significantly more than the 3% ± 0.3% in control cultures without *A. macleodii* (mean ± SD, *n* = 3; one-way ANOVA with Tukey’s post-hoc test, *q*(6) = 344.9, mean difference = 96.37%, 95% CI [95.16, 97.59], *P* < 0.001; Fig. 1a). During the morphological transformation, cylindrical cells bent at the midpoint as a membrane protrusion formed at the thecae junction (Fig. 1b) and expanded within hours until the cell fully emerged from its silica frustule, resulting in a spherical protoplast (Fig. 1b, c, Supplementary Video 1). Protoplasts retained intact nuclei and chloroplasts (Fig. 1d). As protoplast formation was initially observed in xenic *T. rotula*, we repeated the co-culture experiments with an axenic culture lacking their associated microbiome, observing 97.4% ± 2.3% of diatom cells forming protoplasts after 72 h, consistent with xenic co-cultures and significantly more than the untreated control (one-way ANOVA with Tukey’s post-hoc test; *q*(6) = 119.4, mean difference = 94.56%, 95% CI [91.12, 98.00], *P* < 0.001; Fig. 1a). Addition of *A. macleodii* from exponential-phase cultures resulted in 1% (v/v) 2216 MB medium carryover into co-cultures (L1 medium in artificial seawater), introducing DOC concentrations approaching the upper end of those documented in productive coastal and bloom conditions in the ocean (Supplementary Text). Testing the effect of 1% 2216 MB medium alone resulted in significantly fewer protoplasts than the addition of *A. macleodii* (*q*(6) = 321.5, mean difference = 89.83%, 95% CI [88.62, 91.05], *P* < 0.001; Fig. 1a): resulting in 9.5% ± 0.5% in xenic cultures (significantly more than the no-treatment control; *q*(6) = 23.41, mean difference = 6.54%, 95% CI [5.33, 7.75], *P* < 0.001) and 2.9% ± 0.6% in axenic cultures (not significantly different from the no-treatment control; *q*(6) = 0.122, mean difference = 0.097%, 95% CI [−3.53, 3.34], *P* = 0.996). These results demonstrate that *A. macleodii* induces near-complete protoplast formation in *T. rotula*, and that the observed response cannot be attributed to DOC carryover or the diatom’s microbiome alone.

Protoplast formation in response to bacteria is restricted to *T. rotula* among the diatoms tested, yet inducible by a broad range of bacterial taxa. Co-culture with *A. macleodii* ATCC 27126 induced protoplast formation across all three *T. rotula* strains tested (Fig. 1a): CCMP 3362 and 3264 averaged 25.6% ± 5.0% and 17.9% ± 9.2% of cells forming protoplasts after 72 h, respectively, both significantly above the 2216 MB control (unpaired two-sided t-test; CCMP 3362: *t*(4) = 8.549, mean difference = 25.02%, 95% CI [16.89, 33.14], *P* < 0.001; CCMP 3264: *t*(4) = 3.213, mean difference = 13.46%, 95% CI [1.827, 25.09], *P* = 0.033) but substantially lower than the 99.3% ± 0.6% observed in CCMP 3096. In contrast, *A. macleodii* ATCC 27126 induced no morphological changes upon co-culture with *Thalassiosira weissflogii* CCMP 1050, *Thalassiosira pseudonana* CCMP 3367 or *Phaeodactylum tricornutum* CCMP 2560 (Source Data file), indicating that *T. rotula* is uniquely sensitive among the species tested here. To investigate the bacterial specificity of the interaction, we co-cultured *T. rotula* CCMP 3096 with a phylogenetically broad suite of 24 additional bacteria strains, spanning 12 genera across the Alpha- and Gammaproteobacteria (see Supplementary Data 2 for strain identity, isolation source and selection rationale). Whilst 14 of these 24 strains induced a significant increase in protoplast formation after 72 h relative to the 2216 MB control (one-way ANOVA with Dunnett’s post-hoc test; *P* < 0.05; test statistics, effect sizes and exact adjusted *P*-values provided in the Source Data file), no strain approached the potency of *A. macleodii* ATCC 27126: The next highest, a *Roseovarius* sp. (Alphaproteobacteria), induced protoplast formation in only 42.7% ± 1.8% of diatom cells (Fig. 1e). This demonstrates that phylogenetically diverse bacteria can trigger the response, and that induction cannot be predicted from phylogeny: within the *Alteromonas* alone, three strains caused no significant increase (*P* > 0.05), whilst three others induced significant protoplast formation (*P* < 0.001; full statistical details provided in the Source Data file). Together, these results identify *T. rotula* as uniquely sensitive to bacterial-induced protoplast formation, a response triggered by taxonomically diverse bacteria but with highly variable potency. As the strongest effect was observed between *T. rotula* CCMP 3096 and *A. macleodii* ATCC 27126, this pairing was used for subsequent experiments.

To evaluate the environmental plausibility of this interaction, we examined the co-occurrence of *T. rotula* and *A. macleodii* in environmental amplicon sequencing datasets. Mining the Microbe Atlas Project^51^ (MAP, >2 million samples) revealed co-detection of *Alteromonas* and *Thalassiosira* in 990 samples across diverse marine biomes (Supplementary Fig. 2a), significantly exceeding chance expectation (Fisher’s Exact Test; Odds Ratio = 3.63, 95% CI [3.40, 3.87], *P* = 1.19^−235^). Encounter rate estimates (Supplementary Text) predict on the order of 10–100 physical contacts with *A. macleodii* per *T. rotula* cell per day, when both taxa are co-detected at bloom-relevant cell concentrations (10²–10³ *T. rotula* cells/mL^34,52^; 10⁴–10⁵ *A. macleodii* cells/mL^36,53^), supporting the plausibility of microscale interactions between these organisms in situ. Consistent with an ecological association, the relative abundances of *Thalassiosira* and *Alteromonas* were positively correlated among 252 globally distributed samples meeting minimum read-count thresholds (Supplementary Fig. 2b), exceeding both bacterial and eukaryotic background correlation distributions (Supplementary Fig. 2c, d). The *T. rotula–A. macleodii* co-culture, therefore, serves as an ecologically grounded model system for investigating bacterially-induced protoplast formation.

### Contact-dependent algicidal activity of *A. macleodii* against *T. rotula*

**Fig. 2:**
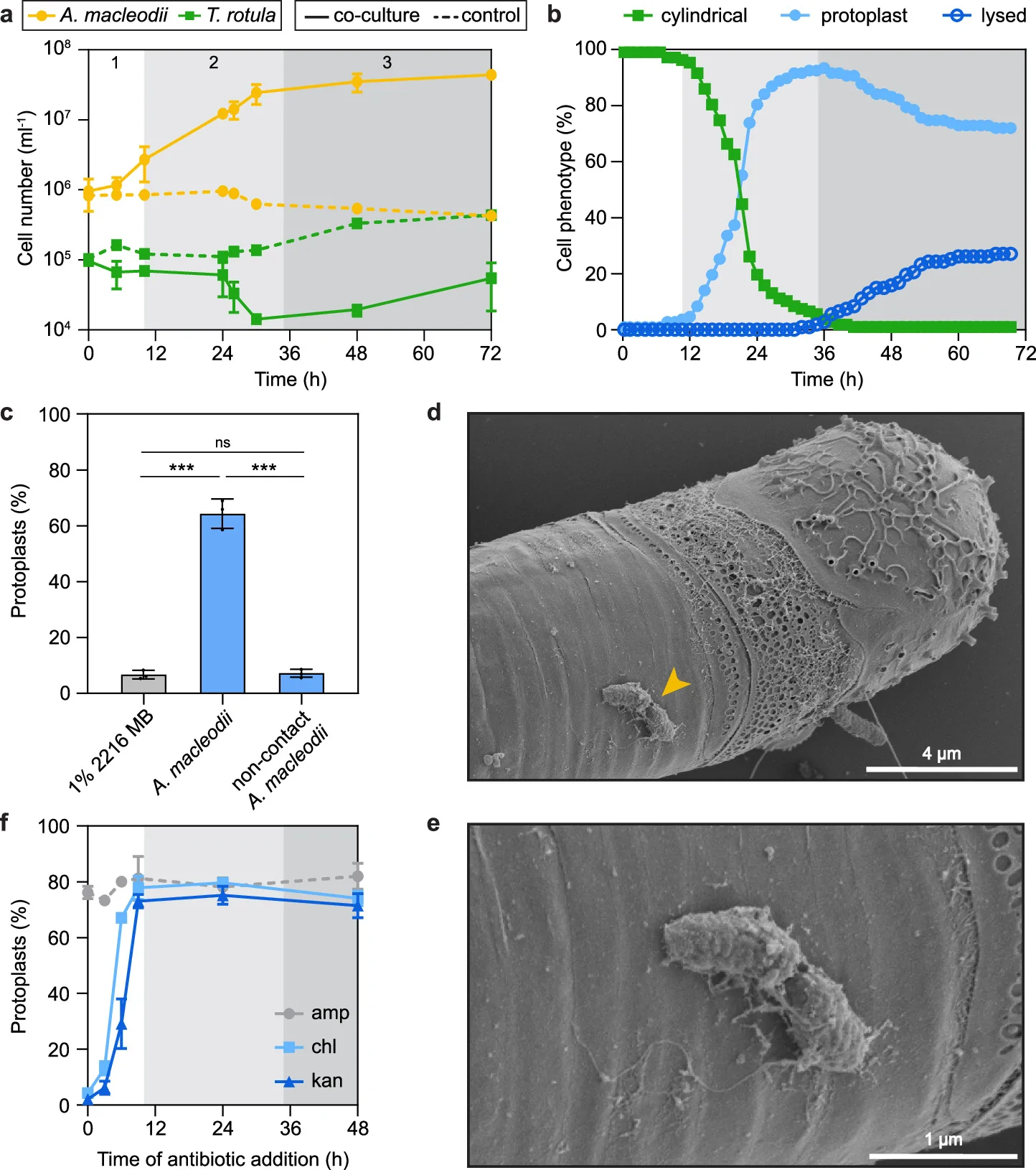
The antagonistic interaction between *T. rotula* and *A. macleodii* requires direct contact and occurs rapidly and irreversibly. **a** Population size over time for *A. macleodii* and *T. rotula* in co-culture and when cultured alone in diatom culture medium (“control”). Symbols represent mean ± s.d. (*n* = 3 independent biological replicates). Shaded regions denote the three phases of the interaction: Phase 1 (0–10 h) is associated with cessation of cell division in *T. rotula*; Phase 2 (11–35 h) coincides with the maximum growth rate of *A. macleodii*; and Phase 3 (36–72 h) involves lysis of *T. rotula* cells. **b** Representative experiment showing the proportion of *T. rotula* cells exhibiting cylindrical, protoplast or lysed phenotypes over time during co-culture with *A. macleodii*, as determined by video microscopy tracking of 100 individual *T. rotula* cells over the 72 h co-culture period. *T. rotula* cells undergo the morphological transition during Phase 2 and lysis begins in Phase 3. **c** Protoplast formation (% of diatom cells) in *T. rotula* after 72 h of culture with 1% 2216 MB (negative control), in co-culture with *A. macleodii*, or in co-culture with *A. macleodii* in shared medium with physical contact prevented by a filter (“non-contact *A. macleodii*”; Methods). Grey bar indicates the negative control; blue bars indicate conditions containing *A. macleodii*. Bars show mean ± s.d. (*n* = 3 independent biological replicates), 100 cells counted per replicate. Horizontal bars indicate statistical significance from one-way ANOVA with Tukey’s post-hoc test; *** = *P* < 0.001, ns = not significant (*P* = 0.984). Full statistical details are provided in the Source Data file. **d** Scanning electron micrograph of a co-culture fixed after 5 h, showing *A. macleodii* on the surface of a *T. rotula* cell. Scale bar 4 µm. **e** Magnified view of d. Scale bar 1 µm. **f** Protoplast formation (% of diatom cells) in *T. rotula* after 72 h of co-culture with *A. macleodii*, following the addition of ampicillin (amp), chloramphenicol (chl), or kanamycin (kan) after 0, 3, 6, 9, 24 or 48 h. Symbols show mean ± s.d. (*n* = 3 independent biological replicates), 100 diatom cells counted per replicate. Source data are provided as a Source Data file.

Time-lapse microscopy and flow cytometry revealed three distinct phases in the *T. rotula*–*A. macleodii* interaction (marked in Fig. 2a, b, f). Phase 1 (0-10 h) involved cessation of cell division in *T. rotula*, with no cell divisions observed by time-lapse microscopy (Fig. 2a, b). In Phase 2 (11–35 h), *A. macleodii* reached maximum growth (doubling time 5.9 ± 1.1 h; Fig. 2a) as diatom cells rapidly transformed into protoplasts, with >50% of diatom cells evacuating their silica shell by 20 h and 95% after 35 h (Fig. 2b). This transformation was irreversible: we never observed protoplasts regaining the cylindrical morphology or forming new frustules. Instead, protoplasts frequently underwent lysis, starting from 36 h after initiation of co-culture and defining Phase 3. By 72 h of co-culture, when the experiment ended, 27% of the protoplast diatom cells had lysed (Fig. 2b).

The antagonistic interaction required direct contact. No significant increase in the prevalence of protoplasts was observed in *T. rotula* when the two organisms were separated by a 0.45 μm membrane (“non-contact”), compared to the 2216 MB negative control (one-way ANOVA with Tukey’s post-hoc test**;** *q*(6) = 0.244, mean difference = 0.46%, 95% CI [−7.74, 8.66], *P* = 0.984; Fig. 2c). Furthermore, scanning electron microscopy (SEM) showed bacteria attached to diatom cells during Phase 1, already after 5 h of co-culture (Fig. 2d, e). Chemotaxis of *A. macleodii* toward exudates of *T. rotula* (Supplementary Fig. 3) suggests that such directed motility may facilitate the rapid establishment of physical contact.

*T. rotula*’s fate is determined within 9 h of exposure to *A. macleodii*. Antibiotic inhibition of protein synthesis in *A. macleodii* with chloramphenicol (“chl”) or kanamycin (“kan”) showed that commitment to the morphological change in *T. rotula* occurs during Phase 1—before protoplast formation in Phase 2—and is irreversible even if *A. macleodii* is subsequently inhibited. While antibiotic inhibition before 9 h produced a marked reduction in the proportion of protoplasts at 72 h relative to the negative control ampicillin (“amp”, to which *A. macleodii* is resistant), addition at or after 9 h yielded values comparable with the control (Fig. 2f).

### Protoplast formation in *T. rotula* is linked to programmed necrosis-like cell death

**Fig. 3:**
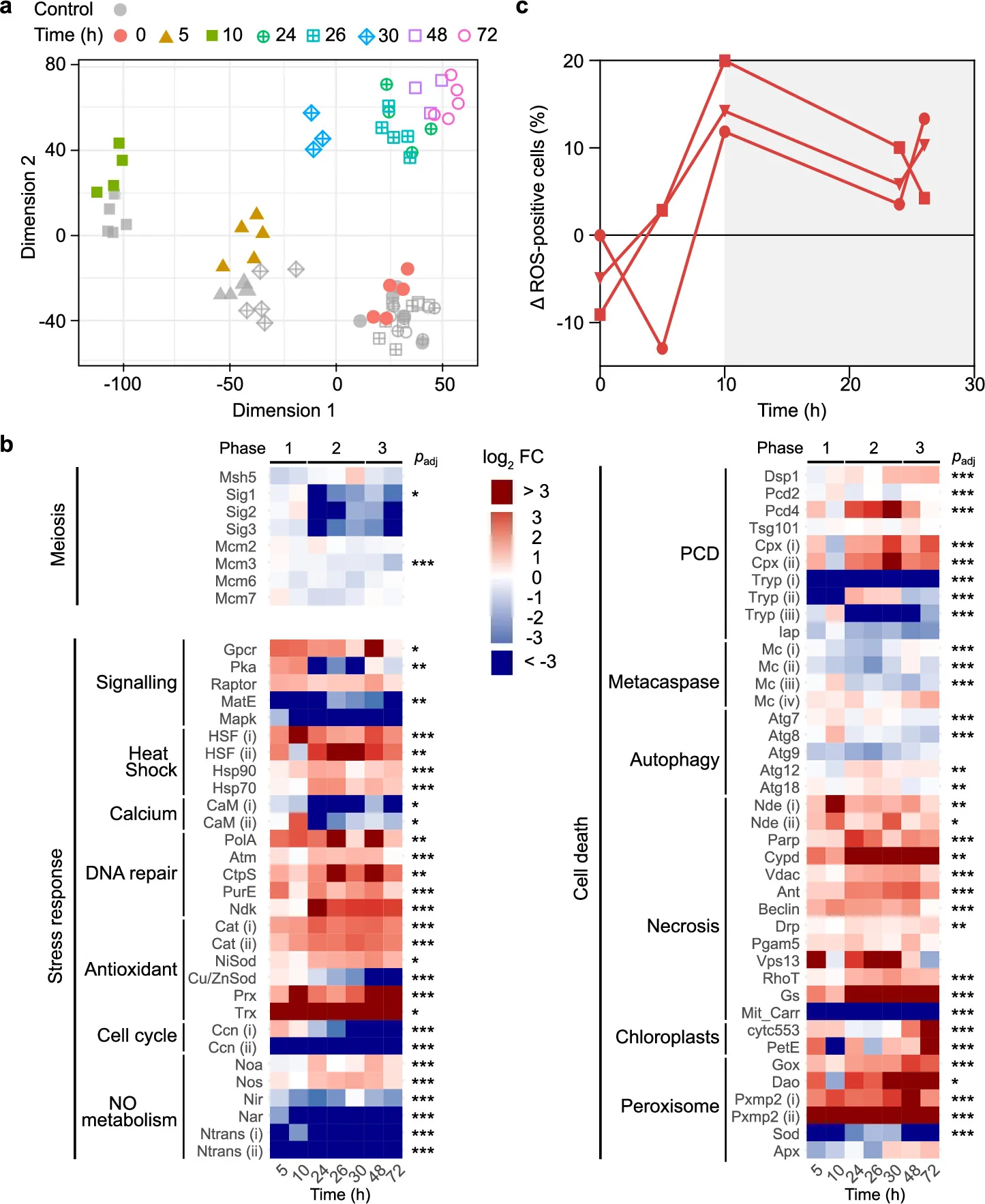
*T. rotula* in co-culture with *A. macleodii* displays transcriptomic signatures of cell death consistent with ROS-driven programmed necrosis. **a** Multidimensional scaling (MDS) plot based on variance-stabilising transformed (VST) RNA-seq data for *T. rotula* from five independent co-cultures with *A. macleodii* and five independent control cultures (treated with 1% 2216 MB). Each symbol represents a biological replicate; symbol shape indicates the timepoint, with control samples shown in grey and co-culture samples in the corresponding timepoint colour (see legend). **b** Relative transcript abundances of *T. rotula* genes after 5, 10, 24, 26, 30, 48, and 72 h, given as log_2_-fold change (log_2_FC) relative to the 0 h timepoint and control cultures (colour scale: ≤ –3 = dark blue, −3 to 0 = blue to white, 0 to 3 = white to red, ≥3 = dark red). Representative genes were selected based on their potential functional relevance to the phenotype, grouped by cellular process (KEGG annotations and manual curation); complete differential expression results are provided in Supplementary Data 4. Gene annotations are based on the *T. rotula* transcriptome annotation, supplemented in selected cases by targeted BLAST searches against other diatom homologues (Methods); full annotation and BLAST details are provided in Supplementary Data 4, 5 and 6. Asterisks indicate the significance of changes in expression over the time series (DESeq2 two-sided likelihood ratio test, * = *P*_adj_ < 0.05, ** = *P*_adj_ < 0.01, *** = *P*_adj_ < 0.001). Benjamini–Hochberg false discovery rate-adjusted *P*-values for all genes are provided in Supplementary Data 4. **c** Difference in the proportion of ROS-positive *T. rotula* cells at 0, 5, 10, 24, 26 h in co-culture with *A. macleodii* in comparison to control cultures (no treatment), measured by CM-H₂DCFDA staining. Symbols show independent biological replicates, >10,000 cells counted per replicate by flow cytometry. Source data are provided as a Source Data file.

To understand the responses of *T. rotula* and *A. macleodii* at the level of gene expression during their interaction, we conducted dual transcriptomic sequencing of co-cultures and controls at 8 timepoints over 72 h (0, 5, 10, 24, 26, 30, 48, 72 h), with 5 biological replicates per timepoint. The de novo assembled transcriptome of *T. rotula* contained 35,541 unique predicted coding sequences (CDS), of which 14,335 genes (40%) were differentially expressed in co-culture relative to the negative controls (two-sided likelihood ratio test; adjusted *P* < 0.05; Methods). Multivariate analysis showed samples clustered by timepoint, with biological replicates grouping closely together, indicating a reproducible progression of the transcriptomic response (Fig. 3a). A diurnal pattern was evident in both control and co-culture samples, as illustrated by the clustering of 0, 24, 48 and 72 h control samples, collected at the same phase of the 12:12 light:dark cycle. During the first 10 h (Phase 1), gene expression profiles were similar in co-cultures and controls, while they diverged markedly from 24 h onwards (Phase 2), coinciding with the phenotypic change in the diatom population.

Given the morphological similarity between protoplasts and auxospores, we examined the transcription of markers of diatom sexual production^54–60^ (Supplementary Text), and found they were not differentially expressed or significantly downregulated in co-culture (Fig. 3b), indicating sexual reproduction pathways do not underlie the observed morphology.

The transcriptomic results suggest that *T. rotula* rapidly initiates a stress response upon co-culture with *A. macleodii*, sequentially activating signalling and repair pathways. Major transcriptional changes were detected as early as 5 and 10 h (Phase 1) and persisted, including upregulation of signalling proteins such as Class 3 G protein-coupled receptor, *Gpcr (C3)*, proteins involved in the TORC complex (*Raptor*), and the kinase *Pka*, alongside strong downregulation of *Mapk*, inositol phosphate signalling components (*Pld*), sensor histidine kinases (*HK)* and *MatE* (Fig. 3b): repression of *MatE* may reduce signalling molecule export to reinforce stress signalling^61,62^. Heat shock factors (*HSF*), key stress regulators, and effector heat shock protein *Hsp90* were upregulated within 5 h, followed by *Hsp70* after 24 h, indicating a phased stress and protein homoeostasis programme. Calmodulin (*CaM)* genes were strongly downregulated from Phase 2, suggesting repression of Ca²⁺-dependent signalling. Genes for DNA damage repair and antioxidants to neutralise reactive oxygen species (ROS) were sequentially activated during Phases 1 and 2. Among the most strongly upregulated genes was a peroxiredoxin (*Prx*), which increased more than twentyfold by 10 h and over fiftyfold by 72 h, consistent with *Prx* induction to detoxify H₂O₂ reported in *Thalassiosira pseudonana*^63^. The strong downregulation of two G1/S Cyclin (*Ccn*) homologues from 5 h and 30 h, may indicate cell cycle arrest, though the expansion of the cyclin family in diatoms^64^ and the absence of functional characterisation in *T. rotula* limits the specificity of this interpretation. These transcriptional patterns reflect a rapid response to *A. macleodii* and sequential activation of stress-signalling, antioxidant defences and DNA/protein repair pathways, promoting stress acclimation and survival.

From Phase 2 onwards, coinciding with the observed morphological change, *T. rotula*’s transcriptome showed signatures of phytoplankton programmed cell death (PCD)^29,63,65–67^, indicating that sustained stress overcame acclimation and shifted cell fate. Upregulated genes included dual stress/PCD regulators *Pcd4*, Ca²⁺-binding *Dsp1*, *Hsp90*, and *Prx*, and a number of cysteine-endopeptidases that help to execute PCD (Fig. 3b): Among these was a cathepsin, *Cpx* (i), with 50% amino acid sequence identity (AA id) to the PCD-linked *P. tricornutum* Cpx^65^, and 38% AA id to human cathepsin Z, an executioner of the programmed necrosis pathway^68^. We also observed upregulation of *Cpx* (ii), showing 31% AA id to a human cathepsin L, which encodes an I_29 autoinhibitory domain common in regulated proteases associated with PCD. Moreover, expression of BIRC2-like inhibitor-of-apoptosis (*Iap*), which restrains cysteine endopeptidases, was significantly downregulated^63^. While several trypsin proteases linked to cell death in *T. pseudonana*^69^ were downregulated, this may indicate that protein catabolism becomes selectively regulated during the execution of cell death.

Expression of cell death markers did not align with the metacaspase-dependent or autophagy-dependent PCD pathways previously characterised in phytoplankton. Metacaspase enzymes (*Mc*)^70–74^, autophagy genes^75^, and genes whose expression is correlated with PCD in *Skeletonema marinoi*, *Pcd2* and *Tsg101*^67^ (Fig. 3b), showed weak or non-significant expression changes. Moreover, we found no clear evidence for nitric oxide (NO) signalling, a key regulator of these PCD pathways in other algae^76^ (Supplementary Text). These findings suggest *T. rotula* in co-culture with *A. macleodii* undergoes a form of PCD that is distinct from the metacaspase-, NO- and autophagy-dependent pathways studied in phytoplankton to date.

*T. rotula*’s PCD process displayed transcriptional signatures of mitochondrial ROS-linked PCD pathways, particularly programmed necrosis characterised in other eukaryotes but not previously described in algae^68,77,78^. Two alternative NADH dehydrogenases (*Nde*) were strongly upregulated at 10 h: *Nde* (i) (33% AA id to fungal *Nde-2*) increased by more than twentyfold at 10 h, and carries an EF hand domain, consistent with calcium regulation (Fig. 3b). *Nde* (ii) (36% AA id to yeast *Nde1* and to fungal *Nde-2*) also peaked at 10 h, coinciding with the commitment of *T. rotula* to protoplast-linked cell death. In budding yeast, Nde1 acts as a death effector^79^ and Nde2 in the fungus *N. crassa* directly generates mitochondrial ROS and promotes cell death^80^. Alternative NADH dehydrogenases are distant homologues of mammalian apoptosis-inducing factor (AIF), whose release from mitochondria triggers ROS-linked PCD pathways in mammals, including programmed necrosis^68,81,82^. Additional hallmarks of programmed necrosis in mammalian cells include PARP1 activation, which impairs respiration and promotes AIF release from mitochondria^83,84^, and the opening of the mitochondrial membrane permeability transition pore (mPTP), which further depolarises the mitochondria membrane, contributing to ATP depletion and ROS generation^77,85^. At 24 h, *T. rotula* upregulated transcription of a *Parp* family gene and the three components of the mPTP; *Vdac, Ant* and *Cypd*. Putative Nde and mPTP involvement parallels mitochondrial ROS-linked cell death and programmed necrosis pathways described in other eukaryotes^68,78^. Mitochondrial stress is further suggested by the induction of mitophagy regulators in *T. rotula* (*Beclin1*, *Drp, Pgam5, Rho, Vps)*^86^ and the sustained expression of glutamine synthetase (*Gs*), indicative of an attempt to mitigate redox imbalance. Transcriptomic signatures of mitochondrial collapse indicate that *T. rotula* is unable to sustain energy-dependent PCD pathways and undergoes a programmed necrosis-like form of cell death.

ROS production appears to precede and potentially trigger the cell death process in *T. rotula*, with peroxisomes likely amplifying mitochondrial dysfunction. Strong regulation of thylakoid membrane alternative photosystem I (PSI) electron donors *PetE* and *Cyt-553* may indicate chloroplast redox imbalances (Fig. 3b). Upregulation of *Dsp1*, previously linked to cyclic electron flow around photosystem I (PSI) during cell death in *T. pseudonana*^63,66,87^, further supports altered chloroplast ROS metabolism. H_2_O_2_-producing peroxisomal enzymes D-amino acid oxidase (*Dao*) and glycolate oxidase (*Gox*), which play a role in photorespiration and PCD in *S. marinoi*^67^, were upregulated from 5 h onward. Multiple *Pxmp*2 genes, encoding the H_2_O_2_-permeable peroxisomal membrane pore, were also strongly upregulated, with *Pxmp*2 (ii) expression remaining elevated more than eightfold at all timepoints. Concurrently, peroxisomal antioxidants such as *Sod*, *Prx* and ascorbate peroxidase (*Apx*) were not upregulated, suggesting elevated ROS production from peroxisomes and leakage into the cytosol. ROS staining with CM-H_2_DCFDA (Methods) showed that from 5 h of co-culture onwards, the proportion of ROS-positive *T. rotula* cells relative to the negative control was significantly greater than at 0 h (one-sided Wilcoxon signed-rank test; *W* = 74, *n* = 12, *P* = 0.0015, *r* = 0.859; Fig. 3c), further supporting the involvement of ROS in the protoplast phenotype in co-culture. Taken together, these results suggest that mitochondrial and peroxisomal-mediated oxidative stress drives a programmed necrosis-like cell death pathway in *T. rotula*, likely underlying the protoplast protrusion and loss of membrane integrity we have observed in co-culture with *A. macleodii*.

### Virulence strategies enabling nutrient extraction by *A. macleodii*

**Fig. 4:**
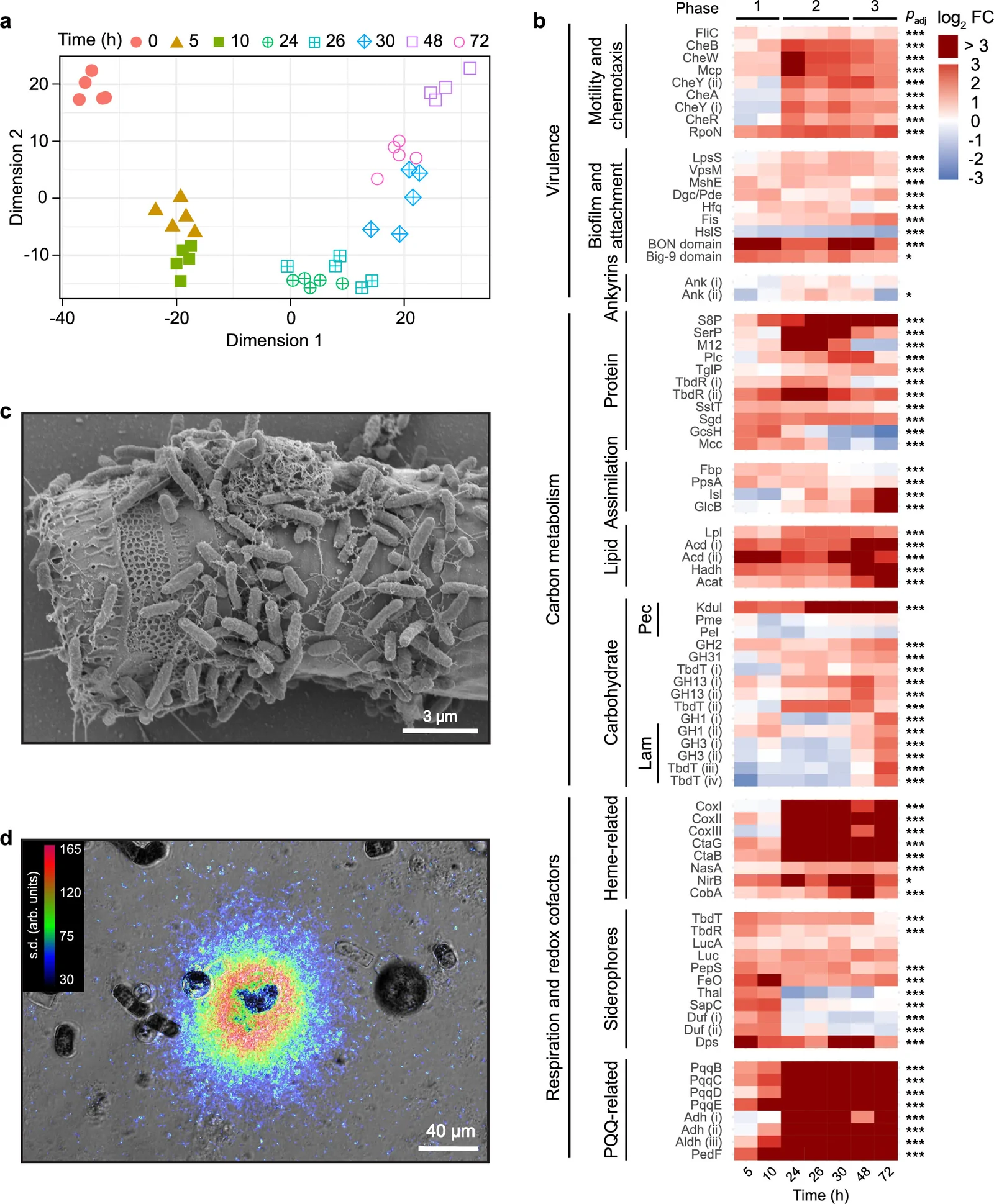
*A. macleodii*’s virulence strategy: coordination of motility, chemotaxis, biofilm formation and dynamic metabolism. **a** Multidimensional scaling (MDS) plot based on variance-stabilising transformed (VST) RNA-seq data for *A. macleodii* from five independent co-cultures with *T. rotula*. Each symbol represents a biological replicate; symbol shape and colour indicates the timepoint (see legend). **b** Relative transcript abundances of *A. macleodii* genes after 5, 10, 24, 26, 30, 48 and 72 h in co-culture, given as log_2_-fold change (log_2_FC) relative to the 0 h timepoint (colour scale: −3 to 0 = blue to white, 0 to 3 = white to red, ≥3 = dark red). Representative genes implicated in the bacterial response to co-culture and with potential relevance to the induction or exploitation of the protoplast phenotype are displayed, grouped by cellular process (KEGG annotations and manual curation); complete differential expression results and full annotation details are provided in Supplementary Data 7. Asterisks indicate significance of expression changes over the time series (DESeq2 two-sided likelihood ratio test, * = *P*_adj_ < 0.05, ** = *P*_adj_ < 0.01, *** = *P*_adj_ < 0.001); Benjamini–Hochberg false discovery rate adjusted *P*-values for all genes are provided in Supplementary Data 7. **c** Scanning electron micrograph of a co-culture fixed after 24 h, showing biofilm formation by *A. macleodii* on the surface of a *T. rotula* cell. Scale bar 3 µm. **d** Time-lapse video microscopy image showing *A. macleodii* swarming around a lysing *T. rotula* cell (Supplementary Video 3). The image is a Z-projection of the standard deviation of pixel intensity across 45 frames (1.8 s), used as a proxy for bacterial motility, false-coloured and overlaid on a single video frame. Scale bar 40 µm. Source data are provided as a Source Data file.

Of the 3740 genes transcribed by *A. macleodii* in co-culture, 2917 (78%) were differentially expressed relative to the 0 h timepoint (two-sided likelihood ratio test; adjusted *P* < 0.05; Methods), with distinct gene expression profiles at each timepoint (Fig. 4a). Upregulation was observed in genes involved in motility, chemotaxis, and biofilm formation—virulence pathways enabling host colonisation^2,88,89^. In the KEGG flagellar assembly and chemotaxis pathways, 86% and 89% of genes were differentially expressed, with strong upregulation peaking in Phases 2 and 3, as illustrated by representative genes in Fig. 4b. These include Sigma-54 (*RpoN*), a regulator of virulence and flagella synthesis^90^, which was consistently upregulated in co-culture. These transcriptomic patterns align with our evidence that *A. macleodii* exhibits chemotaxis towards chemical exudates from intact *T. rotula* cells (Supplementary Fig. 3) and lysed *T. rotula* cells (Fig. 4d), supporting an active chemotactic strategy for host location. 95% of biofilm- and attachment-related genes were differentially expressed, showing overall modest upregulation. Two of the most strongly upregulated genes at 5 h were a BON-domain protein (upregulated more than thirtyfold), which binds phospholipid membranes^91^, and a Big-9 domain protein (upregulated more than sixfold), implicated in adhesion to hosts including diatoms^92,93^ (Fig. 4b). The latter contains the COG2931 motif characteristic of Ca²⁺-binding RTX toxins, which show pore-forming and cytotoxic activity^94^. In agreement with these findings, phase contrast and scanning electron microscopy (SEM) revealed bacterial biofilm formation and extracellular matrix on and around diatom cells (Fig. 4c; Supplementary Video 2).

Gene expression data indicate that *A. macleodii*’s algicidal activity does not rely on canonical secretion pathways or common effectors, pointing to previously uncharacterised mechanisms of microbial interaction. Genes for the type II secretion system, Sec and Tat pathways, and major membrane vesicle proteins^95^ were not upregulated (Supplementary Data 8) and known eukaryotic-interference factors such as ankyrin repeat proteins^96^ exhibited minimal transcriptional changes (Fig. 4b). Extracellular proteases (*S8P*, *SerP, M12, Plc*; Fig. 4b), shown to be effectors in other algicidal interactions^11,97,98^, were only induced after 24 h, well after *T. rotula* cells were committed to the morphological change and many had already undergone the transformation (Fig. 4b). This suggests that proteases are likely not the causative agents of protoplast formation, instead acting saprotrophically, via extracellular digestion, to support *A. macleodii*’s growth.

*A*. *macleodii*’s transcriptome reveals dynamic, phase-dependent utilisation of algal-derived compounds, enabling it to exploit the enriched substrate pool created by *T. rotula*’s breakdown. *A. macleodii* initially exploited proteinaceous carbon, with upregulation of extracellular proteases, peptide and amino acid transporters (*TbdR, SstT*), and catabolism enzymes (*Sgd*, *GcsH*, *Mcc*) (Fig. 4b). Modest induction of gluconeogenic enzymes (*Fbp* and *PpsA*) between 5-26 h, indicates protein-derived carbon supported anabolic growth during this period. As *T. rotula*’s membranes were exposed and degraded during Phases 2 and 3, *A. macleodii* upregulated lipid catabolism, expressing lipases that liberate fatty acids (*Lpl*), β-oxidation (*Acd*, *Hadh*, *Acat)* and glyoxylate cycle enzymes (*Isl* and *GlcB)*, enabling the conservation of fatty-acid-derived carbon for biomass. *A. macleodii* utilised carbohydrates sparingly in co-culture overall (Supplementary Text; Supplementary Data 8) but upregulation of laminarin utilisation genes in Phase 3 is consistent with release of this storage glucan during *T. rotula* lysis, and indicative of a broader nutrient “feast” by 72 h (Fig. 4b). In parallel, *A. macleodii* leveraged redox cofactors to boost respiration of host-derived substrates, increasing investment in siderophores for iron acquisition (Supplementary Text), haem cofactor synthesis and haem-containing electron transport components. Furthermore, *A. macleodii* strongly induced synthesis of pyrroloquinoline quinone (PQQ), and PPQ-dependent alcohol dehydrogenases (*Adh*), coupling ATP production to oxidation of alcohols, including potentially methanol (Fig. 4b; Supplementary Text). Overall, *A. macleodii* dynamically adapted its metabolism to exploit the succession of diatom-derived substrates, culminating in a nutrient surge following *T. rotula* cell lysis.

## Discussion

### A potent algicidal strategy: exploiting protoplast formation

**Fig. 5:**
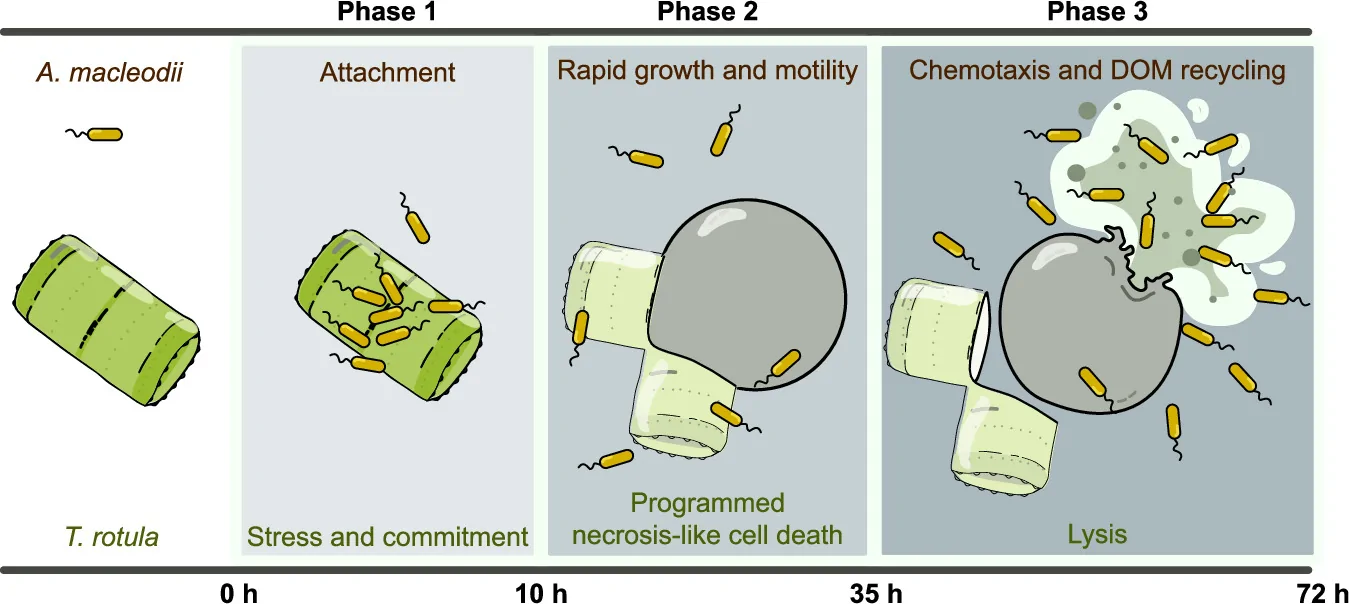
Conceptual model for the algicidal interaction between *T. rotula* and *A. macleodii.* The interaction unfolds in three distinct phases. In Phase 1 (0–10 h), *A. macleodii* attaches to *T. rotula*, triggering a transcriptional programme that includes stress-response genes and commitment to the terminal morphological change. Phase 2 (11-35 h) is marked by rapid bacterial population growth alongside protoplast emergence from the silica frustule, and the upregulation of diatom genes associated with programmed necrotic cell death, including peroxisomal and mitochondrial pathways leading to reactive oxygen species (ROS) accumulation. Meanwhile, in Phase 3 (after 35 h), the exposed protoplasts, lacking the protective silica shell, become susceptible to lysis. *A. macleodii* exhibits chemotactic swimming toward the extruded cellular material and upregulates genes involved in the degradation and assimilation of diatom-derived organic matter.

Our study characterises a distinctive antagonistic diatom-bacteria interaction. *Alteromonas macleodii*, a widespread heterotrophic bacterium whose genus co-occurs and co-varies with *Thalassiosira* across global ocean datasets, exhibits algicidal activity against *T. rotula* in co-culture, inducing over 99% of cells in the population to emerge from their protective silica shells, exposing vulnerable protoplasts, which are then exploited by the bacteria. This interaction progresses through three distinct phases (Fig. 5): *T. rotula* cells first cease to divide (0-10 h), then proceed through rapid and irreversible protoplast formation (11-35 h), and subsequent cell lysis (from 36 h). Concurrently, *A. macleodii* directly attaches to the diatom cells and proliferates rapidly, facilitated by chemotaxis toward and utilisation of *T. rotula*-derived metabolites. The high susceptibility of protoplasts to lysis, with 27% of the population lysed by 72 h, indicates that protoplast formation is a stress-induced, non-adaptive response, which *A. macleodii* exploits to fuel its own proliferation.

Comparable diatom protoplast morphologies occur under nutrient limitation and stress^32^ but have never been explicitly studied and have not previously been documented at the population-wide scale induced here by *A. macleodii*. In stationary-phase *T. rotula* CCMP 3096 cultures 20% of cells exhibited protoplast formation in the absence of *A. macleodii* (>10 days; Supplementary Fig. 1a). Protoplasts have also been noted at low prevalence in stationary-phase or stressed cultures of several other diatom species including *Coscinodiscus wailesii* CCMP 2513, *Thalassiosira pseudonana* CCMP 1335, *T. pseudonana* CCMP 3667, *Thalassiosira rotula* CCMP 3264, and *Thalassiosira weissflogii* CCMP 1051 (Supplementary Fig. 1b). These observations identify protoplast emergence as a general but poorly understood stress response, which *A. macleodii* may exploit and amplify to an extreme outcome to circumvent the diatom’s protective frustule. Bacterial induction of protoplast formation may represent a widespread but previously overlooked algicidal strategy; bacterial strains spanning 8 of 12 tested genera, including cosmopolitan diatom associated taxa^45^, induced significant protoplast formation in *T. rotula*. The broader prevalence of this phenomenon is further supported by recent evidence for protoplast formation in *Thalassiosira oceanica* by multiple *Ekhidna algicida* strains, isolated across the Pacific ocean^99^ (Supplementary Materials therein). Importantly, whereas stress-associated protoplast formation appears limited to a minority of cells, bacterial induction can affect a substantial fraction of the population, amplifying its ecological consequences. Stress accumulation, for example at the end of algal blooms^29^, may heighten this vulnerability in situ. Here we employ the *A. macleodii* - *T. rotula* pairing as a model system to investigate the ecologically relevant yet underexplored process of protoplast formation in diatoms.

To study this algicidal interaction, we established co-culture conditions that reliably synchronised protoplast formation in *T. rotula* after 72 h. Diatoms were co-cultured with *A. macleodii* at 1 ×10⁶ cells/mL (approximately a 1:10 diatom-to-bacteria ratio). While cell densities one to two orders of magnitude above typical environmental levels of both organisms^34,53^ were necessary to synchronise the population-level response and generate an interpretable transcriptomic signal, significant protoplast formation was still induced by lower *A. macleodii* starting concentrations (approximately 1×10^5^ cells/mL; Supplementary Fig. 4). This demonstrates that environmentally relevant bacterial abundances^53^ are sufficient to trigger this interaction, albeit affecting a smaller proportion of the diatom population. Such partial induction may favour coexistence over population collapse while still meaningfully influencing population dynamics and nutrient transfer to the heterotrophic community. In our experiments, bacterial inoculation introduced 1% (v/v) 2216 MB medium into the co-culture resulting in DOC concentrations within the range documented in productive coastal^100^ and bloom conditions^101^. Based on previous reports of DOM-activation of *A. macleodii*^11^, this may have stimulated its algicidal activity in co-culture. Indeed, addition of 1% 2216 MB alone to xenic *T. rotula* cultures significantly increased protoplast formation (Fig. 1a), though to a much lesser extent than addition of *A. macleodii*. The significant increase in protoplasts observed in xenic but not axenic cultures receiving 2216 MB addition suggests the phenotype results from DOC-mediated stimulation of resident bacteria. This includes *A. macleodii*, which was detected in our xenic diatom cultures by 16S rRNA analysis (Supplementary Data 1), consistent with previous reports of *Alteromonas* as a recurrent associate of *T. rotula*^47–49^. These findings highlight an environmentally relevant scenario in which DOM-enriched microenvironments during bloom conditions could activate resident bacteria to trigger protoplast formation in diatoms.

### Evidence for programmed necrosis-like PCD in *T. rotula*

Our results demonstrate that the death of *T. rotula* cells during co-culture with *A. macleodii* is a programmed response to prolonged stress, rather than a passive consequence of bacterial attack, as evidenced by commitment to protoplast emergence within 9 h, well before the morphological transformation begins, and the upregulation of a subset of PCD markers^29,63,67^. This provides direct evidence of PCD in *T. rotula* and underscores its role in bacteria-phytoplankton interactions^102^. While metacaspase homologues^70–73^ were not induced, *T. rotula* strongly upregulated a cathepsin *Cpx* and related proteases, whose homologues in *Phaeodactylum tricornutum* and *Chlamydomonas reinhardtii* mediate oxidative stress-induced PCD^65^. This remodelling of proteolytic transcription indicates activation of a redox-sensitive, metacaspase-independent pathway, thus distinct from those previously characterised in phytoplankton.

The programmed death of *T. rotula* exhibits phenotypic hallmarks of necrosis – perinuclear organelle clustering and plasma membrane rupture – together with genotypic signatures resembling programmed necrosis pathways described in other eukaryotes. The strong transcriptional spike of two alternative NADH dehydrogenase genes (*Nde*) at 10 h coincided with elevated ROS detected in *T. rotula* in co-culture, consistent with their role as mitochondrial ROS-generating death effectors in fungi^79,80^. As Nde proteins are distant homologues of the mammalian apoptosis-inducing factor (AIF), they have been proposed to serve as redox-regulated cell death switches, conserved across eukaryotes^78–80^. Therefore, the upregulation of *Nde* in *T. rotula* may suggest mechanistic similarities with AIF-mediated programmed necrosis studied in mammals^68,82^. In line with this interpretation, *T. rotula* exhibited additional hallmarks of this pathway^77,83,103^, including upregulation of *Parp*, components of the mPTP, markers of mitochondrial dysfunction, and evidence of both ROS and calcium involvement. In addition to mitochondrial ROS production, peroxisomal H₂O₂ production and chloroplast redox perturbations may contribute to oxidative stress amplification in this form of PCD, consistent with organelle-specific oxidative stress patterns regulating PCD in phytoplankton^104^. Calcium signalling, previously implicated in distinct phytoplankton PCD pathways^105^, is supported here by the induction of calcium-regulated genes in Phase 1, such as *Nde*(i)), and downregulation of calmodulins in Phase 2, which may reflect an adaptation to prolonged calcium signalling. Functional validation of the genes described here will be key to confirming and extending these findings. Overall, we present evidence for the existence of a programmed necrosis pathway in *T. rotula*, characterised by necrotic phenotypes and molecular parallels to the pathway documented in other eukaryotes. Protoplast formation observed here, and reported under stress in other diatom species, may represent a conserved morphological feature of this necrosis-like PCD. These findings add to evidence for non-apoptotic death programmes in algae^106,107^ and more broadly support the existence of a conserved programmed necrosis-like pathway with deep evolutionary origins in eukaryotes^78^.

### Mechanistic insights into *A macleodii*’s algicidal lifestyle

The induction of protoplast formation, likely representing necrosis-like PCD, by a wide diversity of bacteria suggests that rather than deploying dedicated algicidal effectors, these interactions proceed by indirectly overwhelming *T. rotula*’s latent stress pathways. Bacterial contributions to diatom stress may be multifactorial and strain-dependent, with multiple mechanisms potentially acting in concert even within a single strain. This may explain the difficulty in identifying a single effector responsible for *A. macleodii’s* algicidal activity, consistent with the lack of evidence for canonical mechanisms. The exceptional antagonistic potency of *A. macleodii* toward *T. rotula* may therefore relate more broadly to a suite of traits that mediate effective host colonisation and exploitation. Given the contact dependent nature of the interaction, motility and chemotaxis behaviours that enhance encounter probability, alongside attachment mechanisms that sustain physical contact, are likely key determinants of virulence. As such a particular gene of interest is the upregulated RTX-like toxin, implicated in adhesion to diatom hosts and cytotoxic activity^92,93^. These traits, combined with the metabolic flexibility to maximise exploitation of host resources, collectively underpin a coordinated attack-and-consume strategy, which may explain *A. macleodii*’s heightened antagonistic performance. The *A. macleodii* species complex displays remarkable divergence, ranging from opportunistic copiotrophy^36,39,41^ to beneficial associations that promote phytoplankton growth^108–111^. Our results suggest that the same toolkit that supports opportunistic scavenging and mutualistic association is here repurposed for host exploitation.

### Ecological implications

The presence of a programmed necrosis-like cell death pathway, involving protoplast emergence from the frustule and heightened vulnerability to lysis, has broad implications for our understanding of *T. rotula*’s life history and population dynamics. In turn, its induction by *A. macleodii* and potentially other bacteria, expands the ecological niche we ascribe to such organisms. Rather than passively scavenging the decay of a bloom, *A. macleodii* can be recategorized as an algicidal bacterium that actively generates sources of energy and carbon through host manipulation, potentially contributing to bloom termination. By impairing productivity, promoting diatom lysis and removing the ballast provided by the silica frustule, this interaction could reduce the export of carbon and other nutrients to deeper waters. *A. macleodii* has also been shown to form biofilm aggregates that reduce particle sinking and alter carbon export efficiency^112^, together suggesting this bacterium may reshape microbial communities and ocean biogeochemistry, paralleling the well-documented role of viruses^113–115^. Our findings underscore the ecological consequences of a distinctive algicidal adaptation in bacteria, highlighting the tight coupling between microbial interactions and the oceanic carbon cycle.

## Methods

### Diatom culture

*T. rotula* CCMP 3096, *T. rotula* CCMP 3362, *T. rotula* CCMP 3264, *Phaeodactylum tricornutum* CCMP 2560, *Thalassiosira pseudonana* CCMP 3367 and *Thalassiosira weissflogii* CCMP 1050 cells were obtained from NCMA (Bigelow Laboratory) as xenic cultures, with an associated bacterial community. All strains were maintained in L1-enriched (NCMA, Bigelow) autoclaved artificial seawater medium (35 g L^−1^ Instant Ocean, Spectrum Brands), with a 12:12 day:night cycle and diluted at least every 14 days, or more frequently as required. All strains were maintained at 14 °C, except *T. rotula* CCMP 3362 (maintained at 22 °C) and *P. tricornutum* (maintained at 18 °C).

### Cell counting

Algal density was measured using a flow cytometer (cytoFLEX, Beckman Coulter) with all gain and threshold settings on automatic. Gating was performed based on diatom cell autofluorescence in three different channels of excitation, APC (638 nm), PE (585 nm) and FITC (525 nm), plotted over forward scatter (FSC). The background signal from L1 medium within these gates was confirmed as zero, and the average cell count across the three channels was taken as the number of cells per mL.

### Fluorescent staining, microscopy, and flow cytometry

Cells were stained to visualise cell membranes with the lipophilic dye PKH26 (Sigma-Aldrich) by incubating cells with 1 μM of the original stock solution for 20 min in the dark. Chloroplasts were detected via chlorophyll autofluorescence without additional staining. To visualise nuclear DNA, cells were fixed with formalin at a final concentration of 4% 1 h before staining and then visualised using Fluoroshield with DAPI (Sigma-Aldrich), with cells incubated for 30 min in the dark before imaging. Fluorescence imaging was performed using a Nikon Ti-E; 20× NA 0.45 Objective, Sola Light Engine Lumencor.

To quantify reactive oxygen species (ROS) accumulation in diatom cells, CM-H₂DCFDA (Thermo Fisher Scientific) was added to cells at a final concentration of 5 μM and incubated for 1 h in the dark. Cells were then measured using a flow cytometer (CytoFLEX, Beckman Coulter) in the FITC-A channel (525/40 nm). The proportion of ROS-positive cells was determined in co-cultures and negative controls (no treatment), with the difference (ΔROS-positive) reported in the figure.

### Axenic cultures

Axenic *T. rotula* CCMP 3096 cultures were generated with the following protocol, which was verified to have no detrimental effect on diatom growth. A 5 mL sample of diatom culture in the exponential growth stage was filtered onto a 3 μm pore-size cell strainer (Sigma-Aldrich) and washed with 5 mL sterile L1 medium. 5 mL of L1 medium containing 20 µg/mL Triton X-100 detergent (Sigma-Aldrich) was added to the cells for 1 min, then the liquid was removed and the cells washed with 5 mL medium. Diatom cells were then aspirated off the membrane and diluted in 10 mL medium containing the antibiotics gentamycin sulfate 67 μg mL⁻¹, ciprofloxacin 20 μg mL⁻¹, chloramphenicol 25 μg mL⁻¹, ampicillin 10 μg mL⁻¹ and streptomycin sulfate 50 μg mL⁻¹ (all Sigma-Aldrich). Cells were incubated in this antibiotic-containing medium for 48 h under regular growth conditions and then transferred 1:10 to antibiotic-free medium. Bacterial decontamination was verified by transferring 100 μL of the diatom culture to 2 mL 2216 Marine Broth (BD Difco) and confirming that there was no change in OD600 after 24 h (CO8000 Cell Density Meter, LabGene scientific).

### Bacterial culture

A stock of *A. macleodii* ATCC 27126 was obtained from the ATCC culture collection and stored at −80 °C as a 20% glycerol stock. For experiments, 5 mL 2216 MB was inoculated from the glycerol stock and grown at 27 °C, 200 rpm for 12 h. Before addition to diatom cultures, the OD600 was measured using a spectrophotometer blanked with 2216 MB (CO8000 Cell Density Meter, LabGene scientific). All other bacterial strains were stored at −80 °C in 20% glycerol stocks, and were inoculated for experiments into 5 mL 2216 MB and grown at 27 °C, 200 rpm for 12 h.

### Cell counting

Bacterial density was measured using a flow cytometer (cytoFLEX, Beckman Coulter) after SYBR Green I staining (ThermoFisher) for 15 min in the dark. Bacteria were identified by gating on FITC-A fluorescence, plotted against forward scatter (FSC).

### Diatom and bacteria co-culture

The conditions for the addition of *A. macleodii* to *T. rotula* were optimised and these standards followed in all experiments, except where explicitly stated to differ for experimental purposes. *T. rotula* cultures split in L1 medium 3 days prior to use and with concentrations between 1 × 10^4^ and 20 × 10^4^ cells/mL were used to ensure exponential growth and optimal imaging. Exponentially growing *A. macleodii* cells, seeded 12 h prior to use, were added to a ratio of 1% (v/v) directly from the culture in 2216 MB. For co-culture with all other bacterial strains, cultures were normalised to 1 × 10^8^ cells mL⁻¹ in 2216 MB before addition at 1% v/v, resulting in a final bacterial concentration of 1 × 10^6^ cells mL⁻¹ in all co-cultures. Each experimental and control condition was conducted in triplicate, with each replicate culture in a separate well of the same 24-well glass-bottomed plates (Cellvis), allowing concurrent microscopic analyses to be performed directly. The plates were incubated at 14 °C with a 12:12 day:night cycle, and for end point experiments images were taken at 0 h and 72 h (10× Objective, Nikon ECLIPSE Ti Microscope equipped with Hamamatsu ORCAFlash4.0 digital camera). Multiple fields of view were imaged per well at random locations until ≥100 cells were captured, and all cells present in the captured images were counted. The most accurate method for analysing protoplast morphologies proved to be manual, thus *T. rotula* cell types were categorised and counted by eye from the images. For time course experiments quantifying temporal dynamics and diatom cell lysis, plates remained on the microscope stage, with an LED panel constructed to provide a 12:12 day:night cycle (P3030W, LED express). Images were taken every 20 min for 72 h (10× Objective, Nikon ECLIPSE Ti Microscope equipped with Hamamatsu ORCAFlash4.0 digital camera). The resulting videos were analysed by eye, categorising the cells in each frame as cylindrical, spherical or lysed.

### Incubation experiments

To culture cells in a shared medium but in the absence of physical contact, sterivex filter units with 0.45 μm pore membranes were used (Merck, SVHVL10RC); *T. rotula* only cultures were injected into one side of the filter unit, and *A. macleodii*–*T. rotula* co-cultures into the other, which were then maintained at 14 °C with 12:12 day:night cycle. Positive control filter units were set up with co-cultures in both compartments and a negative control with *T. rotula* only cultures in both compartments. After 72 h, an aliquot from each compartment was placed on a glass slide and viewed under the microscope to quantify the cell phenotypes (10× Objective, Nikon ECLIPSE Ti Microscope equipped with Hamamatsu ORCAFlash4.0 digital camera).

### Scanning electron microscopy

For scanning electron microscopy (SEM), co-cultures were set up between axenic *T. rotula* and *A. macleodii* following the standard protocol and samples were fixed with EM-grade glutaraldehyde at a final concentration of 2.5% at 0, 5, 24 and 48 h after addition of the bacteria. Fixed cells were attached to a silicon support coated with poly-L-lysine. The samples were then washed sequentially for 5 min with 5 mL 2.5% glutaraldehyde, 5 mL seawater, 5 mL 1% osmium tetroxide, and then again with 5 mL seawater. The samples were dehydrated with an ethanol series in distilled water, with 5 min at each concentration (30%, 50%, 70%, 90% and 100%) followed by three 5 min washes with water-free 100% ethanol. The material was critical point dried (Autosamdri-931, Tousimis) and the supports were mounted on aluminium stubs using silver paint. The sample was then uniformly metalized with 4 nm Pt-Pd by cathode sputter-coating (CCU-010 HV, Safematic). Cells were imaged using an SU5000 scanning electron microscope (HITACHI, EM Wizard).

### Antibiotics

To determine the timepoint at which *T. rotula* becomes committed to the morphological change during co-culture with *A. macleodii*, antibiotics were added at incremental delays to inhibit *A. macleodii*. Final concentrations, confirmed to inhibit *A. macleodii* growth, were 50 μg mL⁻¹ kanamycin sulfate and 25 μg mL⁻¹ chloramphenicol. 100 μg mL⁻¹ ampicillin, which is non-inhibitory to *A. macleodii*, was included as a negative control (all Sigma-Aldrich). Antibiotics were added 0, 3, 6, 9, 24, or 48 h after co-culture initiation under standard conditions and the proportion of *T. rotula* protoplasts was quantified after 72 h.

### Preparation of phytoplankton exudates and solid-phase extraction

200 mL of xenic *T. rotula* CCMP 3096 was grown as described above to a density of 1 × 10^4^ cells/mL, then centrifuged at 1000 × *g* for 10 min. The supernatant was discarded, the pellet resuspended in artificial seawater to replace the L1 medium, and the culture incubated for a further 24 h to allow exudation. The culture was then gently filtered through 0.22 µm polyethersulfone membranes to remove cells and obtain cell-free exudates. Dissolved metabolites in the filtrate were concentrated by solid-phase extraction (SPE) using an HLB Oasis cartridge (6 cc, 200 mg; Waters). The cartridge was preconditioned with one column volume of methanol followed by one column volume of ultrapure water. After sample loading, the cartridge was rinsed with one column volume of ultrapure water to remove salts, dried and then eluted with 80% methanol. The eluate was dried under a gentle stream of nitrogen and resuspended in sterile ASW to 0.5 mg/mL prior to use in chemotaxis assays.

### In situ chemotaxis assay (ISCA)

The chemotactic response of *A. macleodii* to *T. rotula* exudates was quantified using an in situ chemotaxis assay (ISCA)^116^. ISCA wells were filled with either SPE-concentrated exudates or artificial seawater as a control, and devices were incubated for 1 h in bacterial culture at a concentration of 10^6^ cells/mL in artificial seawater. Following incubation, well contents were collected and bacterial abundance quantified by flow cytometry as described above. Three independent ISCAs were run simultaneously to act as biological replicates. The chemotactic index was calculated as the concentration of cells in each well normalised to the mean concentration in ASW control wells. Differences in chemotactic index between treatments and the ASW control were assessed by one-way ANOVA of log-transformed values followed by Dunnett’s post-hoc test; significance was defined as *P* < 0.05.

### Environmental co-occurrence and co-abundance analysis

The scripts used for environmental co-occurrence analysis are available at https://doi.org/10.5281/zenodo.22303717. Global contextualisation was conducted using the Microbe Atlas Project (MAP)^51^, the largest curated reference database of rRNA genes from microbiome and metabarcoding studies (https://microbeatlas.org/). The 16S rRNA sequence of *A. macleodii* strain ATCC 27126 (GenBank accession AY831617.1) and the 18S rRNA sequence of *T. rotula* (GenBank accession AF374480.2) sequences were queried using MAPseq^117^ (https://microbeatlas.org/mapseq), retrieving hits at 97% identity; no matches exceeding 97% identity were detected. SILVA taxonomy assigned these hits as *Alteromonas macleodii* (MAPv3;97_285) and Eukaryota: Diatomea/Mediophyceae (MAPv3;97_93659), respectively. These taxa were detected in 25,792 samples for *Alteromonas* and 23,099 samples for *Thalassiosira*, resulting in a dataset comprised of 990 samples in which both taxa were present. These samples were matched to geographic coordinates (from https://microbeatlas.org/downloads/) to plot the global co-occurrence (Supplementary Fig. 2a). Co-occurrence was assessed using a one-sided Fisher’s exact test against the total pool of 2,056,412 MAP samples.

For quantitative co-abundance analysis, samples were retained only if both *Alteromonas* and *Thalassiosira* had a minimum of 2 raw reads, to reduce the influence of low-count artefacts on relative abundance estimates, yielding a final dataset of 252 samples. Abundance values (as parts per million, ppm) were log_10_-transformed prior to statistical analysis. Associations between taxa were assessed using Pearson (*r*) and Spearman rank (*ρ*) correlations (Supplementary Fig. 2b). To assess the statistical significance of the observed correlation and contextualise it against a broader biological background, null distributions of Pearson and Spearman correlation coefficients were generated by correlating *Thalassiosira* abundance against all other bacterial taxa (Supplementary Fig. 2c), and *Alteromonas* abundance against all other eukaryotic taxa (Supplementary Fig. 2d), within the same co-occurring samples. These background correlations were derived from raw OTU abundance counts, independently processed to ppm values following the procedure described above. An empirical *P*-value was calculated as the proportion of background correlations meeting or exceeding the observed focal correlation.

### Dual transcriptomics: co-culture and RNA extraction

For total RNA extraction, *T. rotula* cultures in exponential growth phase (10 biological replicates, initiated from independently grown stock cultures; 560 mL at 1 × 10⁴ cells/mL in 2 L Erlenmeyer flasks) were maintained at 14 °C under a 12:12 h light:dark cycle. Five treatment cultures were inoculated with 5.6 mL *A. macleodii* (12 h culture in 2216 MB; final 1% v/v), and five controls received an equal volume of sterile 2216 MB. Samples were taken from each of the 10 cultures at 0, 5, 10, 24, 26, 30, 48, and 72 h post-inoculation. At each timepoint, 500 μL was transferred to a 24-well glass-bottom plate for quantifying diatom phenotypes, and two 35 mL aliquots were harvested for RNA extraction by centrifugation (2000 *g*, 3 min; Eppendorf 5804 R, A-4-44 rotor). The pellets were resuspended in 500 μL ROTI Aqua-Phenol (Carl Roth), transferred to tubes containing glass beads (BashingBeads, Zymo Research), shaken (3 min, vortex setting 10; Vortex-Genie 2 with Multi Tube Holder, LabGene Scientific), incubated at room temperature for 2 min, then centrifuged at 12,000 *g* for 7 min (Eppendorf 5425 R, FA-24 × 2 rotor). The supernatants were pooled and stored at −80 °C until processing of all timepoints.

### Clean up, DNase, RT and qPCR

The RNA samples were cleaned and concentrated using an RNeasy Minikit (QIAGEN), following the RNA clean-up protocol, and eluting in 46 μL RNase-free water (kit-supplied). RNA concentration and purity were measured on a NanoDrop spectrophotometer (NanoDrop OneC, Thermo Fisher Scientific). All samples with an A260/A230 ratio below 1.80, indicating phenol contamination, were subjected to standard ethanol precipitation: 0.1 volumes 3 M sodium acetate (pH 5.2) and 3 volumes ice-cold 100% ethanol were added, samples were vortexed, and RNA was precipitated overnight at −80 °C. Thawed samples were centrifuged at 15,900 *g* for 30 min at 4 °C (Eppendorf 5425R centrifuge, FA-24 × 2 rotor). The pellets were washed twice with 0.5 mL ice-cold 75% ethanol (centrifuging at 4 °C for 10 min each time), air-dried, and then resuspended in 46 μL nuclease-free water. RNA concentration and purity were measured again on a NanoDrop OneC.

DNA digestion was performed with TURBO DNase (Thermo Fisher Scientific) in 50 μL reactions containing 5 μL 10× reaction buffer, 1 μL TURBO DNase, and 44 μL RNA solution, incubated at 37 °C for 30 min. Digestion was terminated with 5 μL DNase inactivation reagent, incubated at room temperature for 5 min, and centrifuged at 10,000 × *g* for 1.5 min. The supernatant was transferred to a fresh tube, and RNA concentration was measured again using both NanoDrop and the Qubit Broad-Range RNA Assay Kit (Thermo Fisher Scientific) in a 384-well plate format.

In order to confirm gDNA removal and the presence of RNA, reverse transcription was performed in 20 μL reactions containing 15 μL nuclease-free water, 1 μL of 1:10 diluted RNA, and 4 μL 5X Reaction Mix (iScript RT supermix, Bio-Rad). For each sample, both RT and no-RT controls were prepared. Amplification of *T. rotula* and *A. macleodii* DNA was detected using the 7500 FAST Real-Time PCR System (Thermo Fisher Scientific) in reactions containing 4 μL 5X HOT FIREPol EvaGreen qPCR Mix Plus (Solis BioDyne), 250 nM each of *T. rotula* Rpl13 primers (Rpl13-F ACTTCGCCTCAAGCCAAAC, Rpl13-R TTGACCTCAGCCAATTCTCC, designed for this study using NCBI Primer-BLAST and synthesised by Integrated DNA technologies (IDT)) or *A. macleodii* 16S primers (16S-27F AGAGTTTGATCMTGGCTCAG, 16S-1492R GGTTACCTTGTTACGACTT, universal 16S primers), and 1 μL template solution. gDNA removal was verified by checking for the absence of amplification in no-RT reactions; RNA presence was confirmed by checking for amplification in RT reactions. Negative template controls were included for each primer pair. All reactions were conducted in duplicate. The PCR amplification programme consisted of an initial denaturation step at 95 °C for 12 min, followed by 40 cycles of 95 °C for 15 s, 62 °C for 30 s, and 72 °C for 60 s. Melt curves were checked for each sample to confirm amplification of the correct product.

### rRNA depletion and library preparation

rRNA depletion and dual-library preparation were performed using the Zymo-Seq RiboFree Total RNA Library Kit (R3003, Zymo Research), a probe-free method that generates combined libraries from both organisms. For each sample, 500 ng RNA from the post-DNase step was used as input. Dual-library concentration was measured with the Qubit dsDNA HS Assay Kit (Thermo Fisher Scientific), and size distribution and quality were assessed using the High Sensitivity D1000 ScreenTape assay on a TapeStation system (Agilent). An equimolar pool of all libraries was prepared and re-analysed using Qubit and TapeStation assays.

### Sequencing

The dual RNA-seq libraries were sequenced as 150 bp paired-end reads using the NovaSeq Reagent Kit v1.0 (300 cycles) on a NovaSeq 6000 System (Illumina) at the Genetic Diversity Centre, ETH Zurich. Preliminary experiments confirmed that the ratio of reads mapping to *T. rotula* and *A. macleodii* was favourable, providing sufficient coverage of both organisms without requiring deeper sequencing.

### RNA sequence processing

The scripts used for transcriptome processing in this study are available at https://doi.org/10.5281/zenodo.21890585. Sequence reads were processed to remove Illumina-specific adaptors and were quality-trimmed using BBduk (BBtools v37.61; sourceforge.net/projects/bbmap) with default parameters and a minimum length of 100 nucleotides. Error correction of the reads was performed using the Bayes-Hammer module of the SPAdes assembler^118^ (v3.15.4). Only paired reads were retained, and only those pairs where both reads mapped in the correct orientation and with the correct insert size. To remove ribosomal RNA-like sequences from the dataset, high-quality reads were mapped sequentially to the SILVA SSU and LSU databases^119^ (v138.1) and to the Y2K 5S rRNA database^120^, using BBmap v37.36 (sourceforge.net/projects/bbmap). Reads with a sequence identity threshold of >70% were removed.

To obtain an overview of the microbial community composition in the xenic *T. rotula* cultures (without co-cultivation with *A. macleodii*), reads that had been mapped to small subunit (SSU) rRNA during the rRNA-filtering step were mapped to the SILVA non-redundant SSU Ref database (v.138.1) and assigned to an approximate taxonomic affiliation (nearest taxonomic unit, NTU) using PhyloFlash v3.0^121^ (http://github.com/HRGV/phyloFlash). The results provided a qualitative estimate of the community composition, as residual rRNA reads were obtained from libraries subjected to rRNA depletion. *Alteromonas macleodii*–assigned NTUs were detected (representing ~3.1% of the residual rRNA reads across four replicates), confirming its presence in the xenic *T. rotula* CCMP 3096 cultures. Results are summarised in Supplementary Data 1.

### *T. rotula* de novo transcriptome assembly

Due to the lack of a reference genome for *T. rotula* at the time of analysis, de novo assembly of the diatom transcriptome from the dual RNA-seq data was performed. To facilitate this, mRNA reads resembling *A. macleodii* were removed by mapping to the *A. macleodii* ATCC 27126 genome (NCBI:txid529120) at low stringency (>70% sequence identity) using BBMap. The remaining reads were normalised in silico to a target average depth of 80× using BBnorm (BBTools v37.61). A de novo transcriptome co-assembly was generated from the normalised, concatenated reads of all samples using rnaSPAdes^122^ (SPAdes v3.15.4) with default parameters (K-mer sizes: 49, 73). Non-diatom contaminants were removed by DIAMOND BLASTx (v2.0.13) searches against the NCBI non-redundant protein database (nr; July 2022), discarding sequences with significant hits (*e*-value  < = 10^−15^) to Bacteria, Archaea, or Metazoa. To reduce transcriptome redundancy, assembled transcripts were clustered using CD-HIT-EST^123^ (v4.8.1) with a sequence identity threshold of 95% and global alignment (-c 0.95 -g 1). The decontaminated transcriptome assembly comprised 76,185 contigs (total length 85.4 Mb; N50 = 2,231 bp; average contig length = 1122 bp). CDS retained after filtering had a mean length of 1.3 kb and N50 of 1.8 kb (*n* = 35,514).

Quality and completeness of the de novo transcriptome assembly were evaluated using Benchmarking Universal Single-Copy Orthologs (BUSCO)^124^ v5.8.3 with the Stramenopiles lineage dataset, yielding 97% complete BUSCOs (59% single-copy, 38% duplicated), 2% fragmented, and 1% missing out of 100 orthologs. To further evaluate concordance with genomic resources, transcript-derived coding DNA sequences (CDS; see below) were aligned to the recently published *T. rotula* genome (strain FE80)^125^, and reciprocal best hits were identified. At permissive thresholds (≥50% identity and ≥50% coverage), 95% of transcriptome-derived CDS and 80% of genome CDS showed a best hit to the other assembly, while under more stringent criteria (≥90% identity, ≥90% coverage), 66% of transcript-derived CDS still aligned to genome CDS. Reciprocal best-hit analysis identified ~47% of transcripts as having a one-to-one orthologous counterpart in the genome, and ~32–43% when identity and coverage filters were applied. A summary of assembly statistics, BUSCO scores, and genome alignment metrics is provided in Supplementary Data 3.

### Gene annotation

Transdecoder (https://github.com/TransDecoder/TransDecoder/) v.5.7.1 was used to predict candidate coding regions (CDSs), retaining only the single best open reading frame (ORF) per transcript. To reduce false-positive ORF predictions, we applied the --retain_blastp_hits and --retain_pfam_hits options during TransDecoder. Predict, using homology searches against the NCBI nr protein database (DIAMOND BLASTp, July 2022) and conserved domain searches against Pfam (HMMER hmmsearch, v3.3.2). Transcript sequences were translated into protein sequences, using a minimum ORF of 100 amino acids (TransDecoder.LongOrfs). Functional annotation of the translated protein sequences was performed with DIAMOND BLASTp against the NCBI and UniProt nr databases (both accessed July 2022), and domain prediction using InterProScan (v5.53-87.0)^126^ against the InterPro families database (v89.0)^127^. Additionally, orthology prediction was performed with eggNOG-mapper (v2.1.6)^128^ against the EggNOG database (v5.0). Assigned annotations are documented in Supplementary Data 5 for *T. rotula* and Supplementary Data 7 for *A. macleodii*.

These complementary approaches were deemed sufficient for all *A. macleodii* genes, and for *T. rotula* genes, which belonged to well-conserved families with clear orthologues. For *T. rotula* genes belonging to expanded families with functionally distinct paralogs, or where no gene of interest was directly annotated, targeted BLAST searches were performed against characterised homologues from reference organisms, to confirm or refine functional assignments. Putative homologues were defined as hits satisfying an *e*-value ≤ 1e − 6, an alignment coverage ≥50% of query length, and an amino acid identity of ≥40%. The source of each annotation assignment is documented in Supplementary Data 4; full BLAST outputs, including all hits and designated candidate homologues for downstream analyses, are provided in Supplementary Data 6.

### Quantification of transcript expression and differential expression analysis

Quantification of gene expression was performed by mapping the (non-normalised) mRNA reads to either the de novo assembled contigs of *T. rotula* or to the reference genome of *A. macleodii* (GenBank: CP003841.1) using Bbmap with a mapping identity threshold of 97% (minid=0.97). Alignments below this threshold were filtered out (idfilter = 0.97). Mapped reads were assigned to predicted ORFs in *T. rotula* and to the existing, annotated coding sequences of the reference *A. macleodii* genome using featureCounts^129^ (Subread package, v2.0.3), generating gene-level count tables that were used for downstream processing in R v 4.3.3. On average, 60.85% (range 27.29–87.34%) of all mRNA reads mapped to the *T. rotula* CDS dataset across all samples. In co-culture samples, an average of 8.62% (range 2.85–37.7%) of reads mapped to the *A. macleodii* CDS. Transcript representation and coverage for both the de novo transcriptome assembly and the reference *A. macleodii* genome are summarised in Supplementary Data 3.

Differential expression analyses were performed separately on the mapped read counts for *A. macleodii* and *T. rotula* using DeSeq2^130^ (v1.42.1). Only genes with a read count of 10 or higher in at least three samples were included in the analysis. Hierarchical clustering of the samples was performed using DESeq2-normalised data (subjected to Variance Stabilizing Transformation, VST) using Euclidean distance as the similarity metric. Differential expression of *T. rotula* genes between control (xenic *T. rotula* cultures) and *T. rotula* with *A. macleodii* co-culture samples was assessed at each timepoint (5, 10, 24, 26, 30, 48, and 72 h) using a two-sided likelihood ratio test in DESeq2. A negative binomial generalised linear model (GLM) was fitted using the design formula “~treatment+time+treatment:time”, an approach that controls for variation among replicates at time 0 h and tests whether the temporal expression patterns differ between treatments. Differential expression in *A. macleodii* upon interaction was assessed exclusively in co-culture samples. Pairwise comparisons were made between each timepoint and time 0 h, using a simpler design formula, “~time” in DESeq2. For all analyses, statistical significance was determined using a Benjamini–Hochberg false discovery rate threshold of 5%.

### Gene selection

The genes and pathways displayed in Figs. 3, 4 represent the output of a structured manual curation approach, selected to provide a clear and representative overview of the transcriptional response rather than an exhaustive list; complete differential expression results are provided via Figshare (https://doi.org/10.6084/m9.figshare.32907734)^131^. Differential expression of pathways hypothesised a priori to be relevant to the phenotype were interrogated, including sexual reproduction in *T. rotula* and ankyrin repeat proteins in *A. macleodii*. Otherwise, gene selection was data-driven: the most strongly up- and downregulated genes were identified, and their annotations used to define the cellular processes of greatest functional relevance to the observed response. The differential expression of all annotated members of those pathways was systematically examined to provide a pathway-level interpretation. Where a *T. rotula* gene included in Fig. 3 belongs to an expanded family in which co-annotated paralogues could not be confidently distinguished, Supplementary Data 4 now explicitly documents whether the selected gene is “Representative of family-wide expression pattern”, or a “Selected example from heterogeneously expressed gene family”. For expanded families identified through BLAST searches, the expression profiles of all homologous family members are provided in Supplementary Data 6.

### Statistics and reproducibility

No statistical method was used to predetermine sample size, consistent with standard practice sample sizes of *n *≥ 3 independent biological replicates were used throughout. For the dual transcriptome experiment, *n* = 5 biological replicates per condition were used, with each diatom culture initiated from independently grown stock cultures and each co-culture established independently. For microscopy experiments, *n* = 3 biological replicates were used, consisting of independent co-cultures established in separate wells with separate bacterial inoculations, each representing a distinct diatom-bacterium interaction. No data were excluded from the analyses. The experiments were not randomised; experimental groups were defined by the experimental treatment rather than by allocation of samples between groups, making randomisation not applicable. The investigators were not blinded to allocation during experiments and outcome assessment. Statistical analyses were performed in R (v4.3.3), Python (v3.11.5) and GraphPad Prism (v10.1.2.324); the specific statistical test used for data analysis and where appropriate, whether it was one-sided or two-sided and whether adjustments were made for multiple comparisons, is indicated in the relevant text and figure legends.

### Reporting summary

Further information on research design is available in the Nature Portfolio Reporting Summary linked to this article.

## Supplementary information

Supplementary Information

Description of Additional Supplementary Files

Supplementary Data 1

Supplementary Data 2

Supplementary Data 3

Supplementary Data 4

Supplementary Data 5

Supplementary Data 6

Supplementary Data 7

Supplementary Data 8

Supplementary Data 9

Supplementary Video 1

Supplementary Video 2

Supplementary Video 3

Reporting Summary

Transparent Peer Review file

## Source data

Source Data file

## Data Availability

Raw mRNA sequencing reads and the *Thalassiosira rotula* de novo transcriptome assembly used as a reference for transcript mapping are available in the European Nucleotide Archive under project accession PRJEB72406. The *Alteromonas macleodii* ATCC 27126 complete genome used for bacterial transcript mapping is publicly available under GenBank accession CP003841.1 (https://www.ncbi.nlm.nih.gov/nuccore/CP003841.1). Complete differential expression results and transcriptome annotation are deposited on Figshare (https://doi.org/10.6084/m9.figshare.32907734)^131^. Environmental co-occurrence data are publicly available through the Microbe Atlas Project (https://microbeatlas.org). All other data supporting the findings of this study are available within the paper and its Supplementary Information. Source data are provided with this paper.

## Code availability

Code for transcriptome processing and differential expression analysis is available at https://doi.org/10.5281/zenodo.21890585^132^. Code for environmental co-occurrence analysis is available at https://doi.org/10.5281/zenodo.22303717^133^.
