# Supplementary material for: Alteromonas macleodii breaches the diatom frustule triggering protoplast emergence and programmed necrosis-like death in Thalassiosira rotula
Source: Nat Commun. 2026 Sep 26;17:11158. doi: 10.1038/s41467-026-78079-7 (PMC13710752; doi:10.1038/s41467-026-78079-7)
Supplement: Supplementary file 1 [file 41467_2026_78079_MOESM15_ESM.pdf]

nature portfolio

Corresponding author(s): Isobel Short

Last updated by author(s): Aug 12, 2026

Reporting Summary

Nature Portfolio wishes to improve the reproducibility of the work that we publish. This form provides structure for consistency and transparency in reporting. For further information on Nature Portfolio policies, see our Editorial Policies and the Editorial Policy Checklist.

Statistics

For all statistical analyses, confirm that the following items are present in the figure legend, table legend, main text, or Methods section.

n/a	Confirmed	
☐	☒	The exact sample size (*n*) for each experimental group/condition, given as a discrete number and unit of measurement
☐	☒	A statement on whether measurements were taken from distinct samples or whether the same sample was measured repeatedly
☐	☒	The statistical test(s) used AND whether they are one- or two-sided<br>*Only common tests should be described solely by name; describe more complex techniques in the Methods section.*
☒	☐	A description of all covariates tested
☐	☒	A description of any assumptions or corrections, such as tests of normality and adjustment for multiple comparisons
☐	☒	A full description of the statistical parameters including central tendency (e.g. means) or other basic estimates (e.g. regression coefficient) AND variation (e.g. standard deviation) or associated estimates of uncertainty (e.g. confidence intervals)
☐	☒	For null hypothesis testing, the test statistic (e.g. *F*, *t*, *r*) with confidence intervals, effect sizes, degrees of freedom and *P* value noted<br>*Give P values as exact values whenever suitable.*
☒	☐	For Bayesian analysis, information on the choice of priors and Markov chain Monte Carlo settings
☐	☒	For hierarchical and complex designs, identification of the appropriate level for tests and full reporting of outcomes
☐	☒	Estimates of effect sizes (e.g. Cohen's *d*, Pearson's *r*), indicating how they were calculated

Our web collection on statistics for biologists contains articles on many of the points above.

Software and code

Policy information about availability of computer code

Data collection	Images were acquired using the manufacturer's proprietary software for each instrument, no custom data collection software was used: Fluorescence and time-lapse microscopy images were acquired using NIS-Elements (v5.02, Nikon) and scanning electron microscopy images were acquired using EM Wizard (Hitachi, SU5000 version). Flow cytometry data were collected using CytExpert (v2.6, Beckman Coulter).
Data analysis	Transcriptome processing, assembly, and differential expression analyses were performed using publicly available tools including BBTools (v37.61), BBMap (v37.36), SPAdes (v3.15.4), DIAMOND (v2.0.13), TransDecoder (v5.7.1), InterProScan (v5.53-87.0), eggNOG-mapper (v2.1.6), featureCounts (v2.0.3), PhyloFlash (v3.0), BUSCO (v5.8.3), CD-HIT-EST (v4.8.1), HMMER hmmsearch (v3.3.2), DESeq2 (v1.42.1) in R (v4.3.3). The code used for transcriptome processing and differential expression analysis is available at https://doi.org/10.5281/zenodo.21890585. Environmental co-occurrence analysis was performed using the Microbe Atlas Project database (https://microbeatlas.org/), with analysis and figure generation performed in Python (v3.11.5). The code for environmental co-occurrence analysis is available at https://doi.org/10.5281/zenodo.21886045. GraphPad Prism (v10.1.2.324) was used for statistical analyses and figure generation, Adobe Illustrator (v27.8.1) was used for figure preparation and Inkscape (v1.1.4) was used for preparing Figure 5.

For manuscripts utilizing custom algorithms or software that are central to the research but not yet described in published literature, software must be made available to editors and reviewers. We strongly encourage code deposition in a community repository (e.g. GitHub). See the Nature Portfolio guidelines for submitting code & software for further information.

Data

Policy information about availability of data

All manuscripts must include a data availability statement. This statement should provide the following information, where applicable:

- Accession codes, unique identifiers, or web links for publicly available datasets
- A description of any restrictions on data availability
- For clinical datasets or third party data, please ensure that the statement adheres to our policy

Raw mRNA sequencing reads and the Thalassiosira rotula de novo transcriptome assembly used as a reference for transcript mapping are available in the European Nucleotide Archive under project accession PRJEB72406 (https://www.ebi.ac.uk/ena/browser/view/PRJEB72406). The Alteromonas macleodii ATCC 27126 complete genome used for bacterial transcript mapping is publicly available under GenBank accession CP003841.1 (https://www.ncbi.nlm.nih.gov/nuccore/CP003841.1). Complete differential expression results and transcriptome annotation are deposited on Figshare (https://doi.org/10.6084/m9.figshare.32907734)131. Environmental co-occurrence data are publicly available through the Microbe Atlas Project (https://microbeatlas.org). All other data supporting the findings of this study are available within the paper and its Supplementary Information. Source data are provided with this paper.

Research involving human participants, their data, or biological material

Policy information about studies with human participants or human data. See also policy information about sex, gender (identity/presentation), and sexual orientation and race, ethnicity and racism.

Reporting on sex and gender	*Use the terms sex (biological attribute) and gender (shaped by social and cultural circumstances) carefully in order to avoid confusing both terms. Indicate if findings apply to only one sex or gender; describe whether sex and gender were considered in study design; whether sex and/or gender was determined based on self-reporting or assigned and methods used.<br>Provide in the source data disaggregated sex and gender data, where this information has been collected, and if consent has been obtained for sharing of individual-level data; provide overall numbers in this Reporting Summary. Please state if this information has not been collected.<br>Report sex- and gender-based analyses where performed, justify reasons for lack of sex- and gender-based analysis.*
Reporting on race, ethnicity, or other socially relevant groupings	*Please specify the socially constructed or socially relevant categorization variable(s) used in your manuscript and explain why they were used. Please note that such variables should not be used as proxies for other socially constructed/relevant variables (for example, race or ethnicity should not be used as a proxy for socioeconomic status).<br>Provide clear definitions of the relevant terms used, how they were provided (by the participants/respondents, the researchers, or third parties), and the method(s) used to classify people into the different categories (e.g. self-report, census or administrative data, social media data, etc.)<br>Please provide details about how you controlled for confounding variables in your analyses.*
Population characteristics	*Describe the covariate-relevant population characteristics of the human research participants (e.g. age, genotypic information, past and current diagnosis and treatment categories). If you filled out the behavioural & social sciences study design questions and have nothing to add here, write "See above."*
Recruitment	*Describe how participants were recruited. Outline any potential self-selection bias or other biases that may be present and how these are likely to impact results.*
Ethics oversight	*Identify the organization(s) that approved the study protocol.*

Note that full information on the approval of the study protocol must also be provided in the manuscript.

Field-specific reporting

Please select the one below that is the best fit for your research. If you are not sure, read the appropriate sections before making your selection.

☒ Life sciences ☐ Behavioural & social sciences ☐ Ecological, evolutionary & environmental sciences

For a reference copy of the document with all sections, see nature.com/documents/nr-reporting-summary-flat.pdf

Life sciences study design

All studies must disclose on these points even when the disclosure is negative.

Sample size	No statistical method was used to predetermine sample size: Consistent with standard practice in microbiology, sample sizes of n ≥ 3 independent biological replicates were used throughout. For the dual transcriptome experiment, n = 5 biological replicates per condition were used, with each diatom culture initiated from independently grown stock cultures and each co-culture established independently. The sample size for the transcriptome was determined based on the maximum number of replicates feasible given the scale and cost of dual transcriptome experiments and is in agreement with the requirements of standard transcriptome protocols. For microscopy experiments, n = 3 biological replicates were used, consisting of independent co-cultures established in separate wells with separate bacterial inoculations, each representing a distinct diatom-bacterium interaction.
Data exclusions	No data were excluded from the analyses.

Replication	There were five biological replicates for each treatment (co-culture or negative control): all attempts at replication were successful, and no replicates were excluded from the analyses.
Randomization	Randomisation was not applicable to this study; experimental groups were defined by the experimental treatment (presence or absence of A. macleodii).
Blinding	The investigators were not blinded during group allocation, data collection or analysis for any experiments. Blinding was not pertinent to this study as it did not include any animal or human research participants, and was not possible as many analyses were carried out by the person responsible for sampling. Transcriptomic data analysis was performed computationally using predefined pipelines and statistical thresholds, which are not subject to observer bias.

Reporting for specific materials, systems and methods

We require information from authors about some types of materials, experimental systems and methods used in many studies. Here, indicate whether each material, system or method listed is relevant to your study. If you are not sure if a list item applies to your research, read the appropriate section before selecting a response.

Materials & experimental systems

n/a	Involved in the study
☒	☐ Antibodies
☐	☒ Eukaryotic cell lines
☒	☐ Palaeontology and archaeology
☒	☐ Animals and other organisms
☒	☐ Clinical data
☒	☐ Dual use research of concern
☒	☐ Plants

Methods

n/a	Involved in the study
☒	☐ ChIP-seq
☐	☒ Flow cytometry
☒	☐ MRI-based neuroimaging

Eukaryotic cell lines

Policy information about cell lines and Sex and Gender in Research

Cell line source(s)	T. rotula diatom cells were purchased from NCMA (see specific strain names in the study).
Authentication	The cell lines were not authenticated.
Mycoplasma contamination	The cells were not tested for mycoplasma contamination.
Commonly misidentified lines (See ICLAC register)	*Name any commonly misidentified cell lines used in the study and provide a rationale for their use.*

Plants

Seed stocks	*Report on the source of all seed stocks or other plant material used. If applicable, state the seed stock centre and catalogue number. If plant specimens were collected from the field, describe the collection location, date and sampling procedures.*
Novel plant genotypes	*Describe the methods by which all novel plant genotypes were produced. This includes those generated by transgenic approaches, gene editing, chemical/radiation-based mutagenesis and hybridization. For transgenic lines, describe the transformation method, the number of independent lines analyzed and the generation upon which experiments were performed. For gene-edited lines, describe the editor used, the endogenous sequence targeted for editing, the targeting guide RNA sequence (if applicable) and how the editor was applied.*
Authentication	*Describe any authentication procedures for each seed stock used or novel genotype generated. Describe any experiments used to assess the effect of a mutation and, where applicable, how potential secondary effects (e.g. second site T-DNA insertions, mosiacism, off-target gene editing) were examined.*

Flow Cytometry

Plots

Confirm that:

☒ The axis labels state the marker and fluorochrome used (e.g. CD4-FITC).

☒ The axis scales are clearly visible. Include numbers along axes only for bottom left plot of group (a 'group' is an analysis of identical markers).

☒ All plots are contour plots with outliers or pseudocolor plots.

☒ A numerical value for number of cells or percentage (with statistics) is provided.

Methodology

Sample preparation	Algal density was measured with no additional sample preparation. Bacterial density was measured following sample staining with SYBR Green I staining (ThermoFisher), for 15 minutes in the dark.
Instrument	cytoFLEX, Beckman Coulter
Software	CytExpert v2.6
Cell population abundance	Algal cells were identified by autofluorescence across three excitation channels, APC (638 nm), PE (585 nm), and FITC (525 nm), plotted against forward scatter (FSC). Background signal from L1 medium within these gates was confirmed as zero, and cell counts were averaged across the three channels to determine cells per mL. Bacterial cells were identified by FITC-A fluorescence plotted against FSC following staining. Cell sorting was not performed.
Gating strategy	Algal cell gates were defined based on diatom autofluorescence in APC, PE, and FITC channels plotted against FSC. Bacterial gates were defined based on FITC-A fluorescence plotted against FSC.

☒ Tick this box to confirm that a figure exemplifying the gating strategy is provided in the Supplementary Information.
