# Supplementary material for: Alteromonas macleodii breaches the diatom frustule triggering protoplast emergence and programmed necrosis-like death in Thalassiosira rotula
Source: Nat Commun. 2026 Sep 26;17:11158. doi: 10.1038/s41467-026-78079-7 (PMC13710752; doi:10.1038/s41467-026-78079-7)
Supplement: Supplementary file 2 [file 41467_2026_78079_MOESM16_ESM.pdf]

nature portfolio

Peer Review File

Alteromonas macleodii breaches the diatom frustule triggering protoplast emergence and programmed necrosis-like death in *Thalassiosira rotula*

Corresponding Author: Dr Uria Alcolombri

This file contains all reviewer reports in order by version, followed by all author rebuttals in order by version.

Version 0:

Reviewer comments:

Reviewer #1

(Remarks to the Author)
The study presents a holistic analysis, combining microscopy, cell counts, and transcriptomics over multiple experiments, to investigate the interaction between an algicidal Alteromonas macleodii strain and its diatom host. The manuscript is well written, though at times somewhat dense, as a result of the various datasets and experimental outcomes being synthesized. A central claim of the work is that protoplast emergence represents a novel algicidal mode of action. However, protoplast formation is a stress response in diatoms, and the data presented here do not fully disentangle this broader phenomenon from a specific bacterially induced mechanism. Notably, the addition of bacterial culture medium alone increases protoplast formation from approximately 1% to 10% in xenic Thalassiosira rotula cultures; and it is observed even in the absence of bacteria. Although this response is far less pronounced than the ~95% observed in the A. macleodii co-culture, it raises important questions about the extent to which protoplast emergence is specifically triggered by this strain versus reflecting a more general stress-induced process.
The study ultimately does not identify the underlying molecular mechanism driving the observed phenotype. While the focus on protoplast emergence is relevant & interesting, this response can occur under a variety of stressful conditions, and the current dataset does not establish how characteristic (unique) this phenomenon is for this bacterial strain/species. Only two additional bacterial strains are tested, limiting the strength of the conclusions regarding specificity.
To make this study truly impactful, fitting the scope of Nature Communications, I recommend one of two paths:
(1) expanding the panel of bacterial strains tested with T. rotula to more convincingly demonstrate that protoplast formation is a distinctive feature of A. macleodii–diatom interactions
OR

(2) providing deeper insight into the molecular mechanism underlying the response. I recognize that the latter may be challenging and dependent on experimental tractability, but either approach would substantially strengthen the manuscript's central claims.

Minor comments:
L109-110:'a high proportion' & 'in each case': can you be clearer, and ideally, more quantitative on this: so in all X replicates, y% of the cells ?

L145: why use these specific bacteria (Ruegeria pomeroyi DSS-3 and Vibrio alginolyticus) and why just these two strains/only this condition?
L189: 'control' for the bacteria, this is the bacteria dissolved in diatom culture medium?
L211 & elsewhere (like L314: biological replicates: where these independent cultures? If you used one stock culture, split into 2 x 5 cultures just prior to the experiment, it seems more accurate to refer to these as technical replicates
L212: why de novo (T. rotula genome available: https://www.nature.com/articles/s41597-025-04634-4)
Fig 3a: The transcriptome shows a clear diurinal pattern, this needs to be addressed or at least mentioned in the results
Fig 3a: consider using a clear gradient for the time points (either color of figure complexity)
Fig3b-4b: only a subset of genes is shown, it is not explained how this subset was chosen
L355: Whilst chemical inhibition of proteases could not be tested due to toxicity to T. rotula -> either clarify or remove statement

L545: How are lysed, protoplast, intact cell distinguished with flow cytometry? Gates set based on test with each group?

Reviewer #2

(Remarks to the Author)
The authors describe a fascinating interaction between the bacterium Alteromonas macleodii and the diatom Thalassiosira rotula. They use a comprehensive set of tools to infer that the attachment of the bacterium to the alga causes a sequence of events that leads to algal cell lysis. Compared to co-culture, enhanced DOC in xenic cultures can cause the same phenomenon (but to a far lesser extent). That A macleodii is not exclusively antagonistic but has repurposed its opportunistic copiotrophic traits to mediate host exploitation in this context is intriguing.

I congratulate the authors on such thorough research. From the text to the visual presentation of data, the standard is excellent.

There are a few areas however, that could be tightened.

On the language used to describe programmed cell death. Clearly reconciling "the activation of PCD markers did not fully align with canonical pathways" on line 259, with "this marks the first evidence of PCD in T rotula" (line 454) [later on line 457, a distinct PCD pathway] If key PCD genes showed weak or non-significant expression changes (line 264-266) and "the necrosis is independent of NO- and autophagy-independent form of PCD, divergent from canonical pathways", then I think consistency around necrotic-like or novel PCD pathway rather than simply PCD is advisable.

On describing the timing of Phases. The explanation on line 179 states T rotula's fate is determined within 9 h of exposure", and Phase 1 lasts 0-10 h, but then I see that sampling for transcriptomes was at 5 and 10 h. On line 407-9 T rotula cells first undergo growth arrest (0-10 h), rapid and irreversible protoplast formation (10-35) etc. Given samples were taken at 10 h, it would help if Phase labeling made it clear which Phase the 10 h sample was considered in, not leave it ambiguous.

On significance of the interaction. An area of general uncertainty that remains for me is how relevant this interaction is in the ocean. It is one thing for A. macleodii to cooccur with T. rotula in the volume of a Niskin bottle, but it is quite another for them to occur at the micrometer scale of the phycosphere.

Narrowing this uncertainty would involve such evidence as time-course quantification of A. macleodii during T. rotula blooms in the ocean or detecting A. macleodii in diatom cultures with protoplasts. In the absence of that, then the probability of such interactions in the ocean remains unknown and the language about the significance of this as an -/+ ecological interaction needs to be somewhat moderated – i.e., line 522 "the seismic impact bacterial interactions can exert on diatom ecology"

I highlight a few more points below.

Line 82: what is the natural abundance of these organisms in the ocean? Their ratio is mentioned but not absolute abundance

On species specificity Line 144: various other bacteria were tested against T rotula, and different diatoms against A macleodii, but what about different strains of T rotula against A macleodii? [I didn't think this cross-testing was extensive, so that's why I ask just exactly how specific it is.]

On replication Line 198: were the three independent replicates in the same multiwell plate? The imaging time alone would have made that problematic, but I ask because the standard deviation is small which suggests cells were coherent in their responses. Within 1 well, 100 cells needed to be found. To what extent was this randomized?

Line 279 – on first mention, elaborate on AIF apoptosis inducing factor

Line 407-409. The interaction is described from the algal perspective but it involves two players, so I would prefer to see this phase description that also describes what the bacteria are doing.

Is Figure 5 cited in the text?

Line 422: What is in the culture medium 2216 MB that overlaps with the exometabolome of T rotula?

Line 447: Is there a dilution of bacteria vs T rotula that was also tested as a threshold for protoplast formation?

From a technical standpoint, the 14 deg C temperature for diatom culture did not overlap well with the temperature used to grow bacteria. If 14 deg C was used as the incubation temperature then this must have slowed bacteria growth considerably. Did any technical complications arise?

Figures – Padj significance shown in Fig. 3 – the asterisks are too small. An asterisk shouldn't be used for $P<0.1$. It is non-significant and is misleading without an explanation of why.

While the quality of the plots is high, if it was me, I would be plotting the same time points in Fig 3a and Fig 4a with the same

colour codes so that there's consistency. I appreciate however that colour may have been different because of the algal vs bacteria content.

Fig. 5 is excellent – well done!

Please note the declaration indicated that Flow cytometry was n/a whereas it was used to enumerate T rotula

Reviewer #3

(Remarks to the Author)
In the manuscript by Short et al., authors examined the interaction between the diatom Thalassiosira rotula and the bacteria Alteromonas macleodii, naturally found as member of this diatom's microbiome. They discovered a novel algicidal action of A. macleodii through diatom protoplast emergence from the diatom silica shell, frequently followed by cell lysis. This interaction requires direct contact between diatom and bacteria cells and is species-specific, being variable also between different strains of A. macleodii and T. rotula and strongest between A. macleodii ATCC 27126 and T. rotula CCMP 3096. A dual transcriptomic analysis revealed that T. rotula gene expression switches from signaling and repair pathways activation in the first phase of diatom-bacteria contact to programmed cell death activation from phase 2 onwards. For A. macleodii, genes responsible for motility and chemotaxis were upregulated in all phases to colonize host cells, and gene expression reveals a sequential use of different diatoms derived compounds.
Overall, results suggest that this bacterium could induce and exploit an already observed stress response mechanism in diatoms, i.e. protoplast extrusion from the frustule, to benefit from diatom-derived organic compounds. This is first evidence of bacteria inducing this stress response, thus representing a novel mechanism of action that needs to be further explored to fully disclose the extent of bacterial role in protoplast formation and eventually algicidal effectors at the basis of this interaction. Thus, the manuscript content represents a useful refinement of current knowledge on diatom-bacteria algicidal interactions.
Conclusions drawn by the authors are overall well supported by the analysis performed and the methodology applied is appropriate and consistent with the literature. There are however several claims that would benefit from more in-depth analyses. This applies primarily to the transcriptomic analysis for the diatom counterpart, which in some cases appears superficial. Indeed, by randomly checking T. rotula differentially expressed genes, we found some inaccuracies in genes description in the text referring to Figure 3b and related Supplementary Tables S3 and S4. In many cases, the genes listed belong to gene families or have paralogs, and while it is not expected that each gene is manually checked, caution should be used when drawing conclusions. For instance, SPO11 can be SPO11-2, specific for meiosis, or SPO11-3, also used for mitosis, therefore it would be important to specify which one is the one in the figure, and how this was determined. Cyclins in diatoms are expanded (up to 52 members in T. pseudonana), the different family members can have different functions. Similarly, cathepsins are of many kinds, and the meaning of their modulation is described only in a general way. Moreover, % amino acid identity with sequences from other organisms are reported along the text but without information on the top hits or on the thresholds selected to assign identity- there is nothing in the methods- their utility for the comprehension of the data is not clear. Thus, a more accurate check of annotations and description of gene expression variation needs to be added in the results section.
Another claim which seems based on expectations rather than data is the claim that there is a growth arrest in the diatom at 10 h (stated on lines 164-165, and two more times in the manuscript). In Figure 2a, the progression of control and treatment seems very similar, then the treated sample experiences a collapse at 24 h due to bacterial lysis. There are no data flow cytometry on the cell cycle, nor a statistical analysis to show that the treated sample is different, therefore I would modify the text unless support can be provided.
Overall, the method section contains the main information necessary to reproduce the work performed, with the exceptions here reported:
Lines 143-147: We observed no morphological changes in T. rotula CCMP 3096 co-cultured with two other algal-associated bacteria, Ruegeria pomeroyi DSS-3 and Vibrio alginolyticus. Likewise, A. macleodii ATCC 27126 caused no morphological changes in the diatoms Thalassiosira weissflogii CCMP 1050, Thalassiosira pseudonana CCMP 3367 or Phaeodactylum tricornutum CCMP 2560.

There is information in the methods section regarding the cultivation of these diatom species but nothing about the other bacteria tested and no results are shown in the manuscript. Moreover, more details on the reason why these two bacteria have been chosen for this experiment can be added.

Line 303: ROS staining with CM-H2DCFDA (Methods) showed that after 10 h of co-culture,
No information is reported in the methods section. Moreover, Figure 3c results unclear since the symbols that should identify the different time points, or the independent replicates cited in the description, are not reported in the figure.

Other minor revisions are here reported:
Line 228: upregulation of signaling proteins such as G protein-coupled receptor, Gpcr, and kinase tor

Supplementary Table S3 and S4 show the annotation of these genes, where it is reported that Gpcr has a 7tm_3 domain, suggesting that this gene is a class 3 Gpcr. Here again, there are many Gpcrs in diatoms. Moreover, the annotation for the gene that the authors define as TOR kinase corresponds to Raptor, one of proteins involved in the TORC1 complex together with TOR. This supports an involvement of the TOR signalling pathway, it would be interesting to check if the TOR kinase is also regulated. In any case the text needs to be corrected.

Line 619: Antibiotics and Inhibitors

Which are the inhibitors used? The molecules listed in the paragraph are all antibiotics.

Line 654: Clean up, DNase, RT and qPCR
Are qPCR data presented in the manuscript?

Overall, the work is interesting and worthy of publication. The authors propose the necrotic-like cell death as a novel mechanism and this seems supported in large part by the cluster of genes in the Necrosis category in Fig. 3b, indeed upregulated, therefore it is fundamental to be accurate on the genes described providing a more solid account of the gene identity.

Reviewer #4

(Remarks to the Author)
I co-reviewed this manuscript with one of the reviewers who provided the listed reports. This is part of the Nature Communications initiative to facilitate training in peer review and to provide appropriate recognition for Early Career Researchers who co-review manuscripts.

Version 1:

Reviewer comments:

Reviewer #1

(Remarks to the Author)
I would like to thank the authors for the substantial effort they have invested in revising the manuscript. I appreciate the many improvements that have been made, including the additional co-culture experiments, which have strengthened the study.

I particularly appreciated the addition of the assessment of the environmental plausibility of the observed interaction, which was included in response to another reviewer's comments. This discussion provides valuable context and enhances the ecological relevance of the work.

Overall, the revisions,including the slight shift in focus, have, in my view, increased the broader relevance and impact of the findings. As a result, I believe this manuscript now represents a strong contribution that is well suited for publication in this journal.

Although one could still debate whether the terminology used to describe the replicates (i.e., biological versus technical replicates) is the most appropriate, I do not consider this a significant issue and do not request any further changes to the manuscript.

Reviewer #2

(Remarks to the Author)
The authors have taken the reviewers comments seriously and should be commended for the additional work they have done before resubmitting the manuscript. I find this work sound and well supported by the data.

I have only a few minor comments on the revised version.

Line 312 – Spell out “S.” marinoi on first use (see line 349)
Line 463 – remove full stop
Line 639 – What is the full magnification (this only looks like the eyepiece magnification)
Line 646 – The sentence needs a full stop.
Line 674 – Was any fixative used before SYBR Green staining?
Line 685 – “1x106 cells mL^{-1}L” remove L
Line 797 – was RNA from the two 35 mL replicates pooled?

Reviewer #3

(Remarks to the Author)
We thank the authors for their point-by-point responses to our queries and for carefully refining the manuscript, which we believe has strengthened the presented results. Overall, all our comments have been clearly addressed, and the necessary modifications have been implemented in the text. Therefore, we recommend the manuscript for publication in Nature Communications.
That said, we have a minor question out of curiosity regarding why results of the experiment examining morphological changes of other Thalassiosira species and of Phaeodactylum tricornutum when in co-culture with A. macleodii (lines 144-147) are only mentioned without accompanying figures or a 'data not shown' statement.
Another minor issue is that Extended data Fig. 2 is firstly cited before Extended data Fig. 1 (line 85 for the first and 166 for the second).

Reviewer #4

(Remarks to the Author)
I co-reviewed this manuscript with one of the reviewers who provided the listed reports. This is part of the Nature Communications initiative to facilitate training in peer review and to provide appropriate recognition for Early Career Researchers who co-review manuscripts.

Open Access This Peer Review File is licensed under a Creative Commons Attribution 4.0 International License, which permits use, sharing, adaptation, distribution and reproduction in any medium or format, as long as you give appropriate credit to the original author(s) and the source, provide a link to the Creative Commons license, and indicate if changes were made.
In cases where reviewers are anonymous, credit should be given to 'Anonymous Referee' and the source.
The images or other third party material in this Peer Review File are included in the article's Creative Commons license, unless indicated otherwise in a credit line to the material. If material is not included in the article's Creative Commons license and your intended use is not permitted by statutory regulation or exceeds the permitted use, you will need to obtain permission directly from the copyright holder.
To view a copy of this license, visit https://creativecommons.org/licenses/by/4.0/

Response to Reviewers

We thank the Reviewers for their careful reading of our manuscript and their constructive comments. We have addressed all points raised and provide a detailed point-by-point response below, with our responses in blue. Our updated manuscript with all changes tracked in blue is also provided.

Reviewer #1

The study presents a holistic analysis, combining microscopy, cell counts, and transcriptomics over multiple experiments, to investigate the interaction between an algicidal Alteromonas macleodii strain and its diatom host. The manuscript is well written, though at times somewhat dense, as a result of the various datasets and experimental outcomes being synthesized. A central claim of the work is that protoplast emergence represents a novel algicidal mode of action. However, protoplast formation is a stress response in diatoms, and the data presented here do not fully disentangle this broader phenomenon from a specific bacterially induced mechanism. Notably, the addition of bacterial culture medium alone increases protoplast formation from approximately 1% to 10% in xenic Thalassiosira rotula cultures; and it is observed even in the absence of bacteria. Although this response is far less pronounced than the ~95% observed in the A. macleodii co-culture, it raises important questions about the extent to which protoplast emergence is specifically triggered by this strain versus reflecting a more general stress-induced process.

The study ultimately does not identify the underlying molecular mechanism driving the observed phenotype. While the focus on protoplast emergence is relevant & interesting, this response can occur under a variety of stressful conditions, and the current dataset does not establish how characteristic (unique) this phenomenon is for this bacterial strain/species. Only two additional bacterial strains are tested, limiting the strength of the conclusions regarding specificity. To make this study truly impactful, fitting the scope of Nature Communications, I recommend one of two paths:
(1) expanding the panel of bacterial strains tested with T. rotula to more convincingly demonstrate that protoplast formation is a distinctive feature of A. macleodii–diatom interactions OR
(2) providing deeper insight into the molecular mechanism underlying the response. I recognize that the latter may be challenging and dependent on experimental tractability, but either approach would substantially strengthen the manuscript's central claims.

We thank the Reviewer for their positive assessment of our study and for recognizing the broad experimental scope of the work. We have now provided a detailed, point-by-point response to each of the concerns raised by the reviewer:

1. We agree with the Reviewer's suggestion to expand the panel of bacterial strains tested to assess how unique this interaction is, and acknowledge that our original conclusions regarding specificity were limited. In response to this important recommendation, we established 24

additional pairwise co-cultures between *T. rotula* CCMP 3096 and bacterial strains spanning 9 families across the Alpha- and Gammaproteobacteria, using identical conditions to the *A. macleodii* co-culture experiments and assessing protoplast formation after 72 h (full strain details and rationale for strain choice are provided in Supplementary Table S2, and explained in answer to Reviewer 1's comment below). Of the 24 bacterial strains tested, 14 induced a significant increase in protoplast formation after 72 hours relative to the 2216 MB control ($P < 0.001$, see Fig. 1), yet none exceeded half (< 42.7% ± 1.8%) of the protoplasts induced by *A. macleodii* ATCC 27126 (99.3% ± 0.6%). The remaining 10 strains induced no significant effect.

The data demonstrate that protoplast formation is not simply a nonspecific response to bacterial presence. Nonetheless, several phylogenetically diverse bacterial taxa can induce protoplast formation in *T. rotula* CCMP 3096 to some extent, with this ability not confined to a specific bacterial lineage. While not exhaustive, the set of isolates tested spans a broad taxonomic range and originates from diatom- and particle-associated environments, suggesting that this capacity may be relatively widespread among marine bacteria. This finding thereby increases the likelihood of such interactions occurring in natural marine environments, expanding the environmental relevance of this phenomenon. Importantly, *A. macleodii* induced a substantially stronger and more synchronized response than the other strains tested, supporting its use as a model organism for the mechanistic characterization of the phenomenon.

In addition, our previous experiments across three *T. rotula* strains (CCMP 3096, 3264, and 3362) and three other diatom species showed that protoplast formation is restricted to *T. rotula* amongst those tested, with the strongest response observed in strain CCMP 3096. Together, these results show that *T. rotula* CCMP 3096 is particularly sensitive to bacterial-induced protoplast formation, and *A. macleodii* ATCC 27126 serves as a potent trigger, supporting the *T. rotula* CCMP 3096 - *A. macleodii* ATCC 27126 pairing as a valuable model system for studying bacterially-induced protoplast formation and its biological consequences.

We incorporate these new data into the revised manuscript as Fig. 1e and in the corresponding text in the results section (from line 139).

We thank the Reviewer for this suggestion, which we believe has substantially strengthened the manuscript and helped us clarify the scope and generality of our conclusions.

2. We appreciate the thoughtful and constructive comment regarding the interpretation and distinction between protoplast emergence as a bacterially induced algicidal mechanism versus a more general stress response.

We agree with the Reviewer that protoplast formation in diatoms can occur as a general stress response (as shown in Extended Data Fig. 2 and stated on line 486), and we do not claim that the cellular program underlying this response is unique to *A. macleodii*. However, our expanded bacterial screen demonstrates that protoplast formation is not simply a nonspecific response to bacterial presence: of 24 phylogenetically diverse strains tested, 10 induced no significant response. Furthermore, no other strains approached the potency observed in the *A.*

macleodii–T. rotula co-culture (99.3% ± 0.6%), with the next most potent strain inducing protoplast formation in only 42.7% ± 1.8% of cells (Fig. 1e). This demonstrates that *A. macleodii* possesses specific traits that drive the exceptionally strong population-level response, representing a highly effective bacterial strategy for accessing diatom-derived carbon, with clear consequences for carbon flux in marine environments. Therefore, the *T. rotula* CCMP 3096 - *A. macleodii* ATCC 27126 pairing, and the co-culture conditions used here, serve as a tractable and biologically relevant model system to dissect the mechanisms and ecological significance of bacterially induced protoplast formation, while simultaneously advancing our understanding of the broader - and understudied - phenomenon of diatom protoplast formation. To make this point clear, we have now added a sentence in the abstract, line 34 where we write: "*Whilst protoplast formation, a known diatom stress response, was induced by diverse bacteria, population-wide collapse was uniquely achieved by A. macleodii, and exploited here as a model to characterise the phenomenon*." We have also expanded the relevant paragraph to explicitly discuss the similarities and differences between bacterially induced and stress-induced protoplast formation and its ecological significance, and to frame the *A. macleodii–T. rotula* pairing as a model system for studying this underexplored phenomenon. We refer the Reviewer to this paragraph in the discussion, lines 486-506.

We would like to emphasize that although protoplast formation itself is not a novel cellular response in diatoms and can be induced by other stressors, its induction by bacteria has not been systematically studied. We have been careful to make this distinction clear, and we have now added a sentence in line 83 to clarify the motivation for our study in the introduction, reading: "*This is exemplified by the formation of protoplasts, a phenotype observed at low prevalence in nutrient-limited cultures of many diatom species*[1]*(Extended Data Fig. 2a, b), but never directly characterised, nor systematically investigated during interactions with antagonistic bacteria.*"

Importantly, in a recent paper by Graff van Creveld et al. (ISME J, 2026)[1], we found independent observation of what appears to be bacterial-induced protoplast formation in the pelagic diatom *Thalassiosira oceanica* caused by *Ekhidna algicida*, a member of the phylum Bacteroidota isolated from the Pacific Ocean. The authors describe the disruption of the silica frustule, and their Supplementary figure S6 shows the induction of what appear to be protoplasts in *T. oceanica* and *T. diporocyclus*. The discovery of multiple algicidal *Ekhidna* strains across the Pacific Ocean, each effective against diverse diatom species, supports our hypothesis that bacterial-induced protoplast formation may represent a widespread strategy for diatom antagonism in marine environments. These independent findings strengthen the case for the environmental prevalence and significance of bacterially-induced protoplast formation studied here, and we have added a reference to this paper in line 499, which reads "*The broader prevalence of this phenomenon is further supported by recent evidence for protoplast formation in Thalassiosira oceanica by multiple Ekhidna algicida strains, isolated across the Pacific ocean (Graff van Creveld et al., ISME J, 2026; Supplementary Materials therein)*". Our study, therefore, provides a systematic examination of this phenomenon, combining morphological, physiological, and gene expression analyses to begin identifying candidate molecular components that may underpin protoplast formation, a widespread yet understudied process.

3. In response to the Reviewer's point that the addition of bacterial culture medium alone significantly increases the proportion of protoplasts in the *T. rotula* population to 10% in xenic cultures, we emphasize that bacterial medium alone did not induce a significant increase in protoplasts in axenic *T. rotula* cultures (stated in line 134, and shown in Fig. 1a). In contrast, the response observed in xenic cultures suggests that the added DOC influences bacteria associated with the host, which subsequently induce the observed phenotype. These bacteria include *A. macleodii sp.*, which was detected by 16S rRNA analysis in the xenic cultures of *T. rotula* (see Supplementary Table S1). This difference between axenic and xenic cultures thus supports the interpretation that protoplast formation in the xenic culture in response to the addition of bacterial culture medium alone is still bacterially mediated, rather than reflecting a direct effect of the medium on the diatom itself. This interpretation is now stated clearly in the discussion, on line 523, "*The significant increase in protoplasts observed in xenic, but not axenic cultures suggests the phenotype results from DOC-mediated stimulation of resident bacteria, including A. macleodii, which was detected in our xenic diatom cultures by 16S rRNA analysis (Supplementary Table S1)*".

4. Regarding the missing insight into the molecular mechanism underlying the response, we fully agree with the Reviewer that exploring the precise molecular mechanism underlying the observed phenotype would strengthen the impact of the study. However, we respectfully suggest that elucidating a comprehensive mechanism is beyond the scope of the current manuscript, which already integrates a significant set of experimental approaches and data, as also recognized by the Reviewer.

We furthermore would like to note that the manuscript does provide some initial mechanistic insights. We present a working hypothesis behind the cell biology involved in protoplast formation in *T. rotula* and its relationship to necrotic-like programmed cell death. From the perspective of *A. macleodii,* we also describe several aspects of its virulence mechanism, including the use of chemotaxis, requirement for direct attachment, identification of a number of candidate effector genes and apparent lack of involvement of several canonical algicidal pathways, thereby narrowing the range of plausible mechanisms. We direct the Reviewer to lines 384-413 in the Results section and the re-written discussion paragraph on "*Mechanistic insights into A. macleodii's algicidal lifestyle*". We hope that this provides a useful foundation for future mechanistic studies while allowing the present manuscript to remain focused and coherent.

References:

1. Graff van Creveld, S. et al. Induced pathogenicity toward open-ocean diatoms by a filterable bacterium *Ekhidna algicida* sp. nov. *ISME J*. **20**, wrag038 (2026).

Minor comments:
L109-110:'a high proportion' & 'in each case': can you be clearer, and ideally, more quantitative on this: so in all X replicates, y% of the cells ?

To be more quantitative, we have removed the ambiguous qualifier “a high proportion” and the full sentence now reads “*Within 72 h of co-culture, 99.3% ± 0.6% of the diatom cells had undergone a striking morphological change, significantly more than the 3% ± 0.3% in control cultures without A. macleodii (mean ± SD, n = 3; one-way ANOVA with Tukey’s post-hoc test, P < 0.001; Fig. 1a)*” to line (118).

L145: why use these specific bacteria (Ruegeria pomeroyi DSS-3 and Vibrio alginolyticus) and why just these two strains/only this condition?
Ruegeria pomeroyi DSS-3 and *Vibrio alginolyticus* were initially selected due to their known interactions with algae; however, to address the limitation of the initial screen, we have now expanded the analysis to 25 strains in total, spanning 12 genera across the Alpha- and Gammaproteobacteria (new Fig. 1e). This screen allowed us to have a more comprehensive assessment of the specificity of this interaction and the results and implications of this expanded screen are outlined in the response to the previous point.

Regarding the new choice of bacteria: additional bacterial strains were selected to achieve broad phylogenetic coverage whilst prioritising taxa associated with marine phytoplankton and particle-associated environments, including both isolates obtained from xenic diatom cultures and strains with previously described interactions with marine phytoplankton.

- We first tested close relatives of *A. macleodii* ATCC 27126 within the genus *Alteromonas*, including *A. mediterranea* DSM 17117, a strain formerly classified as a deep ecotype of *A. macleodii*[1], and *A. mediterranea* B34, a diatom microbiome isolate[2], as well as three other *Alteromonas* strains.
- We screened other genera within the Alteromonadales, *Pseudoalteromonas* spp. and *Marinobacter* spp., with the former selected on the basis of their shared functional guild as potent copiotrophs and DOM scavengers with exoenzyme activity[3,4]. *Marinobacter adhaerens* HP15 was specifically included as a well-characterised model for diatom-bacteria interactions[5], and F3R08 as an additional strain from this “foundation genus" in phytoplankton microbiomes[6].
- *Neptunomonas* sp. 3B05 is a copiotroph also associated with dissolved organic matter degradation[7], whose inclusion extended the coverage beyond the Alteromonadales, to the Oceanospirillales.
- *Vibrio* spp. included copiotrophic environmental strains with documented associations with phytoplankton and organic particles (*V. cyclitrophicus* ZF270[8], *V. splendidus* 1A01[3], *V. cholerae* C6706[9], as well as strains selected for their known antagonistic activity towards diverse marine hosts: *V. coralliilyticus* YB2[10] to corals, V. anguillarum FS-144 to other marine bacteria[11] and *V. alginolyticus* YM4 to microalgae[12].
- *Escherichia coli* was included as a phylogenetic outgroup, an enteric bacterium not known to interact with marine phytoplankton, serving as a control to allow us to assess whether protoplast formation represents a specific response to marine bacteria or a non-specific reaction to bacterial presence *per se*.
- Several of these Gammaproteobacteria were previously identified as actively motile in seawater (ZF270, 1A01, 4B03, 3B05, A3R04, F3R08, 3D05)[13], a trait associated with

bacteria-phytoplankton interactions and potentially relevant to establishing the physical proximity required to trigger protoplast formation.

- To broaden taxonomic coverage to the Alphaproteobacteria, we included multiple Rhodobacterales, a clade well-represented in marine phytoplankton phycospheres[14]. These included the canonical diatom interactor *Ruegeria pomeroyi* DSS-3[15] and diatom phycosphere isolates *Acquicoccous* sp. Liq7*, Shimia* sp. B14[2] and *Roseovarius* sp. D2R04 and B3, with the latter isolated from *T. rotula* CCMP 3096 in our laboratory.
- *Phaeobacter inhibens* was chosen for its known antagonistic activity towards phytoplankton[16].
- A further Alphaproteobacteria strain, *Maricaulis* sp. 7BE, isolated directly from the diatom *T. pseudonana* CCMP 3367 in our laboratory, was included because a *Maricaulis* sp. was also isolated from *T. rotula*'s phycosphere[17], indicating the relevance of their association.

Whilst no panel of bacteria can exhaustively represent the diversity of marine bacteria, the strains selected represent nine families across the Alpha- and Gammaproteobacteria, known to interact with phytoplankton and spanning ecologically and functionally diverse lifestyles; therefore, providing a reasonable test of whether bacterially-induced protoplast formation is broadly distributed rather than strain-specific. Full strain details, including the descriptions above, are provided in the new Supplementary Table S2.

References

1. Ivanova, E. P. et al. Ecophysiological diversity of a novel member of the genus Alteromonas, and description of Alteromonas mediterranea sp. nov. *Antonie van Leeuwenhoek* **107**, 119–132 (2015).
2. Le Reun, N. et al. Temporal variability in the growth-enhancing effects of different bacteria within the microbiome of the diatom *Actinocyclus* sp. *Front. Microbiol.* **14**, (2023).
3. McNichol, S. M. et al. Dynamics of carbon substrate competition among heterotrophic microorganisms. *ISME J.* **18**, wrae018 (2024).
4. Clerc, E. E. et al. Strong chemotaxis by marine bacteria towards polysaccharides is enhanced by the abundant organosulfur compound DMSP. *Nat. Commun.* **14**, 8080 (2023).
5. Gärdes, A. et al. Complete genome sequence of *Marinobacter adhaerens* type strain (HP15), a diatom-interacting marine microorganism. *Stand in Genomic Sci.* **3**, 97–107 (2010).
6. Yang, X. et al. Taxonomic composition and ecophysiology of resident bacteria associated with marine phytoplankton. *Nat. Ecol. Evol.* **10**, 973–986 (2026).
7. Amarnath, K. et al. Stress-induced metabolic exchanges between complementary bacterial types underly a dynamic mechanism of inter-species stress resistance. *Nat. Commun*. **14**, 3165 (2023).
8. D'Souza, G. et al. Interspecies interactions determine growth dynamics of biopolymer-degrading populations in microbial communities. *Proc. Natl. Acad. Sci.* **120**, e2305198120 (2023).
9. King, K., Bramucci, A. R., Labbate, M., Raina, J.-B. & Seymour, J. R. Heterogeneous growth enhancement of *Vibrio cholerae* in the presence of different phytoplankton species. *Appl. Environ. Microbiol.* **88**, e01158-22 (2022).

10. Ben-Haim, Y. et al. *Vibrio coralliilyticus* sp. nov., a temperature-dependent pathogen of the coral Pocillopora damicornis. *Int. J. Syst. Evol. Microbiol.* **53**, 309–315 (2003).
11. Stubbusch, A. K. M. et al. Antagonism as a foraging strategy in microbial communities. *Science* **388**, 1214–1217 (2025).
12. Liang, R. et al. Algicidal bacteria as an eco-friendly mitigation of microalgae: an application paradigm shift to heritage conservation. *Herit. Sci.* **14**, 196 (2026).
13. Keegstra, J. M. et al. Risk–reward trade-off during carbon starvation generates dichotomy in motility endurance among marine bacteria. *Nat. Microbiol.* **10**, 1393–1403 (2025).
14. Buchan, A., LeCleir, G. R., Gulvik, C. A. & González, J. M. Master recyclers: features and functions of bacteria associated with phytoplankton blooms. *Nat. Rev. Microbiol.* **12**, 686–698 (2014).
15. Durham, B. P. et al. Recognition cascade and metabolite transfer in a marine bacteria-phytoplankton model system. *Environ. Microbiol.* **19**, 3500–3513 (2017).
16. Bramucci, A. R. & Case, R. J. *Phaeobacter inhibens* induces apoptosis-like programmed cell death in calcifying *Emiliania huxleyi. Sci. Rep.* **9**, 5215 (2019).
17. Garcia, N. S., Yung, C.-M., Davis, K. M., Rynearson, T. & Hunt, D. E. Draft Genome Sequences of Three Bacterial Isolates from Cultures of the Marine Diatom *Thalassiosira rotula. Genome Announc.* **5**, 10.1128/genomea.00316-17 (2017).

L189: ‘control’ for the bacteria, this is the bacteria dissolved in diatom culture medium?
Yes it is - thank you for highlighting this omission - we have added that clarification in the caption to Fig. 2 (“Population size over time for *A. macleodii* and *T. rotula* in co-culture and when cultured alone in diatom culture medium (“control”), on line (233).

L211 & elsewhere (like L314: biological replicates: where these independent cultures? If you used one stock culture, split into 2 x 5 cultures just prior to the experiment, it seems more accurate to refer to these as technical replicates
Technical replicates represent repeated measurements of the same biological material, whereas biological replicates derive from independent biological units. In our experiments, we used 10 independent diatom cultures in separate flasks, each initiated from independently grown stock cultures of diatom and bacteria, and treated separately; thus, these were biological replicates. This has been clarified in the methods section, which now reads “10 biological replicates, initiated from independently grown stock cultures” (line 791).

L212: why de novo (T. rotula genome available: https://www.nature.com/articles/s41597-025-04634-4)
A de novo assembly was required for the differential expression analysis because no reference genome was available at the time the reads were originally mapped (before 2025). We acknowledge that this annotated genome assembly for *T. rotula* became available in early 2025, before submission of this manuscript. However, the strain from which the genome was generated (strain FE80) is different from the one used in our study (CCMP 3096). As specified in the Methods section (line 901), to check the similarity between both assemblies, we aligned the coding DNA sequences (CDS) from our de-novo transcriptome assembly to the published genome and identified reciprocal best hits: At permissive thresholds (≥50 % identity and ≥50 %

coverage), 95 % of transcriptome-derived CDS and 80 % of genome CDS showed a best hit to the other assembly, while under more stringent criteria (≥90 % identity, ≥90 % coverage), 66 % of transcript-derived CDS still aligned to genome CDS. A summary of the genome alignment metrics is provided in Supplementary Table S2.

These results indicated substantial overlap but also showed that the assemblies were not fully concordant. Since the differential expression analysis was performed using the de novo transcriptome assembly prior to the availability of the published genome, and given the incomplete one-to-one correspondence between CDS sets, we retained the original transcriptome assembly to ensure analytical consistency.

This rationale is stated in the Methods (line 880), "Due to the lack of a reference genome for *T. rotula* at the time of analysis, *de novo* assembly of the diatom transcriptome from the dual RNA-seq data was performed".

Fig 3a: The transcriptome shows a clear diurinal pattern, this needs to be addressed or at least mentioned in the results
We thank the reviewer for highlighting this point; we have now addressed this in the results section, line 262, where we write "*A diurnal pattern was evident in both control and co-culture samples, illustrated clearly by clustering of the 0, 24, 48 and 72 h control samples, collected at the same phase of the 12:12 light:dark cycle.*"

Fig 3a: consider using a clear gradient for the time points (either color of figure complexity)
We thank the reviewer for this suggestion, the figure has been updated to use a color gradient matching that of Fig. 4a, improving visual consistency across figures.

Fig3b-4b: only a subset of genes is shown, it is not explained how this subset was chosen
We thank the reviewer for this point and agree that greater clarity was needed regarding our gene selection approach. Gene selection was data-driven rather than hypothesis-led. Following annotation and differential expression analysis, we identified the most strongly up- and downregulated genes and used their GO and KEGG annotations to define major functional response categories in both organisms, which we interpret as directly relevant to co-culture and protoplast formation. When such genes were associated with functional families or specific pathways, the differential expression of all annotated members of those pathways was systematically examined to contextualise the response and provide a pathway-level interpretation. The pathways highlighted in Figs. 3 and 4 represent the output of this structured manual curation approach.

As an exception to this data-driven approach, a small number of pathways were hypothesised *a priori* to be relevant to the phenotype, including sexual reproduction in *T. rotula* and ankyrin repeat proteins in *A. macleodii*. In both of these cases, differential expression analysis did not support the involvement of these genes in the observed phenotype, but they were nonetheless retained in the Figures to ensure transparent reporting.

The specific genes displayed in Figures 3 and 4 were selected to provide a clear and representative overview of the transcriptional response across cellular processes identified as relevant, rather than an exhaustive list. The complete differential expression results for all significantly regulated genes are provided via Figshare for transparency. We have revised the captions of Figures 3 and 4 to clarify that representative genes were selected based on their potential functional relevance to the phenotype (Fig. 3 caption, line 370, Fig. 4 caption, line 423), and we have added the full details of this manual curation approach and selection criteria to the Methods section (lines 925-934, 966-982).

L355: Whilst chemical inhibition of proteases could not be tested due to toxicity to T. rotula -> either clarify or remove statement
Statement removed.

L545: How are lysed, protoplast, intact cell distinguished with flow cytometry? Gates set based on test with each group?
We were unable to reliably distinguish lysed cells, protoplasts, and intact cells from one another in the flow-cytometry analysis. For this reason, in experiments where such distinctions were required, cylindrical cells, protoplasts, and lysed cells were distinguished by video microscopy, which allowed us to follow individual cells over 72 h, classify their morphology, and determine when lysis had occurred. This is explained in the caption of Fig. 2b.

Reviewer #2

The authors describe a fascinating interaction between the bacterium *Alteromonas macleodii* and the diatom *Thalassiosira rotula*. They use a comprehensive set of tools to infer that the attachment of the bacterium to the alga causes a sequence of events that leads to algal cell lysis. Compared to co-culture, enhanced DOC in xenic cultures can cause the same phenomenon (but to a far lesser extent). That A macleodii is not exclusively antagonistic but has repurposed its opportunistic copiotrophic traits to mediate host exploitation in this context is intriguing. I congratulate the authors on such thorough research. From the text to the visual presentation of data, the standard is excellent. There are a few areas however, that could be tightened.
We thank the Reviewer for the very positive feedback. Below, we have now addressed all of the points raised and provide a point-by-point response to the individual comments.

On the language used to describe programmed cell death. Clearly reconciling “the activation of PCD markers did not fully align with canonical pathways” on line 259, with “this marks the first evidence of PCD in T rotula” (line 454) [later on line 457, a distinct PCD pathway] If key PCD genes showed weak or non-significant expression changes (line 264-266) and “the necrosis is independent of NO- and autophagy-independent form of PCD, divergent from canonical pathways”, then I think consistency around necrotic-like or novel PCD pathway rather than simply PCD is advisable.
We appreciate the Reviewer's concern about terminology and recognize that stating "first evidence of PCD in *T. rotula*" alongside evidence that canonical phytoplankton PCD genes show weak expression requires clarification.

Programmed cell death (PCD) is an umbrella term encompassing multiple pathways, including metacaspase-dependent and autophagy-dependent death pathways, as well as programmed necrosis. To date, phytoplankton PCD research has mostly focused on characterising elements of the first two; metacaspase-dependent and autophagy-dependent death[1]. However, programmed necrosis is also a form of PCD, established across diverse eukaryotes[2,3] but not previously identified in *T. rotula* or other phytoplankton.

While key markers for the metacaspase- and autophagy-dependent PCD pathways studied in phytoplankton to date show weak expression in *T. rotula* in the co-culture studied here, this does not negate the programmed nature of the cell death we observe. Rather, our data provide evidence that *T. rotula* undergoes a form of PCD that resembles the programmed necrosis pathway described in other eukaryotes. Therefore, our claim of "first evidence of PCD in *T. rotula*" is appropriate, with the clarification that this represents necrotic-like PCD—a pathway distinct from the autophagy- or metacaspase-dependent cell death previously characterized in phytoplankton. Whilst we emphasize that programmed necrosis has not been previously identified in phytoplankton, we have been careful not to overstate the novelty of the PCD pathway itself, grounding our interpretation in existing evidence for this pathway from other eukaryotes.

We agree with the Reviewer that consistent terminology is very important, and we have now revised the manuscript comprehensively to clarify the distinction between different programmed cell death (PCD) pathways for the reader, as well as their context in *T. rotula* and phytoplankton more generally*:*

- All references to "canonical phytoplankton PCD" have been replaced with explicit pathway names ("Metacaspase-dependent PCD" and "autophagy-dependent PCD"):
    - Line 310: "*Expression of cell death markers did not align with the metacaspase-dependent or autophagy-dependent PCD pathways previously characterized in phytoplankto*n."
    - Line 316: "*These findings suggest T. rotula in co-culture with A. macleodii undergoes a form of PCD that is distinct from the metacaspase-, NO- and autophagy-dependent pathways studied in phytoplankton so far*."
    - Line 543: "*thus distinct from the metacaspase-dependent PCD pathways previously characterized in phytoplankton.*"
- We note that all references to the cell death pathway identified in *T. rotula* are also unified to "programmed necrosis", "programmed necrosis-like" or "necrotic-like PCD". We use the broader term "PCD" early in the manuscript and then consistently employ the specific terminology as soon as the evidence is established.
- We have been careful not to claim a novel PCD pathway, instead stating only that this represents the first evidence of programmed cell death in *T. rotula*, and the first evidence of programmed necrosis-like cell death in algae, a pathway characterised in other eukaryotes but not previously described in algae:
    - Line 538 "*This marks the first evidence of PCD in T. rotula"*
    - Line 319: "*T. rotula's PCD process displayed transcriptional signatures of mitochondrial ROS-linked PCD pathways, particularly programmed necrosis characterized in other eukaryotes but not previously described in algae*".
    - Line 565: "*Overall, we present evidence for the existence of a programmed necrosis pathway in T. rotula, characterised by necrotic phenotypes and molecular parallels to the pathway documented in other eukaryotes.*"

We thank the Reviewer for this suggestion, which we believe has substantially strengthened the manuscript and helped us resolve the apparent contradiction.

References

1. Bidle, K. D. Programmed Cell Death in Unicellular Phytoplankton. *Curr. Biol.* **26**, R594–R607 (2016).
2. Baines, C. Role of the mitochondrion in programmed necrosis. *Front. Physiol.* **1**, 156 (2010).
3. Azzolin, L. et al. The mitochondrial permeability transition from yeast to mammals. FEBS Lett. **584**, 2504–2509 (2010).

On describing the timing of Phases. The explanation on line 179 states T rotula's fate is determined within 9 h of exposure", and Phase 1 lasts 0-10 h, but then I see that sampling for transcriptomes was at 5 and 10 h. On line 407-9 T rotula cells first undergo growth arrest (0-10

h), rapid and irreversible protoplast formation (10-35) etc. Given samples were taken at 10 h, it would help if Phase labeling made it clear which Phase the 10 h sample was considered in, not leave it ambiguous.
Thank you for highlighting this ambiguity. We have revised our phase definitions to eliminate the overlap: Phase 1 (0–10 h), Phase 2 (11–35 h), Phase 3 (36–72 h). This ensures that all sampling timepoints and phenotypic descriptions are unambiguously assigned to a single phase, improving the overall coherence of the manuscript. We have adjusted all relevant instances throughout the manuscript.

On significance of the interaction. An area of general uncertainty that remains for me is how relevant this interaction is in the ocean. It is one thing for A. macleodii to co-occur with T. rotula in the volume of a Niskin bottle, but it is quite another for them to occur at the micrometer scale of the phycosphere.

Narrowing this uncertainty would involve such evidence as time-course quantification of A. macleodii during T. rotula blooms in the ocean or detecting A. macleodii in diatom cultures with protoplasts. In the absence of that, then the probability of such interactions in the ocean remains unknown and the language about the significance of this as an -/+ ecological interaction needs to be somewhat moderated – i.e., line 522 “the seismic impact bacterial interactions can exert on diatom ecology”.
We thank the Reviewer for raising this important point. We now provide five independent lines of evidence supporting the plausibility of the *T. rotula* - *A. macleodii* interaction in the ocean, as well as the broader environmental relevance of our findings. We agree that time-course quantification of *A. macleodii* during *T. rotula* blooms would provide strong means of assessing true ecological association, however unfortunately to our knowledge no such datasets currently exist.

1) To assess the likelihood of this interaction occurring in the ocean, we conducted a global co-occurrence analysis using the Microbe Atlas Project (MAP)[1], querying over 2 million environmental amplicon sequencing samples. The 16S rRNA sequence of *A. macleodii* ATCC 27126 and 18S rRNA sequence of *T. rotula* were queried against the MAP database using MAPseq[2], retrieving hits at a 97% identity threshold in both cases, consistent with the standardized operational taxonomic unit (OTU) definitions pre-computed by MAP. The 97% identity threshold represents a widely accepted proxy for genus level resolution in broad cross-domain co-occurrence surveys; as it does not provide sufficient resolution to discriminate at species level, results are reported at genus level. Our results demonstrate global co-occurence between *Alteromonas* and *Thalassiosira* (Extended Data Fig. 1), significantly exceeding chance expectation (Fisher’s Exact Test, $p = 1.19 \times 10^{-235}$, Odds Ratio = 3.63).

Furthermore, to understand if the organisms show a quantitative ecological association, we correlated the relative abundances (parts-per-million (ppm), $\log_{10}$-transformed), across the 252 co-occurring samples meeting minimum read-count thresholds (Methods). This analysis revealed a significant positive correlation in the relative abundances of *Alteromonas* and *Thalassiosira,* across globally distributed marine environments (Pearson r = 0.40, Spearman ρ =

0.42; *P* < 0.001, Extended Data Fig. 1b), indicating ecological association between the two taxa beyond simple co-detection. To confirm this association was not an artefact of environmental heterogeneity or chance, the observed correlation was compared against biological background null distributions, generated by correlating *Thalassiosira* against all other bacterial taxa (Extended Data Fig. 1c) and *Alteromonas* against all other eukaryotic taxa (Extended Data Fig. 1d), within the same samples. The observed correlation significantly exceeded both background distributions (empirical *P* < 0.001; Extended Data Fig. 1c, d, full data available in Supplementary Table 9), demonstrating that *Alteromonas* and *Thalassiosira* are more strongly correlated with each other than either taxon is with the broader eukaryotic or bacterial community, respectively. These analyses have been added to the manuscript (Extended Data Fig. 1; line 1345) and referenced in the results section on lines 163-177, with language carefully moderated to reflect the inherent limitations of correlative environmental data.

2) We provide a quantitative estimate of encounter frequency and experimental evidence of chemotaxis, which together suggest that, when they co-occur at environmentally relevant cell concentrations, individual *T. rotula* cells can be expected to have repeated physical contact with *A. macleodii* over 24 h, supporting the plausibility of microscale interactions between these organisms in the ocean where they are often co-detected (see point 1).

Firstly, we estimated the expected encounter frequency between a swimming bacterium and a diatom cell assuming ballistic encounters, for which the encounter kernel is

$$\beta = \pi(R_a + R_b)^2 \times U$$

where R_a and R_b are the radii of the diatom and bacterium, respectively, and U is the bacterial swimming speed (the diatom is non-motile)[3,4]. Assuming a bacterial swimming speed of 50 µm/s, a diatom radius of 10 µm, and a bacterial radius of 1 µm, the ballistic encounter kernel is 1.9×10^{-11} m^3/s. Using environmentally relevant bloom concentrations of 10^2-10^3 *T. rotula* cells/mL[5,6] and 10^4-10^5 *A. macleodii* cells/mL[7,8], this corresponds to 0.16-1.64 encounters with a *T. rotula* diatom per day per bacterium, <u>16-164 encounters with an *A. macleodii bacterium* per day per diatom</u>, and <u>1.6×10^6-1.6×10^8 *A. macleodii-T. rotula* encounters per day per liter of seawater</u> (see below for rationale for the choice of cell concentrations). We note that this is a conservative estimate, as it assumes random bacterial swimming and does not account for turbulence, both of which would increase encounter rates. We have included this quantitative estimate of ballistic encounter frequency in the Supplementary Text, and cited it in the results on line 168.

In a chemotaxis experiment that we have now performed using the *In Situ Chemotaxis Assay* (ISCA)[9] (Extended Data Fig. 3), we showed an approximately 10-fold accumulation of *A. macleodii* in wells containing solid-phase extracts of *T. rotula* exudates relative to artificial seawater controls after a 60-minute incubation. This statistically significant enrichment demonstrates chemotaxis of *A. macleodii* to chemicals exuded by *T. rotula.* As chemotaxis can increase encounter rates by up to one order of magnitude[3], this will further enhance effective

encounters beyond our ballistic estimate. The new findings of chemotaxis toward *T. rotula* exudates are included as Extended Data Fig. 3, and cited in the results on line 221.

3) Additional literature provides compelling evidence that Alteromonadaceae (including multiple *Alteromonas* strains) are dominant and persistent members of diatom phycospheres in the environment, with several strains reported in direct association with *T. rotula* and interacting at the phycosphere scale.

- Mönnich et al. (2020, *ISME J*)[10] showed that an *Alteromonas* sp. (OTU_7) is a dominant and consistent member of the core microbiome of *Thalassiosira rotula* (strain S16, a recent North Sea isolate). Their results demonstrate that this bacterium is specifically selected from diverse bacterial communities to colonize the diatom phycosphere, emerging as a regular associate even when *T. rotula* was exposed to highly diverse seawater inocula containing more than 2000 bacterial species.
- Garcia et al. (2017, *Genome Announcements*)[11] provide direct evidence for a stable, microscale association by isolating an *Alteromonas* sp. (W12) from a laboratory culture of the marine diatom *Thalassiosira rotula* CCMP3362. Amplicon sequencing showed this isolate had high prevalence within the xenic diatom culture, indicating a persistent and dominant presence. The study posits that the natural habitat for such bacteria is not the bulk oligotrophic seawater but rather the chemically enriched microenvironment associated with algal cells.
- Tran et al. (2023, *Environmental Microbiology*)[12] demonstrated that Alteromonadaceae were the dominant early colonisers in phycosphere colonisation experiments using a recently isolated *Thalassiosira rotula* strain (CCAC 8673 B) with enriched natural bacterial communities.
- Ahern et al. (2021, *PNAS*)[13] identified members of the order Alteromonadales in 80 out of 81 *Thalassiosira rotula* isolates collected across the global ocean, making it the order with the strongest and most ubiquitous partnership. Alteromonadales were also present in 100% of common garden experiments performed with 8 different *T. rotula* isolates inoculated with a standardized seawater bacterial community, from which the authors suggest the association is not merely a result of geographic proximity but a reproducible assembly process.
- Le Reun et al. (2023, *Frontiers in Microbiology*)[14] isolated *Alteromonas mediterranea* B34 from the microbiome of an *Actinocyclus* sp. diatom culture (20 days after isolation), with 100% identity to an ASV detected in the diatom's natural microbiome, demonstrating stable and persistent phycosphere association and supporting the ecological relevance of *Alteromonas*-diatom associations. Notably, B34 is a strain used in our new, extended screen of bacteria and we found it to induce significant protoplast formation in *T. rotula*.
- Yang et al. (2026, *Nature Ecology & Evolution*)[15] used single-cell isolation of diatom populations collected worldwide to profile the phycosphere community, specifically avoiding contamination from bacteria not associated with the phycosphere microenvironment. Their analysis identified *Alteromonas* as a globally distributed “foundation genus” that shows high occupancy and stability within the phycosphere of diverse diatom hosts.

These six independent studies are cited in the introduction (line 94), where we write: "*Members of the Alteromonas genus have been identified as recurrent and often dominant phycosphere companions of diatoms, including as stable associates of T. rotula in previous studies.*" We believe this body of literature provides clear support for the environmental relevance and plausibility of the interaction studied here at the phycosphere scale.

4) Amplicon sequencing of our xenic *T. rotula* CCMP 3096 cultures showed that members of the *Alteromonas* genus constitute a substantial fraction of the associated bacterial community (Supplementary Table S1): BLASTn analysis of the two dominant *Alteromonas* ASVs showed that one is 100% identical to multiple sequences of *Alteromonas macleodii* (including strain ATCC 27126), while the second ASV is identical to several *Alteromonas* sequences, including *A. macleodii*, but cannot be unambiguously resolved at the species level based on the sequenced region. The dominance of *Alteromonas*, including *A. macleodii*, in our xenic *T. rotula* CCMP 3096 culture, and their persistence without additional carbon sources since environmental isolation, demonstrates long-term coexistence and supports this as a naturally occurring association rather than an artificial pairing.

Furthermore, the presence of *Alteromonas* and *A. macleodii* strains in our xenic diatom cultures directly addresses the Reviewer's suggestion of "detecting *A. macleodii* in diatom cultures with protoplasts". Treatment of xenic *T. rotula* 3096 with 1% 2216 MB, resulted in 10% of protoplasts after 72 h, significantly above the no-treatment control, whereas the same treatment of axenic cultures produced no significant increase. This observation is consistent with DOC-mediated stimulation of resident bacteria, including *A. macleodii*, contributing to protoplast formation. It therefore demonstrates that protoplast formation can occur in the presence of naturally-associated bacterial communities, providing support for the ecological relevance of this phenomenon.

Taken together, these four lines of evidence demonstrate that the interaction between *T. rotula* and *A. macleodii* studied here represents an ecologically grounded pairing rather than an arbitrary experimental choice, and thus its investigation under controlled conditions provides mechanistic insight into an association with clear environmental relevance.

5) We emphasize that beyond the environmental prevalence of the specific *A. macleodii*–*T. rotula* pairing, this co-culture remains a scientifically valuable model system for broader phenomena in marine microbial ecology. In a new experiment we performed, we demonstrated that a large number and diversity of bacteria can induce protoplast formation in *T. rotula* (see Fig. 1e, and the response to Reviewer 1), substantially increasing the likelihood of such interactions occurring in the ocean, and strengthening the case for their environmental prevalence. Furthermore, bacterially-induced protoplast formation serves as a tractable experimental platform for investigating protoplast formation as a general and widespread stress response in diatoms.

While this evidence supports the environmental relevance of this association, we agree that our language regarding the environmental significance of this interaction should be moderated in

two regards. First, we have revised language that overstated the strength of the ecological association between *A. macleodii* and *T. rotula* specifically, so as not to speculate beyond the current evidence.

- Line 94: We replaced "*A. macleodii* is…a core member of the microbiome associated with *T. rotula*", with the more accurate context "*Members of the Alteromonas genus have been identified as recurrent and often dominant phycosphere companions of diatoms, including as stable associates of T. rotula in previous studies.*"
- Line 525: We removed "*As A. macleodii is a known member of T. rotula's microbiome*" and replaced it with the direct experimental evidence "*A. macleodii, which was detected in our xenic diatom cultures by 16S rRNA analysis (Supplementary Table S1)*".

Second, we have moderated language regarding the ecological consequences of this interaction in the ocean and we refer the Reviewer to our revised Discussion paragraph on Ecological implications (line 596-610).

References

1. Rodrigues, J. F. M. et al. The MicrobeAtlas database: Global trends and insights into Earth's microbial ecosystems. *Cell* **189**, 2092-2107.e17 (2026).
2. Rodrigues, J. F. M., Schmidt, T. S. B., Tackmann, J. & Von Mering, C. MAPseq: highly efficient k-mer search with confidence estimates, for rRNA sequence analysis. *Bioinformatics* **33**, 3808–3810 (2017).
3. Foffi, R., Brumley, D. R., Peaudecerf, F. J., Stocker, R. & Słomka, J. Slower swimming promotes chemotactic encounters between bacteria and small phytoplankton. *Proc. Natl. Acad. Sci.* **122**, e2411074122 (2025).
4. Słomka, J. et al. Encounter rates prime interactions between microorganisms. Interface Focus 13, 20220059 (2023).
5. Mikhail, S., Khalil, N. & El-Hadary, M. A new record of red tide forming species; *Heterocapsa triquetra, Gymnodinium impudicum, Heterosigma akashiwo* and *Thalassiosira rotula* in the Red Sea. *Egypt. J. Aquat. Biol. Fish.* **24**, 207–223 (2020).
6. Rynearson, T. A., Flickinger, S. A. & Fontaine, D. N. Metabarcoding Reveals Temporal Patterns of Community Composition and Realized Thermal Niches of *Thalassiosira* Spp. (Bacillariophyceae) from the Narragansett Bay Long-Term Plankton Time Series. *Biology* **9**, 19 (2020).
7. Tada, Y. et al. Differing Growth Responses of Major Phylogenetic Groups of Marine Bacteria to Natural Phytoplankton Blooms in the Western North Pacific Ocean. A*ppl Environ. Microbiol.* **77**, 4055–4065 (2011).
8. Pedler, B. E., Aluwihare, L. I., Azam, F. & Karl, D. M. Single bacterial strain capable of significant contribution to carbon cycling in the surface ocean. *Proc. Natl. Acad. Sci. USA* **111**, 7202-7207 (2014).
9. Lambert, B. S. *et al.* A microfluidics-based in situ chemotaxis assay to study the behaviour of aquatic microbial communities. *Nature Microbiology* **2**, 1344–1349 (2017).
10. Mönnich, J. *et al.* Niche-based assembly of bacterial consortia on the diatom *Thalassiosira rotula* is stable and reproducible. *ISME J.* **14**, 1614–1625 (2020).

11. Garcia, N. S., Yung, C.-M., Davis, K. M., Rynearson, T. & Hunt, D. E. Draft Genome Sequences of Three Bacterial Isolates from Cultures of the Marine Diatom *Thalassiosira rotula. Genome Announc*. **5**, e00316-17 (2017).
12. Tran, D. Q., Milke, F., Niggemann, J. & Simon, M. The diatom *Thalassiosira rotula* induces distinct growth responses and colonization patterns of Roseobacteraceae, Flavobacteria and Gammaproteobacteria. *Environ. Microbiol*. **25**, 3536–3555 (2023).
13. Ahern, O. M., Whittaker, K. A., Williams, T. C., Hunt, D. E. & Rynearson, T. A. Host genotype structures the microbiome of a globally dispersed marine phytoplankton. *Proc. Natl. Acad. Sci. USA.* **118**, e2105207118 (2021).
14. Le Reun, N. *et al.* Temporal variability in the growth-enhancing effects of different bacteria within the microbiome of the diatom *Actinocyclus* sp. *Front. Microbiol.* **14**, (2023).
15. Yang, X. *et al.* Taxonomic composition and ecophysiology of resident bacteria associated with marine phytoplankton. *Nat. Ecol. Evol.* **10**, 973–986 (2026).

I highlight a few more points below.
We provide a detailed point-by-point response to each of the issues raised below.

Line 82: what is the natural abundance of these organisms in the ocean? Their ratio is mentioned but not absolute abundance
We thank the Reviewer for this question. Unfortunately, to the best of our knowledge, no published data are currently available on the absolute concentrations of *A. macleodii* and *T. rotula* in the ocean and we can provide only approximate estimates, suggesting values on the order of 10^3 -10^5 cells/mL for *A. macleodii* (based on[1,2]) and 10^2 - 10^3 cells/mL for *T. rotula* during bloom conditions (based on[3,4]).

As noted in the manuscript, the concentrations used in our experiments were chosen to maintain an ecologically relevant ratio between the two organisms, while using absolute cell concentrations one to two orders of magnitude higher than those typically expected in the ocean. These concentrations were necessary to obtain a sufficiently synchronised response and permit our experimental analysis of co-cultures, including observation of sufficient cell numbers by microscopy and in particular generation of adequate biomass for robust transcriptomic analysis. Importantly, our dose-response experiments (see Extended Data Fig. 4, and additional response to point below) demonstrate that protoplast formation can be induced at lower bacterial concentrations; we observed 20% of *T. rotula* cells forming protoplasts 72 h after addition of 0.1% (v/v) *A. macleodii* culture into co-culture, approximately $1x10^5$ bacterial cells/mL. These observations demonstrate that protoplast formation is not restricted to the elevated bacterial concentration used in our main experiments, supporting the feasibility of this mechanism operating in the ocean. Under environmentally representative conditions[1,2], protoplast formation still occurs, but affects a lower proportion of diatom cells, and accordingly does not result in uniform population collapse. This suggests that *A. macleodii* and *T. rotula* may coexist in the environment, with only a subpopulation of diatom cells induced to form protoplasts, yet even partial induction would influence population dynamics and nutrient transfer

to the heterotrophic community. Our model co-culture system thus represents a useful tool for studying this environmentally relevant process.

We thank the reviewer for raising this point and agree it is important for understanding the ecological context of our study. Therefore, we have included the results of this dose-response experiment as Extended Data Fig. 4, and adapted our discussion to emphasize these points, lines 509-518.

References

1. Tada, Y. et al. Differing Growth Responses of Major Phylogenetic Groups of Marine Bacteria to Natural Phytoplankton Blooms in the Western North Pacific Ocean. A*ppl Environ. Microbiol.* **77**, 4055–4065 (2011).
2. Pedler, B. E., Aluwihare, L. I., Azam, F. & Karl, D. M. Single bacterial strain capable of significant contribution to carbon cycling in the surface ocean. *Proc. Natl. Acad. Sci. USA* **111**, 7202-7207 (2014).
3. Mikhail, S., Khalil, N. & El-Hadary, M. A new record of red tide forming species; *Heterocapsa triquetra, Gymnodinium impudicum, Heterosigma akashiwo* and *Thalassiosira rotula* in the Red Sea. *Egypt. J. Aquat. Biol. Fish.* **24**, 207–223 (2020).
4. Rynearson, T. A., Flickinger, S. A. & Fontaine, D. N. Metabarcoding Reveals Temporal Patterns of Community Composition and Realized Thermal Niches of *Thalassiosira* Spp. (Bacillariophyceae) from the Narragansett Bay Long-Term Plankton Time Series. *Biology* **9**, 19 (2020).

On species specificity Line 144: various other bacteria were tested against T rotula, and different diatoms against A macleodii, but what about different strains of T rotula against A macleodii? [I didn't think this cross-testing was extensive, so that's why I ask just exactly how specific it is.]
We appreciate this question about strain specificity. As shown in Fig. 1, we tested *A. macleodii* against three strains of *T. rotula* (CCMP 3096, 3264 and 3362). Though less strongly than *T. rotula* CCMP 3096, strains CCMP 3264 and 3362 also showed a significant increase in protoplast formation upon co-culture with *A. macleodii* ATCC 27126 compared to the 2216 MB control, averaging 18% and 25% of cells forming protoplasts after 72 h, respectively ($P < 0.001$, versus 2216 MB control for both diatom strains).

On replication Line 198: were the three independent replicates in the same multiwell plate? The imaging time alone would have made that problematic, but I ask because the standard deviation is small which suggests cells were coherent in their responses. Within 1 well, 100 cells needed to be found. To what extent was this randomized?
The three replicates, three separate wells, were cultured in the same multiwell plate and imaged simultaneously at 72 h (with a delay of a matter of seconds). Multiple fields were imaged per well (10X magnification) until ≥100 cells were captured. We subsequently counted every cell present in these images. Thank you for highlighting the ambiguity, we have adjusted the methods section on line 685-692, to reflect this.

Line 279 – on first mention, elaborate on AIF apoptosis inducing factor
AIF is defined on first mention at line 327 (previously line 277), where we describe that "*Alternative NADH dehydrogenases are distant homologues of mammalian apoptosis-inducing factor (AIF), whose release from mitochondria triggers ROS-linked PCD pathways in mammals, including programmed necrosis.*" This context is provided before line 330 (previously line 279).

Line 407-409. The interaction is described from the algal perspective but it involves two players, so I would prefer to see this phase description that also describes what the bacteria are doing.
We agree that the interaction involves both partners. We have revised lines 466-469 to better capture the two-sided nature of the relationship and it now reads: "*T. rotula cells first cease to divide (0-10 h), then proceed through rapid and irreversible protoplast formation (11-35 h), and subsequent cell lysis (from 36 h). Concurrently, A. macleodii directly attaches to the diatom cells and proliferates rapidly, facilitated by chemotaxis toward and utilisation of T. rotula-derived metabolites*."

Is Fig. 5 cited in the text?
Thank you for picking up on this - we have added a citation on line 466.

Line 422: What is in the culture medium 2216 MB that overlaps with the exometabolome of T rotula?
We thank the Reviewer for this question. We agree that potential overlap between the composition of 2216 MB and the exometabolome of *T. rotula* is relevant for understanding both the chemotactic behavior and nutrient metabolism of *A. macleodii*. However, we did not perform exometabolomic analyses in the present study and a detailed comparison is therefore beyond the scope of the current manuscript.

Nevertheless, several observations allow us to offer a plausible interpretation. Both 2216 MB and *T. rotula* cell lysate likely provide peptide-rich substrates as nutrient sources for *A. macleodii*. Because 2216 MB contains peptone (5 g/L) and yeast extract (1 g/L), both rich in peptide-derived compounds, and because our data suggest the involvement of bacterial proteases during the interaction, we consider it plausible that *A. macleodii* exploits at least partially overlapping nutrient classes in 2216 MB and in diatom-derived organic matter. This interpretation, however, will require direct exometabolomic analysis to confirm.

Moreover, we acknowledge that metabolic genes upregulated by *A. macleodii* could reflect responses to 2216 MB constituents, due to the 1% 2216 MB final concentration in co-culture, rather than *T. rotula*'s exometabolome alone. However, the progressive upregulation of bacterial genes associated with specific substrate metabolism as the co-culture proceeded (log2FC given to the 0h time point) suggests that at least some of these compounds were released by the diatom during co-culture, rather than being present in the medium from the start. For example, *A. macleodii* shows gene expression consistent with laminarin utilization as the co-culture progresses (Fig. 4b). Laminarin is an important storage polysaccharide in diatoms and would not be expected to be present in 2216 MB, suggesting this compound more likely reflects diatom-derived material. Future work using defined minimal media, exometabolomics, or isotope

tracing will be needed to identify which specific compounds in the algal exometabolome support bacterial growth.

Line 447: Is there a dilution of bacteria vs T rotula that was also tested as a threshold for protoplast formation?
We thank the reviewer for this point. As noted in our response to the previous point, we tested a range of *A. macleodii* inoculum concentrations, and found a dose-dependent increase in protoplast formation (Extended Data Fig. 4). *A. macleodii* culture was added into co-culture from 0.1-1% v/v, yielding starting cell concentrations of approximately $1x10^5$ - $1x10^6$ bacteria cells/mL with $1x10^5$ diatom cells/mL (bacteria:diatom ratios from 10:1 - 1:1). Protoplast formation occurred across all tested ratios and thus we did not identify a threshold below which protoplast formation is not observed. At the lowest concentration tested (0.1% v/v *A. macleodii* culture in the co-culture), only approximately 20% of *T. rotula* cells formed protoplasts after 72 h, so there was no total diatom population collapse. This suggests that at lower bacterial loads (approximately $1x10^5$ cells/mL), as might be encountered under natural environmental conditions[1,2], the interaction could result in partial protoplast induction and coexistence rather than diatom population collapse, which would nonetheless impact population dynamics and cell fate heterogeneity.

Although a bacterial cell concentration of $1x10^6$ cells/mL (1% v/v *A. macleodii* culture in co-culture) is likely higher than typically expected under environmental conditions, it was employed in our experiments to obtain a well-synchronized host response across the population. This was necessary to generate clear and interpretable results, for example, bulk transcriptomic analysis of a population in which only 20% of cells are forming protoplasts would yield a substantially diluted signal, confounding interpretation.

We also agree with the reviewer that this point is important for understanding the methodology and environmental context of our study and we have added this data as Extended Data Fig. 4, and have incorporated discussion of this context, lines 509-518.

References

1. Tada, Y. et al. Differing Growth Responses of Major Phylogenetic Groups of Marine Bacteria to Natural Phytoplankton Blooms in the Western North Pacific Ocean. A*ppl Environ. Microbiol.* **77**, 4055–4065 (2011).
2. Pedler, B. E., Aluwihare, L. I., Azam, F. & Karl, D. M. Single bacterial strain capable of significant contribution to carbon cycling in the surface ocean. *Proc. Natl. Acad. Sci. USA* **111**, 7202-7207 (2014).

From a technical standpoint, the 14 deg C temperature for diatom culture did not overlap well with the temperature used to grow bacteria. If 14 deg C was used as the incubation temperature then this must have slowed bacteria growth considerably. Did any technical complications arise?
We recognize that the 14°C incubation temperature was suboptimal for bacterial growth. However, our co-culture conditions were designed to replicate quasi-natural environmental conditions (temperatures expected in surface oceans) rather than to optimize for bacterial

growth. Furthermore, the 14°C was optimal to maintain diatom viability; increasing temperature would further stress the diatom and introduce a confounding effect. We did not observe signs of temperature stress in bacterial morphology and behaviour, or encounter any technical complications.

Figures – Padj significance shown in Fig. 3 – the asterisks are too small. An asterisk shouldn't be used for P<0.1. It is non-significant and is misleading without an explanation of why.
We have revised the statistical notation in Figures 3 and 4 to follow standard conventions: all P values ≥ 0.05 are unmarked, and significant values are marked as * $P < 0.05$, ** $P < 0.01$, *** $P < 0.001$. The asterisks have been enlarged for readability.

While the quality of the plots is high, if it was me, I would be plotting the same time points in Fig 3a and Fig 4a with the same colour codes so that there's consistency. I appreciate however that colour may have been different because of the algal vs bacteria content.
We have adjusted this figure to have a color gradient matching that of Fig. 4a, improving visual consistency across figures.

Fig. 5 is excellent – well done!
Thank you!

Please note the declaration indicated that Flow cytometry was n/a whereas it was used to enumerate T rotula
Thank you - we have adjusted this.

Line-by-line comments from Reviewer #2

44: "aligicidal" = the opposite of mutualism is?
The reviewer is correct and we have revised the text to use 'amensalism', the more precise and general ecological term, rather than the algae-specific 'algicidal'.

47: these relationships → bacteria-algal
We have revised the text to use 'bacterial-algal' instead of 'these relationships' for greater specificity and clarity.

49: ^carbon
Thank you for catching this omission; we have added carbon to the sentence.

84: heterotroph^ bacteria
We have revised the text to read heterotrophic bacteria.

92: see my comment below. cell necrosis mentioned above, and canonical PCD refuted below, so you need clear consistent language around this.
Text revised, see above response.

98: ie remains untested

We agree this would be a valuable follow-up. However, we have focused the current work on characterizing the protoplast formation process itself, and feel that characterizing particle formation and comparing sinking rates between intact *T. rotula* cells and protoplasts formed in the presence of *A. macleodii* is beyond the scope of this work.

108: 2216 MB ^media

We have revised the text.

119: but not independent of the culture medium?

Correct, formation of protoplasts was not independent of the bacterial culture medium, with 1% 2216 MB media (v/v) present in the final co-culture. However, we demonstrated that bacterial culture medium alone induced significantly fewer protoplasts than co-culture with *A. macleodii*, demonstrating that bacterial presence, and not medium components alone, drives the response. Furthermore, as discussed in our response above, bacterial medium alone did not induce significant protoplast formation in axenic cultures, suggesting that the modest formation of protoplasts observed in xenic cultures upon medium addition is bacterially mediated, rather than reflecting a direct effect of the medium on the diatom itself.

125: but still higher than in natural systems? - how do these low concentrations of DOC in culture align with ambient seawater DOC?

We thank the Reviewer for this important point. Based on the standard composition of 2216 MB, a 1% carryover corresponds to 50 mg/L peptone, 10 mg/L yeast extract, and 1 mg/L ferric citrate in the final culture. Assuming that approximately 90% of these organic components were consumed by *A. macleodii* during culture growth prior to co-culturing[1], the residual medium-derived dissolved organic carbon is estimated to be on the order of 220–270 μM C. This estimate is based on TOC conversion factors of 0.53 g C/g for protein (upper bound) and 0.44 g C/g for carbohydrate (lower bound)[2]. If only 50% of the 2216 MB was consumed during bacterial culture growth, residual carbon would be approximately five-fold higher. This is substantially higher than typical open-ocean surface DOC (~60–80 μM C)[3] and is more similar to coastal[4,5] or bloom conditions[6,7], where DOC has been reported in the range of ~100–200+ μM C, and contains a higher percentage of labile DOC.

We therefore acknowledge that the residual DOC introduced with 1% (used) 2216 MB is not negligible relative to ambient open-ocean conditions. However, it falls within the range of DOC documented in productive coastal waters and eutrophic bloom conditions. We suggest that the localized organic carbon enrichment occurring in the microenvironment surrounding *T. rotula* cells during bloom conditions could produce DOC concentrations similar to our experimental conditions. Our culture conditions therefore represent an environmentally plausible scenario for exploring *A. macleodii* interactions with *T. rotula* under nutrient-enriched conditions comparable to natural blooms. We agree with the reviewer that this point is important for understanding the environmental relevance of our findings, and we have now added this calculation to the Supplementary Text and refer to it twice in the revised manuscript:

- Line 128 (Results): "*Addition of A. macleodii from exponential-phase cultures resulted in 1% (v/v) 2216 MB medium carryover into co-cultures (L1 medium in artificial seawater), introducing DOC concentrations approaching the upper end of those documented in productive coastal and bloom conditions in the ocean (Supplementary Text).*"
- Line 518 (Discussion): "*In our experiments, bacterial inoculation introduced 1% (v/v) 2216 MB medium into the co-culture resulting in DOC concentrations relevant to productive coastal and bloom conditions*".

References

1. Goto, S., Tada, Y., Suzuki, K. & Yamashita, Y. Production and Reutilization of Fluorescent Dissolved Organic Matter by a Marine Bacterial Strain, *Alteromonas macleodii. Front. Microbiol.* **8**, (2017).
2. Rouwenhorst, R. J., Frank Jzn, J., Scheffers, W. A. & Van Dijken, J. P. Determination of protein concentration by total organic carbon analysis. *J. Biochem. Biophys. Methods* **22**, 119–128 (1991).
3. Hansell, D. A., Carlson, C. A., Repeta, D. J. & Schlitzer, R. Dissolved Organic Matter in the Ocean: A Controversy Stimulates New Insights. *Oceanography* **22**, 202–211 (2009).
4. Liu, X. et al. Effects of elevated CO2 on phytoplankton during a mesocosm experiment in the southern eutrophicated coastal water of China. *Sci. Rep.* **7**, 6868 (2017).
5. Barrón, C. & Duarte, C. M. Dissolved organic carbon pools and export from the coastal ocean. *Glob. Biogeochem. Cycles* **29**, 1725–1738 (2015).
6. Zark, M. et al. Ocean Acidification Experiments in Large-Scale Mesocosms Reveal Similar Dynamics of Dissolved Organic Matter Production and Biotransformation. *Front. Mar. Sci.* **4**, (2017).
7. Vidal-Melgosa, S. et al. Diatom fucan polysaccharide precipitates carbon during algal blooms. *Nat. Commun.* **12**, 1150 (2021).

135: PKH26 - which binds to what?

PKH26 is a lipophilic dye that binds non-covalently to the aliphatic hydrocarbon chains making up the core of the cell membrane bilayer. We have now clarified this in the figure caption (line 194) and methods section (line 633).

136: insert labels to match figure legend (T. rotula strain names)

T. rotula strain names have been inserted in the figure caption.

144: any tests with different T. rotula strains?

Yes, three strains of *T. rotula* were tested against *A. macleodii* ATCC 27126, *T. rotula* CCMP 3096, 3362 and 3264.

182: or removed

We appreciate the suggestion, but we prefer to retain 'inhibited' since our experimental approach used antibiotic treatment, which inhibits and kills bacteria without physically removing cells from the culture system.

197: = 2c: I'm impressed with the coherence of the independent replicates. Were these observations made in well plates? And were more than 3 replicates cultured for each treatment but only results from 3 reps presented?
The three independent co-cultures were maintained in separate wells of the same multiwell plate (see above response). All three replicates established for this experiment are presented; no additional replicates were cultured or omitted.

223: Does sexual reproduction happen in a/xenic culture? I don't quite see that coculture excludes other explanations
Sexual reproduction has not been reported in *T. rotula* under standard culture conditions. Our initial hypothesis was that *A. macleodii* might induce sexual reproduction, with the observed protoplasts representing auxospores, which *A. macleodii* subsequently consumes. However, our transcriptomic data do not support this hypothesis. Instead, the data more strongly support programmed necrotic-like cell death as the source of the protoplast phenotype.

226: remember these are only as time-defined as when the samples for this analysis were taken
Thank you, we agree and have adjusted the text on line 276 to highlight the observed timing is determined by our sampling intervals, not necessarily the actual onset of transcription, which now reads "Major transcriptional changes were detected as early as 5 and 10 h (Phase 1)".

233: proteostasis - explain
We appreciate the suggestion for clarity. We have revised 'proteostasis' to 'protein homeostasis'; the cellular mechanisms maintaining proper protein folding and function under stress conditions, to better convey the concept to a broader readership.

263: this needs to be reconciled with previous text that discusses PCD-like progression
Text revised, see above response.

268: PCD-like?
Text revised, see above response.

279: (AIF) what is this?
See above response.

Fig 4: asterisks should be in bigger font. They are readable for me at 200x
Thank you for highlighting this, the asterisks have been enlarged for readability at normal magnification.

408: alongside rapid bacteria growth
Thank you for this suggestion. We have added information about concurrent bacterial growth to ensure the interaction description reflects both protagonists, on line 466, where we write: " *This interaction progresses through three distinct phases (Fig. 5): T. rotula cells first cease to divide (0-10 h), then proceed through rapid and irreversible protoplast formation (11-35 h), and subsequent cell lysis (from 36 h). Concurrently, A. macleodii directly attaches to the diatom cells*

and proliferates rapidly, facilitated by chemotaxis toward and utilisation of T. rotula-derived metabolites."

411: cite Fig. 5
Now cited on line 466.

440: the ratio observed in nature, but different absolute abundances - please expand on the feasibility of this mechanism being significant in the real ocean
We refer the Reviewer to our detailed responses above regarding the environmental relevance of this interaction (see "On the significance of the interaction") and the estimated natural abundances of these organisms relative to the concentrations used in our experiments (see "Line 82: what is the natural abundance of these organisms in the ocean?").

442: what are the components of MB? do they overlap with the excretome of T. rotula?
See response above.

448: was there also effort given to diluting the bacteria and monitoring the effect on protoplast generation?
See response above.

457: but non-canonical phytoplankton PCD-like pathway?
See response above.

515: test abundance of A. macleodii in aging diatom cultures
We agree with the reviewer this would be an interesting avenue, determining whether *A. macleodii* abundance correlates with protoplast formation in aging cultures (as shown in Extended Data Fig. 2a) could provide valuable insight into the interaction under more natural conditions. However, we feel this falls beyond the scope of the current manuscript and represents an avenue for future work.

536: I would not infer 'natural' after generations in culture.
We agree. We have revised the text to replace 'natural' with 'associated,' which more accurately describes the bacterial community in this long-cultured strain, line 617.

541: temperature? might be easier to create a table showing base medium, enrichment, temperature
We have simplified the paragraph to make the culturing conditions, including temperature, clearer.

546: Culture crossed out → algal
Change incorporated.

565: no bacteria counts? what is the LOD for this approach?

We did not perform direct bacterial counts, instead bacterial decontamination was verified by transferring 100 µL of the diatom culture to 2 mL 2216 MB and confirming that there was no change in the OD600 after 24 h: this culture-based detection has an effective LOD of one viable cell as a single bacterium can divide exponentially to reach detectable OD600 within 24 h. This approach is a standard and sensitive method for validating bacterial removal.

573: I noted that there are large differences in growth temperature between T.rotula and the bacterium.
See above response.

582: ah yes! is one plate a replicate or one well?
See above response.

587: were the assessed cells located via a random number generator?
See above response.

589: If imaging took place in one well in a wellplate over 72 h, then wellplates would have needed to be prepared in a serial time-course to get enough replication. Was replication then technically done over time, not simultaneously? It's a stronger approach as it shows reproducibility.
We thank the Reviewer for raising this point, but wish to clarify that replication was not performed over time. All replicate wells were within one plate, imaged within the same 72 h experiment using automated stage movement, with only seconds between fields of view. This allowed simultaneous videos of the independent replicates to be captured across the full time course, rather than requiring separate experimental runs.

597: is this consistent with elsewhere? I thought I saw a different pore size
Thank you, this was a mistake, we have corrected the pore size to 0.45 µm throughout the manuscript.

601: I would have liked to have seen bacteria only in both compartments
The experimental readout measures the proportion of *T. rotula* cells becoming protoplasts. A bacteria-only control would yield 0% by definition (no diatom cells present), which would not inform the biological question. Our *T. rotula*-only controls directly test whether bacterial presence drives the response.

621: 1 hourly?
The delay before addition of the antibiotic, after the onset of co-culture, is given by the x axis of the graph. We have adjusted the x axis label to reflect this more clearly.

632: much larger volume compared to wellplates (2L erlenmeyer)
Although we used a larger volume (560 mL in 2L Erlenmeyer flask), we verified that culture scale did not affect the results. Aliquots from flask cultures showed equivalent protoplast proportions compared to well plate experiments, confirming consistent behavior across scales.

634: final bacteria concentration and diatom:bacteria ratio?
This data is shown in Fig. 2a. At 72 h, the bacterial concentration was 4.4x10^{7} cells/mL, while the algal concentration was 5.5x10^{4} cells/mL, representing an approximate 800-fold difference in cell abundance between bacteria and diatom.

637: seems small
Sampling 70 mL at each time point yielded adequate RNA for sequencing analysis.

643: what was the total processing time?
Processing time from harvest to RNA stabilization was minimized to less than 5 minutes, to reduce artefacts in expression and RNA degradation: the two 35 mL aliquots were centrifuged for 3 minutes and then immediately treated with ROTI Aqua-Phenol, which rapidly stabilized RNA and prevented degradation.

Reviewer #3

In the manuscript by Short et al., authors examined the interaction between the diatom *Thalassiosira rotula* and the bacteria *Alteromonas macleodii*, naturally found as member of this diatom's microbiome. They discovered a novel algicidal action of *A. macleodii* through diatom protoplast emergence from the diatom silica shell, frequently followed by cell lysis. This interaction requires direct contact between diatom and bacteria cells and is species-specific, being variable also between different strains of *A. macleodii* and *T. rotula* and strongest between *A. macleodii* ATCC 27126 and *T. rotula* CCMP 3096. A dual transcriptomic analysis revealed that *T. rotula* gene expression switches from signaling and repair pathways activation in the first phase of diatom-bacteria contact to programmed cell death activation from phase 2 onwards. For *A. macleodii*, genes responsible for motility and chemotaxis were upregulated in all phases to colonize host cells, and gene expression reveals a sequential use of different diatoms derived compounds.

Overall, results suggest that this bacterium could induce and exploit an already observed stress response mechanism in diatoms, i.e. protoplast extrusion from the frustule, to benefit from diatom-derived organic compounds. This is first evidence of bacteria inducing this stress response, thus representing a novel mechanism of action that needs to be further explored to fully disclose the extent of bacterial role in protoplast formation and eventually algicidal effectors at the basis of this interaction. Thus, the manuscript content represents a useful refinement of current knowledge on diatom-bacteria algicidal interactions.
We thank the reviewer for their positive feedback, and for recognizing the novelty of this algicidal mechanism and its relevance to understanding diatom-bacteria interactions as well as diatom stress responses more generally. We have provided a detailed, point-by-point response to each of the concerns raised by the reviewer below.

Conclusions drawn by the authors are overall well supported by the analysis performed and the methodology applied is appropriate and consistent with the literature. There are however several claims that would benefit from more in-depth analyses. This applies primarily to the transcriptomic analysis for the diatom counterpart, which in some cases appears superficial. Indeed, by randomly checking *T. rotula* differentially expressed genes, we found some inaccuracies in genes description in the text referring to Fig. 3b and related Supplementary Tables S3 and S4. In many cases, the genes listed belong to gene families or have paralogs, and while it is not expected that each gene is manually checked, caution should be used when drawing conclusions. For instance, SPO11 can be SPO11-2, specific for meiosis, or SPO11-3, also used for mitosis, therefore it would be important to specify which one is the one in the figure, and how this was determined.

Cyclins in diatoms are expanded (up to 52 members in T. pseudonana), the different family members can have different functions.

Similarly, cathepsins are of many kinds, and the meaning of their modulation is described only in a general way.

Moreover, % amino acid identity with sequences from other organisms are reported along the text but without information on the top hits or on the thresholds selected to assign identity - there is nothing in the methods - their utility for the comprehension of the data is not clear. Thus, a more accurate check of annotations and description of gene expression variation needs to be added in the results section.
We thank the reviewer for this comment and acknowledge that greater clarity was needed regarding our gene annotation and selection approach.

Annotation accuracy
Automatic transcriptome annotations were generated using multiple complementary approaches; by aligning transcripts against the NCBI and UniProt nr databases and by domain prediction using InterProScan against the InterPro families database. Additionally, orthology-based annotation using eggNOG-mapper against the EggNOG database provided KEGG orthology assignments, Gene Ontology terms, and COG functional categories. Supplementary Table S5 documents the results of these annotations for *T. rotula* genes included in Fig. 3.

These complementary approaches were deemed sufficient for all *A. macleodii* genes, and for *T. rotula* genes where genes belonged to well-conserved families with clear orthologues. However, where *T. rotula* genes belonged to expanded families with functionally distinct paralogs, or where no gene of interest was directly annotated, we performed targeted BLAST searches against characterised homologues from reference organisms, to confirm or refine functional assignments. The source of annotation assignment (whether automatic transcriptome annotation or through blast alignments) is now included in the Supplementary Tables S4, for every gene shown in Fig. 3. Revised Supplementary Table S6 contains the full BLASTp outputs; putative homologs were defined as hits satisfying an e-value $\leq$ 1e−6, an alignment coverage $\geq$50% of query length, and an amino acid identity of $\geq$40%.

Expanded families
Diatoms have many expanded gene families, and due to the lack of functional characterisation in *T. rotula*, we could not confidently distinguish between co-annotated paralogues in some of these cases i.e. multiple genes carried identical annotations and similar BLAST scores following the annotation pipeline described above. In some cases, a gene from such a family was included in Fig. 3, selected based on their expression patterns, and therefore Supplementary Table S4 now explicitly documents whether:
1) All the co-annotated genes showed a consistent expression pattern, and a representative gene was selected ("Representative of family-wide expression pattern"; Suppl. Table S4).
2) The expression patterns were heterogeneous across a family, and the selected gene was explicitly flagged as a "Selected example from heterogeneously expressed gene family" (Suppl. Table S4). We interpret strong regulation of an individual family member as indicative of pathway-level activity, even when other paralogues do not exhibit the same differential expression; accordingly, such genes are discussed conservatively in the main text, at the level of pathway regulation, without inferring specific functional consequences. For expanded families

identified through BLAST searches, the expression profiles of all homologous family members are provided in Supplementary Table S6. Expression profiles of all genes are available via Figshare.

Overall, the annotation basis and selection rationale for each *T. rotula* genes included in Fig. 3 are now documented in Supplementary Tables S4, S5 and S6. We have also expanded the description of the gene annotation workflow in the Methods section (line 925-934), including the annotation criteria and BLAST thresholds used. Additional details on our methodology for selecting genes displayed in Figures 3 and 4 are provided in our response to Reviewer 1's related comment, and are also now included in the Methods section (line 966-982), as well as the captions of Figures 3 and 4. We have added a statement to the discussion (line 564) acknowledging that "*Functional validation of the genes described here will be key to confirming and extending these findings*", reflecting our view that the transcriptomic data presented here provide a valuable foundation for future mechanistic work in *T. rotula*. We thank the reviewers for their constructive feedback. Together these revisions provide greater transparency regarding our methodology for transcriptome annotation and interpretation.

Specific gene examples given by the reviewer
SPO11**:** As the reviewer noted, our initial annotation of *T. rotula*'s transcriptome did not distinguish between SPO11-2 and SPO11-3. Following this suggestion, we performed a targeted BLAST analysis using all diatom SPO11 sequences reported by Patil et al. (2015, *BMC Genomics*)[1], with *Thalassiosira pseudonana*, *Phaeodactylum tricornutum*, *Pseudo-nitzschia multiseries*, and *Fragilariopsis cylindrus* as reference queries. Only two transcripts in the *T. rotula* transcriptome showed significant similarity to SPO11 proteins. Both exhibited their strongest matches to the *T. pseudonana* SPO11-3 homologue. Although one transcript also aligned to SPO11-2, this match was substantially weaker (approximately 30% amino acid identity compared with approximately 90% identity to Spo11-3; full BLAST details in Supplementary table S6). Based on these results, we interpret both transcripts as SPO11-3 homologues and found no evidence for the presence of a SPO11-2 homologue in the transcriptome assembly, which is consistent with reported losses of SPO11-2 in some diatom lineages[1]. Given that the *T. rotula* SPO11 sequence is more likely to play a role in vegetative cell divisions than meiotic recombination, we have therefore removed it from the heatmap in Fig. 3 and removed it from the main text. We note that this does not affect our broader interpretation of the transcriptomic data.

Cyclins: the three cyclins included in Fig. 3a, show top BLAST hits to G1/S transition cyclins in *T. pseudonana* (Ccn1: B8BVI5; Ccn2: B8CD69) and a cyclin in *T. oceanica* (Ccne: K0R150, putatively Cyc8); full blast details are available in Supplementary Table S6. Ccn1 was the top blast hit for B8BVI5 by a considerable margin (pident 41.5, evalue $5.78x10^{-59}$), which was significantly downregulated during co-culture. For B8CD69, six strong hits were identified in *T. rotula*'s transcriptome (p-ident 39.4–45.9%, e-value 5.63×10^{-30}–2.46×10^{-67}); five of the six were significantly downregulated, and the gene with the lowest adjusted p-value was selected as a representative of this trend for display in Fig. 3a (full BLAST details and expression data for all hits are provided in Supplementary Table S6). G1/S cyclins function in controlling cell cycle

progression through the G1/S checkpoint, and have undergone considerable expansion in diatoms[2], with some members potentially functioning as immediate early genes regulated by light or mitogens. Given this expansion and the absence of functional characterisation in *T. rotula*, we cannot comment on specific roles of individual family members. We therefore cautiously suggest that their downregulation may indicate cell cycle arrest, consistent with microscopic observations showing no cell divisions in *T. rotula* cells in co-culture. The revised text notes their putative role in G1/S transition while acknowledging the limitations of annotation-based functional inference in an expanded gene family, on line 288, stating "*The strong downregulation of two G1/S Cyclin (Ccn) homologs from 5 h and 30 h, may indicate cell cycle arrest, though the expansion of the cyclin family in diatoms and the absence of functional characterisation in T. rotula limits the specificity of this interpretation*."

We additionally performed targeted BLAST searches for other cell cycle regulators, including cyclin-dependent kinases (CDKs)[2], with BLAST results and expression data provided in Supplementary Table S6.

Cathepsins: we agree that Cathepsin-like proteases encompass multiple subfamilies with distinct functions. Since these genes have not been functionally characterised in *T. rotula*, we deliberately described their modulation in general terms, cautiously suggesting a possible role in PCD rather than stating it as fact. However, for one cathepsin-like gene, Cpx(i) in Fig. 3, our BLAST analysis supports a more specific assignment: The top *T. rotula* hit for the *T. pseudonana* CPX-II homologue identified in Mizrachi et al. (2025)[3] (XP_002288580.1; B8BWD8) was the transcript we designate Cpx(i) (full BLAST details in Supplementary Table S6). A strong hit to tr|B7FS80|B7FS80_PHATC, CPX1 in *P. tricornutum*[3], was also obtained. Since Mizrachi et al. demonstrated conserved function of CPX genes from *P. tricornutum* CPX1 and *C. reinhardtii* CEP12 in executing PCD in response to oxidative stress, we suggest that the *T. rotula* Cpx(i) homologue likely plays a similar role. We therefore believe the stronger interpretation for Cpx(i) is supported by the published functional data and our BLAST results.

Description of gene expression variation:

A description of gene expression variation is now presented at several levels throughout the manuscript. Temporal changes in expression are shown in the time-series heatmaps (7 time points) and described in the Results as three distinct phases of the transcriptional response. Variation in gene expression was evaluated through differential expression analyses that account for variability among biological replicates: Statistical support for differential expression is provided through the adjusted p-values for individual timepoints as well as likelihood ratio tests (LRTs), reported either in Fig. 3b, in the text, where relevant, or in Suppl. Table S3. The reported *P*-values reflect the extent to which expression differences between co-culture and the control exceed variability across the biological replicates, providing a transparent measure of reproducibility for expression of each gene.

The consistency of biological replicates is further illustrated by ordination analyses (Fig. 3a), in which samples cluster according to treatment and timepoint. To make this more explicit, we have adjusted the results section (line 260) to state "*Multivariate analysis showed samples*

clustered by timepoint, with biological replicates grouping closely together, indicating a reproducible progression of the transcriptomic response (Fig. 3a)". We hope these additions and clarifications address the reviewer's request for a clearer description of gene expression variation.

References:

1. Patil, S. *et al.* Identification of the meiotic toolkit in diatoms and exploration of meiosis-specific SPO11 and RAD51 homologs in the sexual species *Pseudo-nitzschia multistriata* and *Seminavis robusta*. *BMC Genomics* **16**, 930 (2015).
2. Huysman, M. J. et al. Genome-wide analysis of the diatom cell cycle unveils a novel type of cyclins involved in environmental signaling. *Genome Biol.* **11**, R17 (2010).
3. Mizrachi, A. *et al.* Cathepsin X is a conserved cell death protein involved in algal response to environmental stress. *Curr. Biol.* **35**, 2240-2255.e6 (2025).

Another claim which seems based on expectations rather than data is the claim that there is a growth arrest in the diatom at 10 h (stated on lines 164-165, and two more times in the manuscript). In Fig. 2a, the progression of control and treatment seems very similar, then the treated sample experiences a collapse at 24 h due to bacterial lysis. There are no data flow cytometry on the cell cycle, nor a statistical analysis to show that the treated sample is different, therefore I would modify the text unless support can be provided.
We acknowledge the reviewer's point that growth arrest is a specific biological state that we have not formally demonstrated without cell cycle analysis. Our interpretation of *T. rotula*'s growth rests not on our flow cytometry data but primarily on direct observations from video microscopy: we did not observe any *T. rotula* cells in co-culture undergoing cell division, whereas control cultures showed active cell division (Supplementary Movies 1 and 2). Therefore, to more accurately reflect our observations, we have made the following changes to the manuscript:

- Line 206 (Results): replaced "*Phase 1 (0-10 h) involved growth arrest" with "Phase 1 (0-10 h) involved cessation of cell division in T. rotula, with no cell divisions observed by time-lapse microscopy*", clarifying the basis of our conclusion.
- Line 235 (Fig. 2 caption): "*Phase 1 (0–10 h) is associated with cessation of cell division in T. rotula"*
- Line 466 (Discussion): "*T. rotula cells first cease to divide (0-10 h),*"

We believe this revised terminology more faithfully represents what was directly observed, without implying a mechanistic claim about cell cycle state that our data do not support. We note that later in the manuscript, when discussing the transcriptomic evidence of G1/S cyclin downregulation, we cautiously suggest that this is consistent with our observations from video microscopy and together may indicate cell cycle arrest.

Overall, the method section contains the main information necessary to reproduce the work performed, with the exceptions here reported:

Lines 143-147: We observed no morphological changes in T. rotula CCMP 3096 co-cultured with two other algal-associated bacteria, Ruegeria pomeroyi DSS-3 and Vibrio alginolyticus.

Likewise, A. macleodii ATCC 27126 caused no morphological changes in the diatoms Thalassiosira weissflogii CCMP 1050, Thalassiosira pseudonana CCMP 3367 or Phaeodactylum tricornutum CCMP 2560.
There is information in the methods section regarding the cultivation of these diatom species but nothing about the other bacteria tested and no results are shown in the manuscript. Moreover, more details on the reason why these two bacteria have been chosen for this experiment can be added.
We thank the reviewer for catching this oversight. We have now expanded our bacterial panel to include 25 strains in total, with results presented in the updated Fig. 1e, described in the manuscript and in our response to Reviewer 1 above. The cultivation details for all bacterial strains have been added to the Methods section (line 668), and a detailed rationale for strain selection is provided in our response to Reviewer 1 and in Supplementary Table S2.

Line 303: ROS staining with CM-H2DCFDA (Methods) showed that after 10 h of co-culture,
No information is reported in the methods section. Moreover, Fig. 3c results unclear since the symbols that should identify the different time points, or the independent replicates cited in the description, are not reported in the figure.
We thank the reviewer for pointing this out, it was not included by mistake. The Methods section has now been updated to include the description of the ROS staining with CM-H2DCFDA (line 641), as well as the other fluorescence staining methods (used in Fig. 1), In "*Fluorescent staining, microscopy and flow cytometry*" (line 632).

To improve clarity in Fig. 3c, we have revised the figure to more clearly indicate the independent replicates by using distinct symbols: This change makes the presentation of the three biological replicates and their progression over time clearer to the reader. The time points are indicated by the position along the x-axis.

Other minor revisions are here reported:

Line 228: upregulation of signaling proteins such as G protein-coupled receptor, Gpcr, and kinase tor

Supplementary Table S3 and S4 show the annotation of these genes, where it is reported that Gpcr has a 7tm_3 domain, suggesting that this gene is a class 3 Gpcr. Here again, there are many Gpcrs in diatoms. Moreover, the annotation for the gene that the authors define as TOR kinase corresponds to Raptor, one of proteins involved in the TORC1 complex together with TOR. This supports an involvement of the TOR signalling pathway,
We thank the reviewer for these further points on annotation and naming. In Fig. 3 we had used simplified gene names to aid pathway level interpretation in the absence of functional validation in *T. rotula*. We agree, however, that greater precision is warranted and have updated the relevant annotations in Fig. 3, Supplementary Tables S4 and throughout the manuscript; updated acronyms now read "Gpcr (C3)" and "Raptor", as well as "Vps13", and "RhoT". The text now reads as follows (line 277), *"upregulation of signaling proteins such as Class 3 G*

protein-coupled receptor, Gpcr (C3), proteins involved in the TORC complex (Raptor), and the kinase Pka".

Regarding GPCR, we acknowledge that this gene family is highly expanded in diatoms, as in our *T. rotula* transcriptome. This example serves as a good illustration of our gene selection and reporting approach described above; The Gpcr gene displayed in Fig. 3b has been explicitly flagged as a selected example from a heterogenously expressed gene family, rather than a representative of a uniform family-wide response (Supplementary Table S4).

It would be interesting to check if the TOR kinase is also regulated. In any case the text needs to be corrected.
TOR kinases were identified in our BLASTp searches (Supplementary Table S6), suggesting homologues exist for *T. rotula*. However, TOR kinases were not significantly upregulated in our dataset.

Line 619: Antibiotics and Inhibitors
Which are the inhibitors used? The molecules listed in the paragraph are all antibiotics.
Thank you for noticing this error: The molecules listed are all antibiotics, and we have corrected the text accordingly.

Line 654: Clean up, DNase, RT and qPCR
Are qPCR data presented in the manuscript?
qPCR was performed as a quality control step to verify the success of DNase treatment and cDNA synthesis prior to downstream analyses, and was not used to generate results presented in the manuscript. We have clarified this in the Methods section on lines 834 and 838.

Overall, the work is interesting and worthy of publication. The authors propose the necrotic-like cell death as a novel mechanism and this seems supported in large part by the cluster of genes in the Necrosis category in Fig. 3b, indeed upregulated, therefore it is fundamental to be accurate on the genes described providing a more solid account of the gene identity.
We thank the reviewer for their positive assessment and for the constructive input: We hope that the additional details provided on our gene annotation and selection methodology provide a convincing and transparent account of gene identity and the transcriptional response observed.

Reviewer #4 (Remarks to the Author):

I co-reviewed this manuscript with one of the reviewers who provided the listed reports. This is part of the Nature Communications initiative to facilitate training in peer review and to provide appropriate recognition for Early Career Researchers who co-review manuscripts.
We thank you for your contribution: We hope that reviewing this manuscript was an engaging experience, and we greatly appreciate your constructive comments, which have helped us improve the manuscript.

Line-by-line comments from PDF:

29: “exploitation” = evidence of uptake of previously intracellular material
We thank the reviewer for this comment. We note that subsequent data in the manuscript support this interpretation, specifically, the growth dynamics of *A. macleodii* in co-culture and the upregulation of genes involved in metabolising diatom-derived compounds, such as the intracellular storage compound laminarin, suggesting active uptake and utilisation of previously intracellular material.

1100: in the absence of the bacterium?
Yes these protoplasts were observed in the absence of any additional bacteria, therefore providing evidence for protoplast formation as a more general stress response in diatoms. This is now clarified in the caption of Extended Data Fig. 2, line 1371.

REVIEWERS' COMMENTS

Reviewer #1 (Remarks to the Author):
I would like to thank the authors for the substantial effort they have invested in revising the manuscript. I appreciate the many improvements that have been made, including the additional co-culture experiments, which have strengthened the study.

I particularly appreciated the addition of the assessment of the environmental plausibility of the observed interaction, which was included in response to another reviewer's comments. This discussion provides valuable context and enhances the ecological relevance of the work.

Overall, the revisions,including the slight shift in focus, have, in my view, increased the broader relevance and impact of the findings. As a result, I believe this manuscript now represents a strong contribution that is well suited for publication in this journal.

Although one could still debate whether the terminology used to describe the replicates (i.e., biological versus technical replicates) is the most appropriate, I do not consider this a significant issue and do not request any further changes to the manuscript.
We thank Reviewer 1 for their generous and encouraging assessment of the revised work. We are pleased that the additional experiments and ecological context have strengthened the manuscript to their satisfaction. We acknowledge the Reviewer's comment regarding replicate terminology and we have added explicit definitions of our biological replicates for all experiments to the Statistics & Reproducibility section of the Methods, so that readers are able to assess this classification for themselves.

Reviewer #2 (Remarks to the Author):
The authors have taken the reviewers comments seriously and should be commended for the additional work they have done before resubmitting the manuscript. I find this work sound and well supported by the data.

I have only a few minor comments on the revised version.
We thank Reviewer 2 for their positive assessment of the additional work and we are very pleased that they find the work sound and well supported by the data. We also thank the Reviewer for their very careful reading of the manuscript at all stages which has substantially improved the manuscript. We have made the relevant corrections and addressed their remaining minor comments below.

Line 312 – Spell out “S.” marinoi on first use (see line 349). Done.
Line 463 – remove full stop Done.

Line 639 – What is the full magnification (this only looks like the eyepiece magnification)
We thank the reviewer for this comment but the 20× refers to the objective lens magnification, not the eyepiece. We have clarified the methods to read: "Fluorescence imaging was performed using a Nikon Ti-E; 20× NA 0.45 Objective".
Line 646 – The sentence needs a full stop. Done.
Line 674 – Was any fixative used before SYBR Green staining?
No fixative was used; cells were stained live with SYBR Green prior to imaging.
Line 797 – was RNA from the two 35 mL replicates pooled?
We thank the reviewer for this comment, yes the two separate aliquots were pooled and we have amended the methods section to clarify this; "The supernatants were pooled and stored at −80 °C until processing of all time points."

Reviewer #3 (Remarks to the Author):
We thank the authors for their point-by-point responses to our queries and for carefully refining the manuscript, which we believe has strengthened the presented results. Overall, all our comments have been clearly addressed, and the necessary modifications have been implemented in the text. Therefore, we recommend the manuscript for publication in Nature Communications.
That said, we have a minor question out of curiosity regarding why results of the experiment examining morphological changes of other Thalassiosira species and of Phaeodactylum tricornutum when in co-culture with A. macleodii (lines 144-147) are only mentioned without accompanying figures or a 'data not shown' statement.
Another minor issue is that Extended data Fig. 2 is firstly cited before Extended data Fig. 1 (line 85 for the first and 166 for the second).
We thank Reviewer 3 for their constructive comments here and throughout the review process, which have improved the manuscript.

Regarding the first point, we thank the reviewer for flagging this and we have now included these data in the Source Data File and updated the text in the Results section to indicate this. Regarding the order of Extended Data Fig. 1 and 2, we have corrected the citation order so that the Supplementary Figures are cited in the correct order in the revised manuscript.

Reviewer #4 (Remarks to the Author):
I co-reviewed this manuscript with one of the reviewers who provided the listed reports. This is part of the Nature Communications initiative to facilitate training in peer review and to provide appropriate recognition for Early Career Researchers who co-review manuscripts.

We thank Reviewer 4 for their contribution to the review process and for their participation in Nature Communications' co-review initiative.
