# Supplementary material for: Alteromonas macleodii breaches the diatom frustule triggering protoplast emergence and programmed necrosis-like death in Thalassiosira rotula
Source: Nat Commun. 2026 Sep 26;17:11158. doi: 10.1038/s41467-026-78079-7 (PMC13710752; doi:10.1038/s41467-026-78079-7)
Supplement: Supplementary file 3 [file 41467_2026_78079_MOESM1_ESM.pdf]

Supplementary Information for

Alteromonas macleodii breaches the diatom frustule triggering protoplast emergence and programmed necrosis-like death in Thalassiosira rotula

Isobel Short[1], Clara Martínez-Pérez[1#*], Samuel Miravet-Verde[2], Shinichi Sunagawa[2], Roman Stocker[1], Uria Alcolombri[3*].

Affiliations:
[1] Institute of Environmental Engineering, Department of Civil, Environmental and Geomatic Engineering, Eidgenössische Technische Hochschule Zürich (ETHZ), Stefano-Franscini-Platz 5, 8093 Zürich, Switzerland.

[2] Institute of Microbiology and Swiss Institute of Bioinformatics, Department of Biology, Eidgenössische Technische Hochschule Zürich (ETHZ), Vladimir-Prelog-Weg 4
8093 Zürich, Switzerland.

[3] Department of Plant and Environmental Sciences, Institute of Life Sciences, The Hebrew University of Jerusalem, Jerusalem 9190401, Israel.

[#] Current address: Limnological Research Station, Department of Hydrology; University of Bayreuth, Universitätsstraße 30, Bayreuth, Germany.

* Corresponding authors: Clara Martínez-Pérez (Clara.Martinez-Perez@uni-bayreuth.de) and Uria Alcolombri (uria.alcolombri@mail.huji.ac.il).

This PDF file includes:

Supplementary Figures 1-4

Supplementary Text

Supplementary References

Supplementary Figures

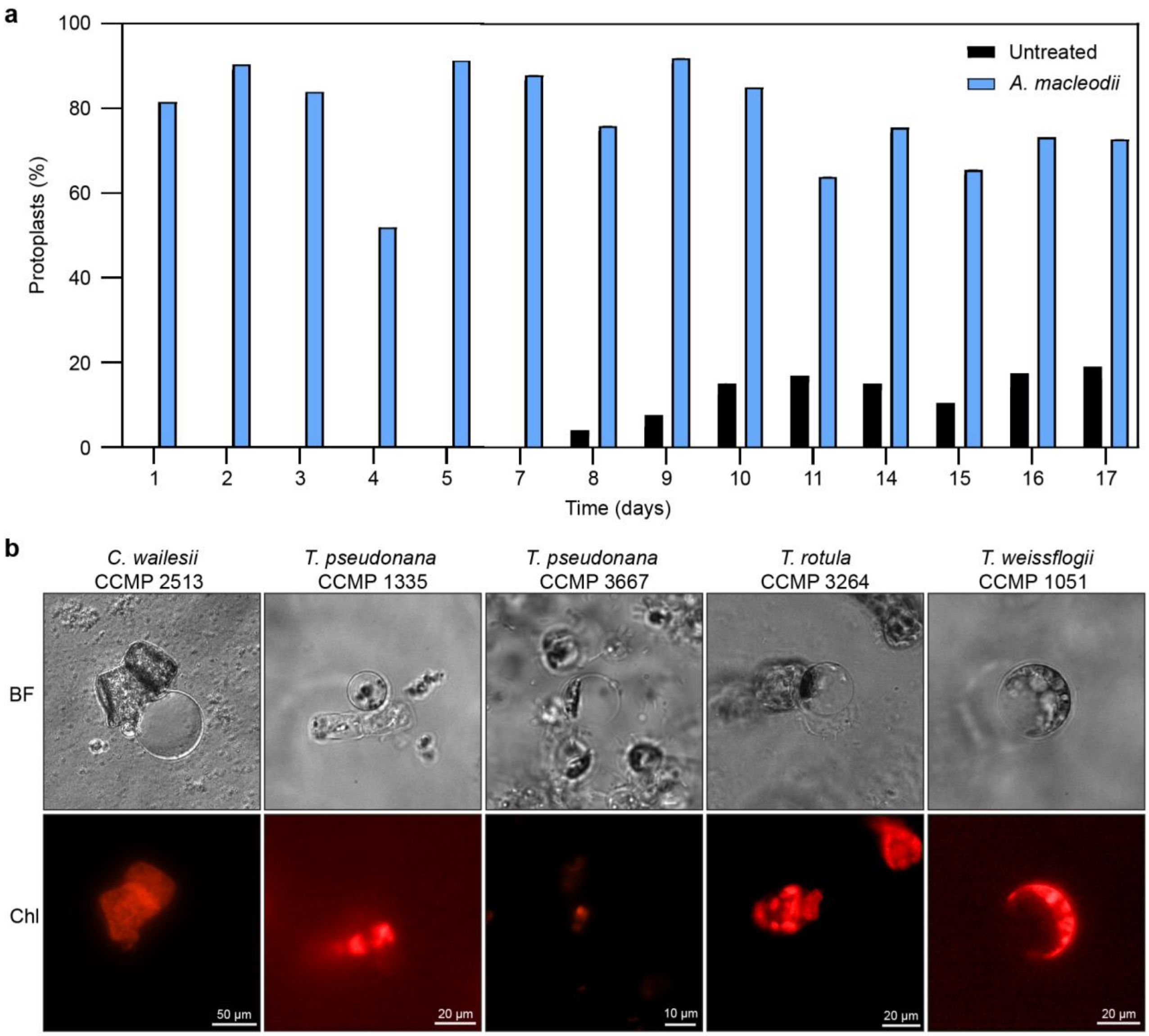

Supplementary Figure 1 | Naturally occurring protoplasts in *T. rotula* and other diatoms
a | Proportion of protoplasts (% of total diatom cells) in a *T. rotula* CCMP 3096 culture maintained for 17 days: on each day indicated, aliquots were taken and incubated for 72 h either untreated (black) or with 1% (v/v) *A. macleodii* (blue); the phenotype of 100 diatom cells was quantified by phase-contrast microscopy at the end of the 72 h incubation. Source data are provided as a Source Data file. **b** | Light microscopy (BF) and corresponding chlorophyll autofluorescence (Chl) images showing protoplasts in five diatom species without bacterial addition: *Coscinodiscus wailesii* CCMP 2513, *Thalassiosira pseudonana* CCMP 1335, *T. pseudonana* CCMP 3667, *T. rotula* CCMP 3264, and *T. weissflogii* CCMP 1051. Images are representative of the protoplast phenotype observed in each species, during a single experiment.

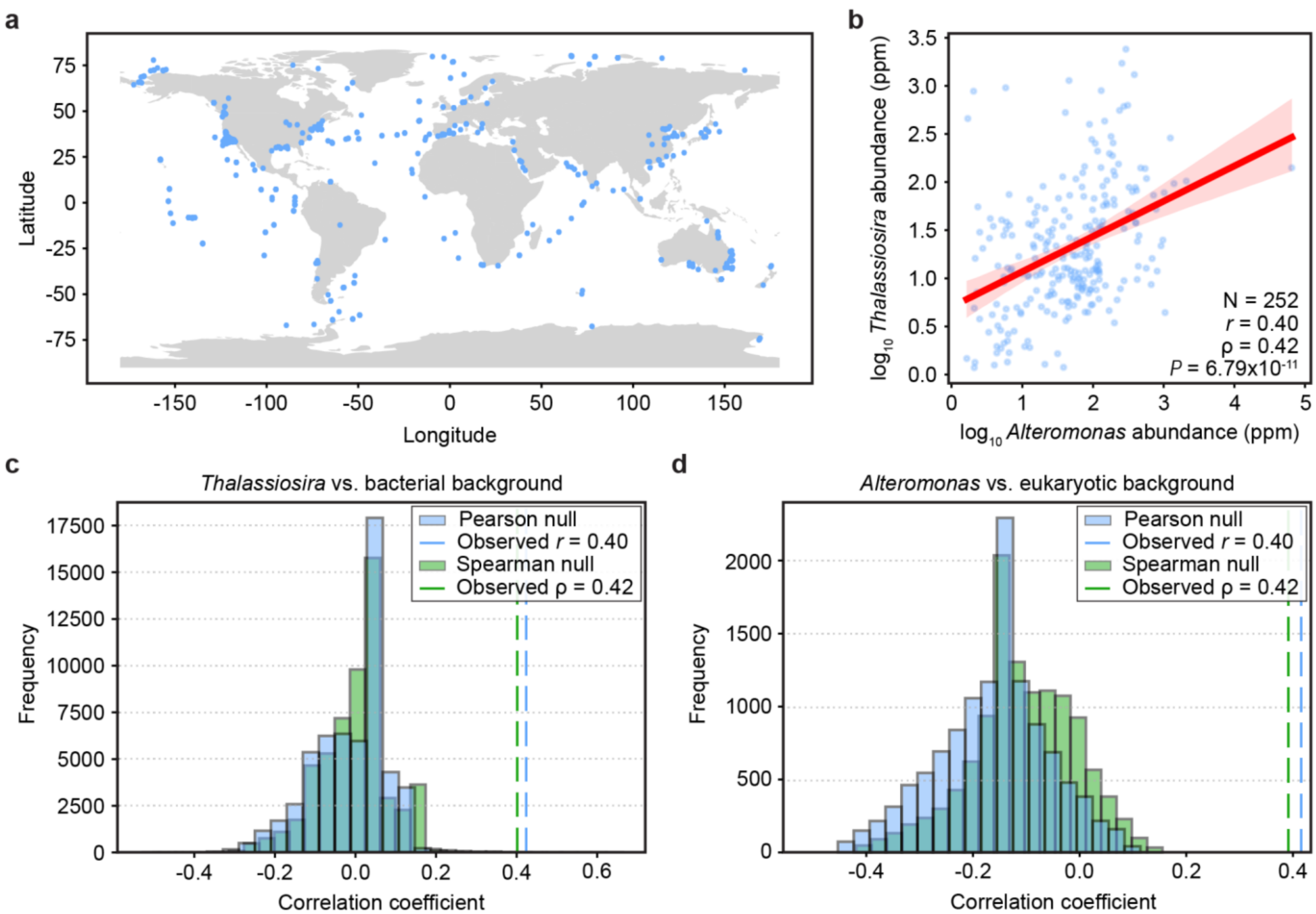

Supplementary Figure 2 | Global co-occurrence and abundance correlation between *Thalassiosira* and *Alteromonas* in Microbe Atlas.
a | Global distribution of 990 sampling sites where *Thalassiosira* and *Alteromonas* co-occur in the Microbe Atlas database (https://microbeatlas.org/; raw data provided in Supplementary Data 9). Co-occurrence was significantly enriched relative to chance (Odds Ratio = 3.63, 95% CI [3.40, 3.87], $P = 1.19^{-235}$, one-sided Fisher's exact test against the total pool of 2,056,412 MAP samples). Quantitative co-abundance analysis (b–d) was restricted to 252 samples meeting a minimum read-count threshold (Methods). **b |** Relationship between $\log_{10}$-transformed *Thalassiosira* and *Alteromonas* abundances (parts per million, ppm) across samples (N = 252), showing a positive correlation (Pearson r = 0.40, Spearman ρ = 0.42; regression line in red; *P*-value corresponds to Pearson's r). **c |** Null model assessing whether *Alteromonas* is a disproportionately strong bacterial correlate of *Thalassiosira*, relative to the bacterial background community. Null distributions of Pearson (green) and Spearman (blue) correlation coefficients were generated by correlating *Thalassiosira* abundance against all other bacterial taxa within the same samples; dashed lines of matching colour indicate observed values from panel b. The observed correlation significantly exceeds the bacterial background (empirical $P < 0.001$). **d |** Null model assessing whether *Thalassiosira* is a disproportionately strong eukaryotic correlate of *Alteromonas*, relative to the eukaryotic background community. Pearson (green) and Spearman (blue) null distributions were generated by correlating *Alteromonas* abundance

against all other eukaryotic taxa within the same samples; dashed lines indicate observed values from panel b. The observed correlation significantly exceeds the eukaryotic background (empirical $P < 0.001$). Source data are provided as a Source Data file.

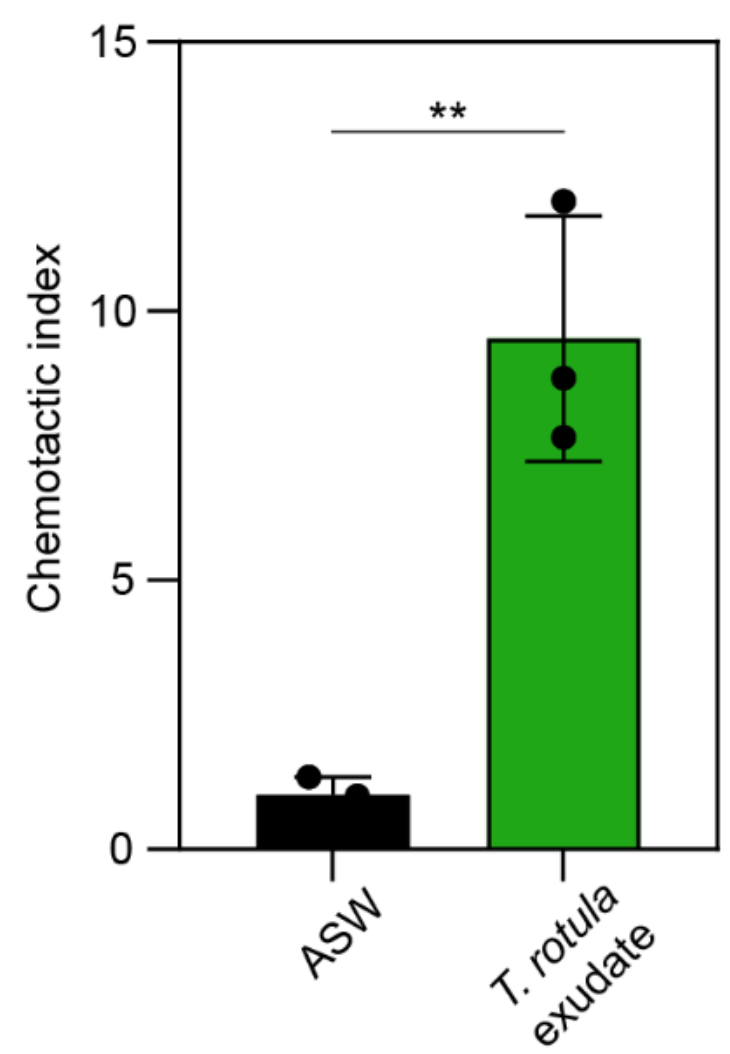

Supplementary Figure 3 | Chemotaxis of *A. macleodii* toward *T. rotula* exudates
Chemotactic index elicited by the solid phase extract of *T. rotula* exudate (Methods); chemotactic index denotes the concentration of cells in ISCA wells after a 60 min incubation, normalized by the mean concentration of cells in wells containing artificial seawater (ASW), such that a value of 1 indicates no chemotactic preference. The treatment was replicated across three independent ISCAs (n = 3), data are mean ± s.d.. Horizontal bar shows statistical significance from an unpaired two-sided t-test of log-transformed chemotactic index values; $t(4) = 6.373$, mean difference = 8.490, 95% CI [4.791, 12.19], ** $P = 0.003$. Source data are provided as a Source Data file.

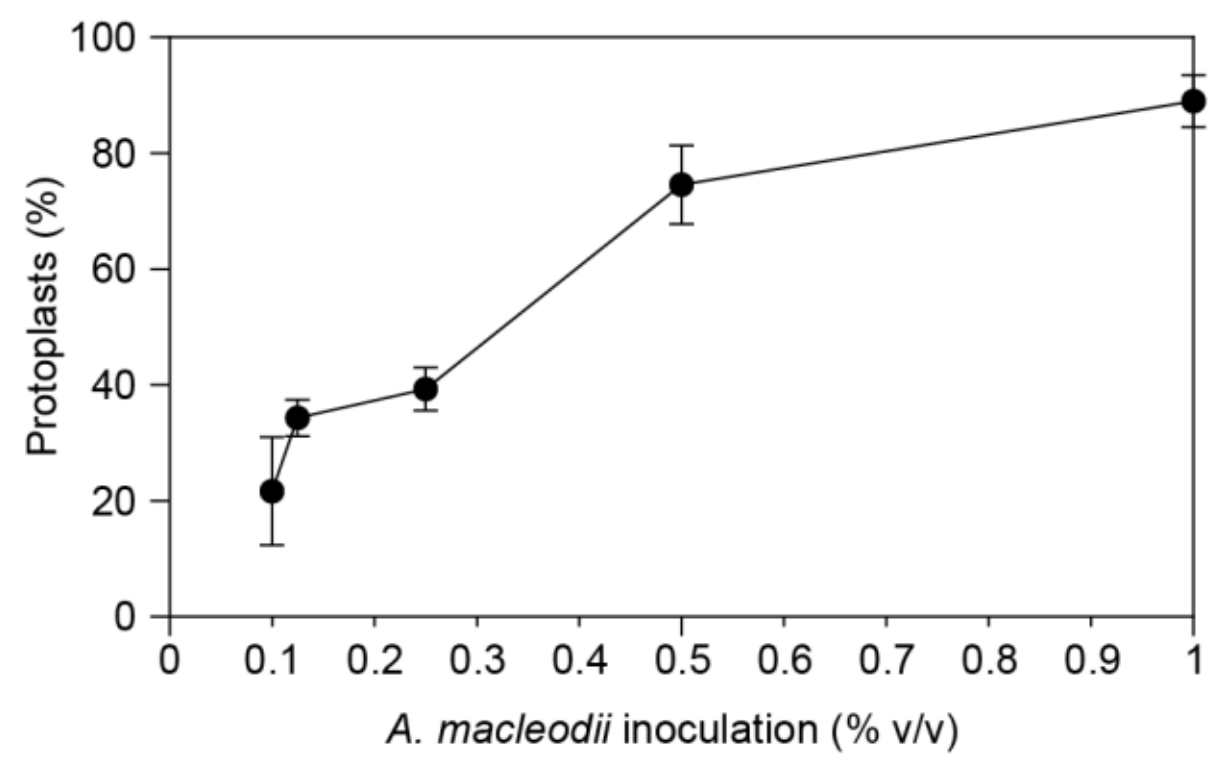

Supplementary Figure 4 | Protoplast formation in *T. rotula* across a range of bacterial inoculations
Protoplast formation (% of diatom cells) in *T. rotula* after 72 h of co-culture with *A. macleodii* across a range of bacterial inoculations; 0.1–1% v/v of the final co-culture volume, equivalent to approximately 1×10^5 - 1×10^6 cells/mL. Symbols show mean ± s.d. (n = 3 independent biological replicates), 100 diatom cells counted per replicate. Baseline protoplast formation in the absence of bacteria is shown in Fig. 1a. Source data are provided as a Source Data file.

Supplementary Text

Estimate of encounter frequency in oceanic conditions

We estimated the expected encounter frequency between a swimming bacterium and a diatom cell assuming ballistic encounters, for which the encounter kernel is

$$\beta = \pi(R_a + R_b)^2 \times U$$

where R_a and R_b are the radii of the diatom and bacterium, respectively, and U is bacterial swimming speed, whereas the diatom is assumed to be static[2,3]. Assuming a bacterial swimming speed of 50 μm/s, a diatom radius of 10 μm, and a bacterial radius of 1 μm, the ballistic encounter kernel is 1.9×10^{-14} m^3/s. Using environmentally relevant bloom concentrations of 10^2-10^3 *T. rotula* cells/mL[4,5] and 10^4-10^5 *A. macleodii* cells/mL[6,7], this corresponds to 0.16-1.64 encounters per day per bacterium, 16-164 encounters per day per diatom, and 1.6×10^6-1.6×10^8 bacteria-diatom encounters per day per liter of seawater. We note that this is a conservative estimate, as it assumes random bacterial swimming and does not account for chemotaxis or turbulence, both of which would increase encounter rates.

Calculation on DOC introduced by 1% (v/v) 2216 MB medium in co-cultures

Based on the standard composition of Marine Broth 2216, a 1% carryover corresponds to 50 mg/L peptone, 10 mg/L yeast extract, and 1 mg/L ferric citrate in the final co-culture. Assuming that approximately 90% of these organic components were consumed by *A. macleodii* during culture growth prior to co-culturing[8], the residual medium-derived dissolved organic carbon is estimated to be on the order of 220–270 μM C. This estimate is based on TOC conversion factors of 0.53 g C/g for protein (upper bound) and 0.44 g C/g for carbohydrate (lower bound)[9].

If only 50% of the 2216 MB was consumed during bacterial culture growth, residual carbon would be approximately five-fold higher.

While this is substantially higher than typical open-ocean surface DOC (~60–80 μM C)[10], it falls within the range of DOC documented in coastal[11,12] or bloom conditions[13,14] (~100–200+ μM C). We suggest that localized organic carbon enrichment occurring in the microenvironment surrounding *T. rotula* cells during bloom conditions could produce DOC concentrations similar to our experimental conditions. Our culture conditions therefore represent a relevant experimental system for exploring *A. macleodii* interactions with *T. rotula* under nutrient-enriched conditions comparable to natural blooms.

Evidence that sexual reproduction is not induced in *T. rotula*

Despite the morphological similarity of the protoplasts with diatom sexual auxospores, our results suggest that sexual reproduction is not induced by *A. macleodii*. We tested whether diatom protoplasts were formed through an aborted sexual reproduction cycle by comparing *T. rotula*'s transcriptome with marker genes of diatom sexual reproduction[15–19]. We identified four homologous genes of the minichromosome maintenance family (*Mcm2*, *Mcm3*, *Mcm6* and *Mcm7*), which play a role in eukaryotic DNA replication during both mitosis and meiosis[20], as well as homologs of meiosis-specific genes that are expressed only during sexual reproduction (*Spo11, Msh5, Sig1, Sig2* and *Sig3*)[19,21,22]. These markers were either not differentially expressed in co-culture or were strongly downregulated (Fig. 3b; Supplementary Data 4), suggesting that sexual reproduction is not induced by the bacteria and that the observed protoplasts are neither oogonia nor auxospores.

Expression of genes related to nitric oxide signaling in *T. rotula*

We found no clear evidence for nitric oxide (NO) signalling, a key regulator of PCD in other algae[23] (Fig. 3b; Supplementary Data 4). Homologs of the NO-production enzymes *TpNoa*[23] and putative nitric oxide synthases (*Nos*)[24] showed only modest upregulation (maximum log2-FC of 1.3 at 30 h), which is not consistent with strong production of NO to mediate the process. Similarly, nitrate and nitrite reductases (*Nar, Nir*) and associated transporters (*Ntrans*), capable of reductive NO production[25,26], were very strongly downregulated, with log2fold changes < -7 at 30 h (Fig. 3b).

Expression of carbohydrate metabolism genes in *A. macleodii*

A. macleodii utilized carbohydrates sparingly in co-culture, as evidenced by overall weak expression of carbohydrate-active enzymes (CAZymes), with a limited number showing sequential activation. Whilst 102 CAZymes were identified in the transcriptome, only a small number of these genes were significantly upregulated during co-culture with *T. rotula* (Supplementary Data 8). One exception was the strong and consistent upregulation of 5-keto-4-deoxyuronate isomerase (*KduI*), involved in the later stages of pectin degradation (Fig. 4b; Supplementary Data 7, log2FC > 2.9 at all time points). However, other genes from the pectin polysaccharide utilization locus (PUL) were not upregulated in co-culture, including *Pme* and *Pel* that act upstream in pectin degradation to produce the substrates of *KduI*. From Phase 2, we observed upregulation of CAZymes and transporters that indicate *A. macleodii* begins to utilise

galactose and xylose-rich EPS polysaccharides, alpha-glucans and sucrose, a metabolic intermediate in phytoplankton cells (Fig. 4b). In the third phase, particularly after 72 h, genes from the laminarin PULs[27] were upregulated (log2FC of all genes > 1.7; Fig. 4b). Laminarin is the primary storage carbohydrate in diatoms and could therefore be released when *T. rotula* cells lyse.

Upregulation of siderophore related genes in *A. macleodii*

A. macleodii increased investment in iron acquisition in Phase 1, as shown by the upregulation of genes responsible for biosynthesis of the siderophore petrobactin (*Luc*) and two siderophore specific TonB-dependent transporters (*TbdT*)[28] (Fig. 4b). A number of other neighboring iron-responsive genes[28] (*SapC, PepS, Duf* (i) and *Duf* (ii)), were strongly induced in Phase 1, including an oxidoreductase (*FeO*) and tryptophan halogenase (*THal*) (Fig. 4b). The upregulation of *Dps*, which sequesters excess iron through a bacterioferritin domain to prevent oxidative damage, is indicative of elevated intracellular iron levels. Increased iron availability likely supported higher demand for heme cofactors, as indicated by the upregulation of protoheme IX farnesyltransferase, *CtaB* (log2FC 5.7 at 24 h). Correspondingly, genes encoding heme-containing components of the respiratory electron transport chain were upregulated (Fig. 4b), including cytochrome c oxidase subunits (*CoxI–III*) and the assembly factor *CtaG*. Heme cofactors also support the activity of the nitrate assimilation enzymes *NirB*-*NasA*-*CobA*[29,30], which were likewise upregulated (Fig. 4b).

Potential for methanol oxidation by *A. macleodii*

Genes for the biosynthesis of pyrroloquinoline quinone (PQQ) were induced from 5 and 10 h in *A. macleodii*, along with two periplasmic PQQ-dependent alcohol dehydrogenases (*Adh*), all reaching log2FC > 3 at 24 h (Fig. 4b). Two additional genes, encoded in the same neighbourhood as *Adh (ii)*, were also very strongly upregulated (log2FC > 6 24-72 h); cytochrome c-550 electron acceptor *PedF*, which could serve as an electron acceptor for PQQ-dependent *Adh*, and an NAD-dependent aldehyde dehydrogenase (*Aldh* (iii)), which could catalyze oxidation of the resulting aldehyde to acid. Whilst *A. macleodii* also encodes enzymes for the downstream oxidation of formaldehyde via THF- and GSH-link pathways, these were not upregulated in co-culture. However, we detected modest upregulation of *FdhA*, a subunit of formate dehydrogenase (log2FC > 1 all timepoints), capable of oxidising formate to CO_2. This suggests that *A. macleodii* may have the capacity for complete oxidation of methanol to CO_2, though further biochemical validation is needed to determine whether methanol or other alcohols serve as the principal substrate. *A. macleodii* strain ATCC 27126 is reportedly unable to grow on methanol as a sole carbon source, consistent with the absence of a complete serine cycle[27,30]. In addition, we observed strong upregulation of several molybdo–xanthine oxidoreductase (XOR)–family genes (log_2FC > 6; Supplementary Data 8), which catalyse oxidation of diverse organic compounds, again with limited substrate annotation.

Given the strong upregulation of PQQ biosynthesis, it is also relevant that PQQ has functions beyond metabolism, including acting as a potent antioxidant and a signaling molecule, including in mitochondrial biogenesis in eukaryotic systems[31].

Supplementary References

1. Di Costanzo, F. *et al.* High-quality genome assembly and annotation of *Thalassiosira rotula* (synonym of *Thalassiosira gravida*). *Sci. Data* **12**, 310 (2025).

2. Foffi, R., Brumley, D. R., Peaudecerf, F. J., Stocker, R. & Słomka, J. Slower swimming promotes chemotactic encounters between bacteria and small phytoplankton. *Proc. Natl. Acad. Sci.* **122**, e2411074122 (2025).

3. Słomka, J. *et al.* Encounter rates prime interactions between microorganisms. *Interface Focus* **13**, 20220059 (2023).

4. Mikhail, S., Khalil, N. & El-Hadary, M. A new record of red tide forming species; *Heterocapsa triquetra, Gymnodinium impudicum, Heterosigma akashiwo* and *Thalassiosira rotula* in the Red Sea. *Egypt. J. Aquat. Biol. Fish.* **24**, 207–223 (2020).

5. Rynearson, T. A., Flickinger, S. A. & Fontaine, D. N. Metabarcoding Reveals Temporal Patterns of Community Composition and Realized Thermal Niches of *Thalassiosira* Spp. (Bacillariophyceae) from the Narragansett Bay Long-Term Plankton Time Series. *Biology* **9**, 19 (2020).

6. Tada, Y. et al. Differing Growth Responses of Major Phylogenetic Groups of Marine Bacteria to Natural Phytoplankton Blooms in the Western North Pacific Ocean. A*ppl Environ. Microbiol.* **77**, 4055–4065 (2011).

7. Pedler, B. E., Aluwihare, L. I., Azam, F. & Karl, D. M. Single bacterial strain capable of significant contribution to carbon cycling in the surface ocean. *Proc. Natl. Acad. Sci. USA* **111**, 7202-7207 (2014).

8. Goto, S., Tada, Y., Suzuki, K. & Yamashita, Y. Production and Reutilization of Fluorescent Dissolved Organic Matter by a Marine Bacterial Strain, *Alteromonas macleodii*. *Front. Microbiol.* **8**, (2017).

9. Rouwenhorst, R. J., Frank Jzn, J., Scheffers, W. A. & Van Dijken, J. P. Determination of protein concentration by total organic carbon analysis. *J. Biochem. Biophys. Methods* **22**, 119–128 (1991).

10. Hansell, D. A., Carlson, C. A., Repeta, D. J. & Schlitzer, R. Dissolved Organic Matter in the Ocean: A Controversy Stimulates New Insights. *Oceanography* **22**, 202–211 (2009).

11. Liu, X. *et al.* Effects of elevated CO2 on phytoplankton during a mesocosm experiment in the southern eutrophicated coastal water of China. *Sci. Rep.* **7**, 6868 (2017).

12. Barrón, C. & Duarte, C. M. Dissolved organic carbon pools and export from the coastal ocean. *Glob. Biogeochem. Cycles* **29**, 1725–1738 (2015).

13. Zark, M. *et al.* Ocean Acidification Experiments in Large-Scale Mesocosms Reveal Similar Dynamics of Dissolved Organic Matter Production and Biotransformation. *Front. Mar. Sci.* **4**, (2017).

14. Vidal-Melgosa, S. *et al.* Diatom fucan polysaccharide precipitates carbon during algal blooms. *Nat. Commun.* **12**, 1150 (2021).

15. Bilcke, G. *et al.* Mating type specific transcriptomic response to sex inducing pheromone in the pennate diatom *Seminavis robusta*. *ISME J.* **15**, 562–576 (2021).

16. Ferrante, M. I. *et al.* Exploring molecular signs of sex in the marine diatom *Skeletonema marinoi*. *Genes* **10**, 494 (2019).

17. Nanjappa, D., Sanges, R., Ferrante, M. I. & Zingone, A. Diatom flagellar genes and their expression during sexual reproduction in *Leptocylindrus danicus*. *BMC Genomics* **18**, 813 (2017).

18. Moore, E. R. *et al.* Morphological and transcriptomic evidence for ammonium induction of sexual reproduction in Thalassiosira pseudonana and other centric diatoms. *PLoS ONE* **12**, e0181098 (2017).

19. Patil, S. *et al.* Identification of the meiotic toolkit in diatoms and exploration of meiosis-specific SPO11 and RAD51 homologs in the sexual species *Pseudo-nitzschia multistriata* and *Seminavis robusta*. *BMC Genomics* **16**, 930 (2015).

20. Lindner, K., Gregán, J., Montgomery, S. & Kearsey, S. E. Essential Role of MCM Proteins in Premeiotic DNA Replication. *Mol. Biol. Cell* **13**, 435–444 (2002).

21. Armbrust, E. V. Identification of a New Gene Family Expressed during the Onset of Sexual Reproduction in the Centric Diatom *Thalassiosira Weissflogii*. *Appl. Environ. Microbiol.* **65**, 3121–3128 (1999).

22. Armbrust, E. V. & Galindo, H. M. Rapid Evolution of a Sexual Reproduction Gene in Centric Diatoms of the Genus *Thalassiosira*. *Appl. Environ. Microbiol.* **67**, 3501–3513 (2001).

23. Vardi, A. *et al.* A Diatom Gene Regulating Nitric-Oxide Signaling and Susceptibility to Diatom-Derived Aldehydes. *Curr. Biol.* **18**, 895–899 (2008).

24. Di Dato, V. *et al.* Transcriptome sequencing of three *Pseudo-nitzschia* species reveals comparable gene sets and the presence of Nitric Oxide Synthase genes in diatoms. *Sci. Rep.* **5**, 12329 (2015).

25. Abada, A. *et al.* Aerobic bacteria produce nitric oxide via denitrification and promote algal population collapse. *ISME J.* **17**, 1167-1183 (2023).

26. Sakihama, Y., Nakamura, S. & Yamasaki, H. Nitric Oxide Production Mediated by Nitrate Reductase in the Green Alga *Chlamydomonas reinhardtii*: an Alternative NO Production Pathway in Photosynthetic Organisms. *Plant and Cell Physiol.* **43**, 290–297 (2002).

27. Koch, H. *et al.* Biphasic cellular adaptations and ecological implications of *Alteromonas macleodii* degrading a mixture of algal polysaccharides. *ISME J.* **13**, 92–103 (2019).

28. Manck, L. E. *et al.* Petrobactin, a siderophore produced by *Alteromonas*, mediates community iron acquisition in the global ocean. *ISME J.* **16**, 358–369 (2022).

29. Diner, R. E., Schwenck, S. M., McCrow, J. P., Zheng, H. & Allen, A. E. Genetic manipulation of competition for nitrate between heterotrophic bacteria and diatoms. *Front. Microbiol.* **7**, 880 (2016).

30. Sher, D. *et al.* Collaborative metabolic curation of an emerging model marine bacterium, *Alteromonas macleodii* ATCC 27126. *PLoS ONE* **20**, e0321141 (2025).

31. Misra, H.S., Rajpurohit, Y.S. and Khairnar, N.P. Pyrroloquinoline-quinone and its versatile roles in biological processes. *J. Biosci.* **37**, 313–325 (2012).
