# Supplementary material for: Alteromonas macleodii breaches the diatom frustule triggering protoplast emergence and programmed necrosis-like death in Thalassiosira rotula
Source: Nat Commun. 2026 Sep 26;17:11158. doi: 10.1038/s41467-026-78079-7 (PMC13710752; doi:10.1038/s41467-026-78079-7)
Supplement: Supplementary file 4 [file 41467_2026_78079_MOESM2_ESM.pdf]

Description of Additional Supplementary Files

Supplementary Data 1. (Excel Workbook) Taxonomic composition of SSU rRNA reads recovered from xenic T. rotula CCMP 3096 cultures (replicates S9-S12, representing the control condition in the meta transcriptome analysis) at time 0 h. Reads were classified with PhyloFlash v3.0 against the SILVA SSU Ref NR99 database (v138.1). Values represent read abundance and relative proportion (%) of each taxonomic lineage. Due to prior rRNA depletion, results provide an approximate (non-quantitative) indication of the native bacterial community present in the diatom cultures.

Supplementary Data 2. (Excel Workbook) Details of all bacterial strains used in the co-culture screen with T. rotula, including taxonomic identity, strain designation, and culture collection accession number where applicable. Columns also include the isolation source, with corresponding references, and the rationale for inclusion in the screen, with supporting references.

Supplementary Data 3. (Excel Workbook) Quality assessment of de novo T. rotula CCMP 3096 transcriptome assembly, containing the following tabs: Assembly stats: Separate summaries are shown for the complete transcriptome assembly and for the subset of predicted CDS regions used in downstream analyses. The metrics include total sequence length (sum, in base pairs), number of sequences (n), average and maximum contig lengths (in bp), and standard N-statistics (N50–N100). Ncount shows the total number of ambiguous nucleotides (N's) in the assembly, and Gaps indicates the number of gap regions detected. BUSCO: Benchmarking Universal Single-Copy Orthologs completeness assessment of the T. rotula de novo transcriptome assembly and predicted CDS dataset, evaluated against the Stramenopiles_odb10 lineage dataset (BUSCO v5.8.3 in transcriptome mode). Report includes the counts of Complete (C), Single copy (S), Duplicated (D), Fragmented (F), and Missing (M) BUSCOs out of the total number of n ortholog groups searched. RBHs: Summary of reciprocal best-hit (RBH) analyses across varying sequence identity (50–90%) and alignment coverage (50–90%) thresholds between T. rotula de novo transcriptome derived coding sequences (CDSs) and the recently sequenced T. rotula (strain FE80)1 reference genome CDSs. Columns report Transcriptome-derived CDS hits to the genome (total number and percentage of CDSs with at least one BLASTp hit); Genome CDS hits to the transcriptome (total number and percentage of CDSs with at least one BLASTp hit); RBHs ("any": total number of reciprocal best hits, independent of thresholds and "filtered": retained after applying the specified sequence identity and coverage thresholds), shown as totals and as a percentage of all transcriptome-derived CDSs. mRNA read mapping stats: number of reads mapped to A. macleodii CDSs, to T. rotula CDSs, and the total number of high-quality mRNA reads are reported per sample. Percentages indicate the fraction of total mRNA reads assigned to A. macleodii, T.

rotula, or remaining unmapped. Mapping was performed at a nucleotide sequence identity threshold of 97% using BBMap.

Supplementary Data 4. (Excel Worksheet) Log_2 fold-change values and adjusted P-values (DESeq2 two-sided likelihood ratio test, Benjamini–Hochberg false discovery rate-adjusted) for T. rotula CDSs shown in Fig. 3, with the source of each annotation (automatic transcriptome annotation or targeted BLAST) and, where relevant, the rationale for conducting the BLAST search. For genes belonging to expanded families with co-annotated paralogues, the table also indicates whether the selected gene is representative of a family-wide expression pattern or a selected example from a heterogeneously expressed family. Complete differential expression results are available via Figshare (https://doi.org/10.6084/m9.figshare.32907734).

Supplementary Data 5. (Excel Workbook) Functional annotation of T. rotula genes included in the heatmap shown in Fig. 3. Columns summarize assigned biological processes or pathways and annotation results from multiple databases, including NCBI nr, UniProt, COG, KEGG, PFAM, and Gene Ontology (GO) terms. The last set of columns ("T. rotula genome") reports the best BLAST hits, percent identity, and KEGG/functional annotations based on the T. rotula FE80 reference genome1 . Complete functional annotation results are available via Figshare (https://doi.org/10.6084/m9.figshare.32907734)

Supplementary Data 6. (Excel Workbook) BLAST-based homology searches of T. rotula transcriptome sequences against curated query genes. BLAST searches were performed where genes belonged to expanded families with functionally distinct paralogues or lacked direct annotation, as documented for each gene shown in Fig. 3 in Supplementary Table S4. Columns report the Query sequence ID and source organism, evidence for the annotation and corresponding references, BLAST alignment statistics, transcript annotations, and log_2 fold-change values with adjusted P-values (padj_LTR; DESeq2 two-sided likelihood ratio test, Benjamini–Hochberg false discovery rate-adjusted) across all sampled timepoints. Candidate homologs used for downstream analyses were defined as hits with E-value ≤ 1e-6 , alignment coverage ≥50% of query length, and amino acid identity of ≥40%.

Supplementary Data 7. (Excel Worksheet) Log_2 fold-change values and adjusted P-values (DESeq2 two-sided likelihood ratio test, Benjamini–Hochberg false discovery rate-adjusted) for A. macleodii CDSs shown in Fig. 4 Annotation (COG category and description, KEGG ortholog identifiers) is also reported. Complete differential expression results are available via Figshare (https://doi.org/10.6084/m9.figshare.32907734).

Supplementary Data 8. (Excel Worksheet) Log_2 fold-change values and adjusted P-values (DESeq2 two-sided likelihood ratio test, Benjamini–Hochberg false discovery

rate-adjusted) for A. macleodii CDSs additionally discussed in the text. Annotation (COG category and description, KEGG ortholog identifiers and, where applicable, CAZyme classification) is also reported.

Supplementary Data 9. (Excel Worksheet) Sheet S1 contains raw abundance data for Alteromonas retrieved from the Microbe Atlas Project (MAP) database. Sheet S2 contains raw abundance data for Thalassiosira retrieved from the MAP database. Abundance values are expressed in parts per million (PPM), calculated as (reads assigned to taxon / total reads in sample) × 1,000,000. Sheet S3 contains the merged dataset of all samples in which both Alteromonas and Thalassiosira were co-detected (n = 990 samples after quality filtering), including $\log_{10}$-transformed abundance values and geographic coordinates. This dataset forms the basis for all coabundance analyses presented in Supplementary Fig. 1.

Supplementary Video 1. Time-lapse light microscopy video of four T. rotula CCMP 3096 cells undergoing the morphological change, in coculture with A. macleodii 27126. Time since the start of coculture top left, hours:minutes. Scale bar, 20 μm.

Supplementary Video 2. Time-lapse light microscopy video of three T. rotula CCMP 3096 cells undergoing the morphological change, in coculture with A. macleodii 27126. Video shows the formation of bacterial biofilm. Time since the start of co-culture top left, hours:minutes. Scale bar, 20 μm.

Supplementary Video 3. Time-lapse light microscopy video of A. macleodii 27126 chemotaxing toward a lysed T. rotula CCMP 3096 cell. The movie is displayed at a reduced playback speed for clarity; time since the start of the recording is shown at the top left (in seconds). Scale bar, 50 μm.
